# Emotion, motivation, decision-making, the orbitofrontal cortex, anterior cingulate cortex, and the amygdala

**DOI:** 10.1007/s00429-023-02644-9

**Published:** 2023-05-13

**Authors:** Edmund T. Rolls

**Affiliations:** 1 Oxford Centre for Computational Neuroscience, Oxford, UK; 2 Department of Computer Science, University of Warwick, Coventry, UK

**Keywords:** Emotion, Motivation, Reward, Human orbitofrontal cortex, Cingulate cortex, Amygdala, Ventromedial prefrontal cortex, Memory, Depression, Consciousness, Mind-brain problem, Welfare

## Abstract

The orbitofrontal cortex and amygdala are involved in emotion and in motivation, but the relationship between these functions performed by these brain structures is not clear. To address this, a unified theory of emotion and motivation is described in which motivational states are states in which instrumental goal-directed actions are performed to obtain rewards or avoid punishers, and emotional states are states that are elicited when the reward or punisher is or is not received. This greatly simplifies our understanding of emotion and motivation, for the same set of genes and associated brain systems can define the primary or unlearned rewards and punishers such as sweet taste or pain. Recent evidence on the connectivity of human brain systems involved in emotion and motivation indicates that the orbitofrontal cortex is involved in reward value and experienced emotion with outputs to cortical regions including those involved in language, and is a key brain region involved in depression and the associated changes in motivation. The amygdala has weak effective connectivity back to the cortex in humans, and is implicated in brainstem-mediated responses to stimuli such as freezing and autonomic activity, rather than in declarative emotion. The anterior cingulate cortex is involved in learning actions to obtain rewards, and with the orbitofrontal cortex and ventromedial prefrontal cortex in providing the goals for navigation and in reward-related effects on memory consolidation mediated partly via the cholinergic system.

## Introduction and aims

There have been considerable advances recently in understanding the connectivity and connections of the human orbitofrontal cortex and amygdala, and how they relate to emotion (Rolls et al. 2023a, d), but how these systems and processes are related to motivation has been much less explored. This paper shows how the brain systems involved in motivation are similar to those involved in emotion, and provides a framework for understanding how emotion and motivation are related to each other, and how similar brain systems are involved in both. This paper aims to make key advances in our understanding of how the orbitofrontal cortex and amygdala structure (anatomy and connectivity) is related to the two key functions performed by these brain regions, emotion and motivation.

To understand the neuroscience of both emotion and motivation, it is important to have a framework for understanding the relation between emotion and motivation. This paper first sets out a theory of emotion, and a framework for understanding the relation between emotion and motivation, and then considers how brain regions involved in emotion and motivation, the orbitofrontal cortex, anterior cingulate cortex, and amygdala, are involved in emotion and motivation. Special reference is made to these brain regions in primates including humans, to ensure that what is described in relevant to understanding brain systems involved in emotion and motivation in humans, and their disorders. Recent evidence about the connectivity of these systems in humans makes this paper very timely (Rolls et al. 2023a, d). A second aim is to show how emotion and its brain systems are highly adaptive from an evolutionary and gene specification perspective. The third aim is to consider where and how decisions are made about reward and emotional value, and separately about where and how decisions are made about the actions to obtain the rewards. The fourth aim is to consider some of the implications of this research for understanding brain function in health and disease; evolution to select for brain systems that respond to stimuli that encode rewards and punishers; memory and memory consolidation; and personality.

The approach taken here is new, in that it produces a unified approach to understanding emotion and motivation and their underlying brain mechanisms; in that it updates our understanding of the brain mechanisms of emotion (Rolls 2014b, 2018) by incorporating new evidence on the effective connectivity as well as the functional connectivity and the tractography of the brain systems involved in humans (Rolls et al. 2023a, d); in that it emphasises how evolution operates in part by selecting for brain reward systems that increase reproductive fitness; and in that it considers implications for understanding brain function in neurological and psychiatric states, how reward and emotional systems relate to episodic and semantic memory and memory consolidation, and welfare. The new results and understanding from taking this approach, including the advances related to new investigations of effective connectivity of the human brain, are summarised in "Conclusions and highlights".

## A theory of emotion relevant to brain systems involved in reward value and emotion

First a definition and theory of emotion and its functions are provided, and then key brain regions involved in emotion are considered, including the orbitofrontal cortex, anterior cingulate cortex, amygdala, striatum, the dopamine system, and the insula.

### A definition of emotion

**Fig. 1:**
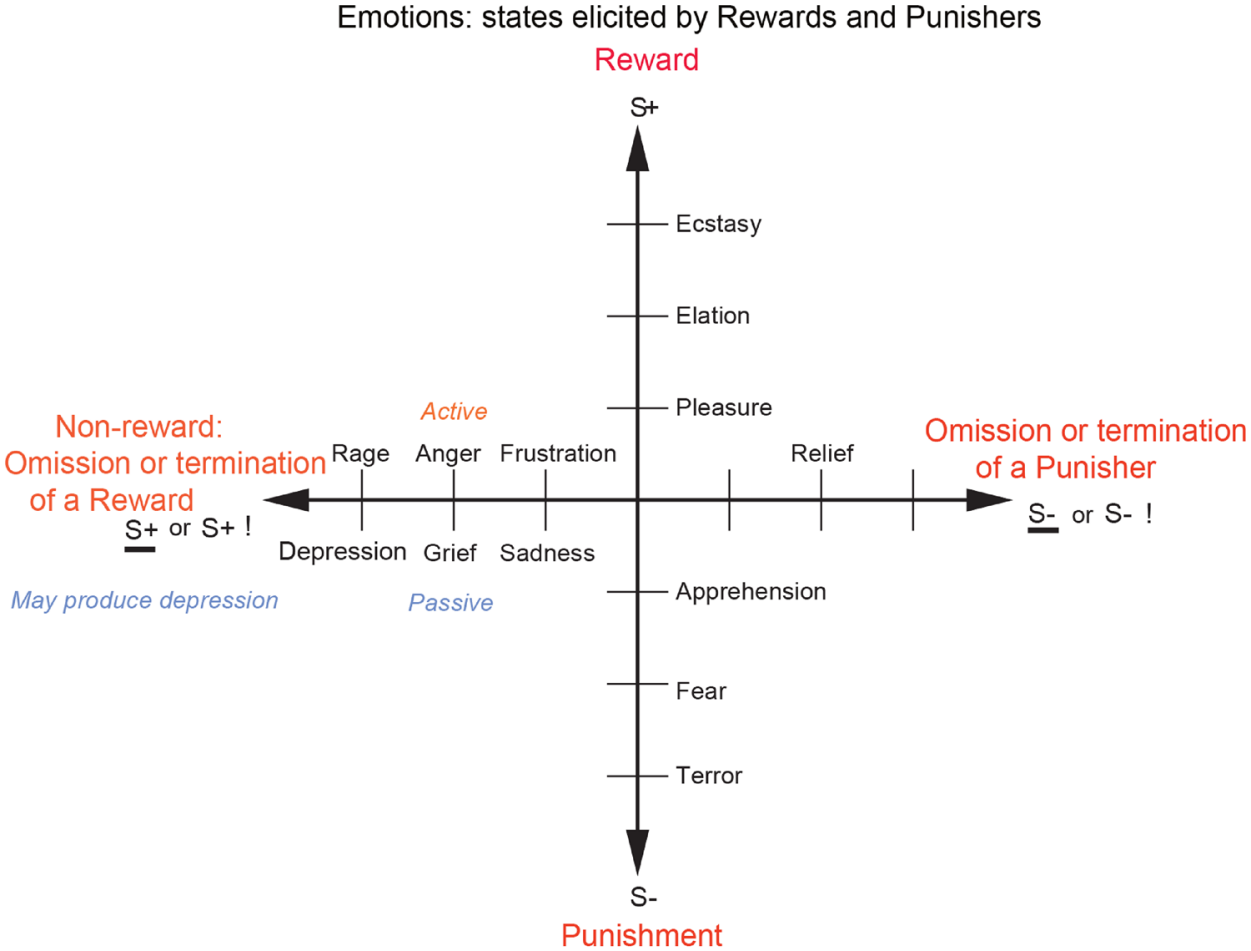
Some of the emotions associated with different reinforcement contingencies are indicated. Intensity increases away from the centre of the diagram, on a continuous scale. The classification scheme created by the different reinforcement contingencies consists with respect to the action of (1) the delivery of a reward (S+), (2) the delivery of a punisher (S−), (3) the omission of a reward (S+) (extinction) or the termination of a reward (S+ !) (time out), and (4) the omission of a punisher (S−) (avoidance) or the termination of a punisher (S−!) (escape). Note that the vertical axis describes emotions associated with the delivery of a reward (up) or punisher (down). The horizontal axis describes emotions associated with the non-delivery of an expected reward (left) or the non-delivery of an expected punisher (right). For the contingency of non-reward (horizontal axis, left) different emotions can arise depending on whether an active action is possible to respond to the non-reward, or whether no action is possible, which is labelled as the passive condition. In the passive condition, non-reward may produce depression. Frustration could include disappointment. The diagram summarizes emotions that might result for one reinforcer as a result of different contingencies. Every separate reinforcer has the potential to operate according to contingencies such as these. This diagram does not imply a dimensional theory of emotion, but shows the types of emotional state that might be produced by a specific reinforcer. Each different reinforcer will produce different emotional states, but the contingencies will operate as shown to produce different specific emotional states for each different reinforcer

A clear working definition of emotion is helpful before we consider its brain mechanisms. Emotions can usefully be defined (operationally) as states elicited by the presentation, termination or omission of rewards and punishers which have particular functions (Rolls 1999, 2000a, 2013b, 2014b, 2018). A reward is anything for which an animal (which includes humans) will work. A punisher is anything that an animal will escape from or avoid. As shown in Fig. 1, different reward/punishment contingencies are associated with different types of emotion. An example of an emotion associated with a reward might be the happiness produced by being given a particular reward, such as a pleasant touch, praise, or winning a large sum of money. An example of an emotion produced by a punisher might be fear produced by the sound of a rapidly approaching bus, or the sight of an angry expression on someone’s face. We will work to avoid such punishing stimuli. An example of an emotion produced by the omission or termination or loss of a reward is frustration or anger (if some action can be taken), or sadness (if no action can be taken). An example of an emotion produced by the omission or termination of a punisher (such as the removal of a painful stimulus, or sailing out of danger) would be relief. These examples indicate how emotions can be produced by the delivery, omission, or termination of rewarding or punishing stimuli, and go some way to indicate how different emotions could be produced and classified in terms of the rewards and punishers received, omitted, or terminated. Figure 1 summarizes some of the emotions associated with the delivery of a reward or punisher or a stimulus associated with them, or with the omission of a reward or punisher.

The subjective feelings of emotions are part of the much larger problem of consciousness (Rolls 2020). The brain bases of subjective experience are a topic of considerable current interest, not only with higher order thought (HOT) theories (Rosenthal 2004; Brown et al. 2019), but also with the higher order syntactic thought (HOST) theory of consciousness (Rolls 2007a, 2012b, 2014b, 2016c, 2018, 2020) which is more computationally specific and addresses the adaptive value of the type of processing related to consciousness; and a point made here is that the orbitofrontal cortex is at least on the route to human subjective experience of emotion and affective value (see below).

I consider elsewhere a slightly more formal definition than rewards or punishers, in which the concept of reinforcers is introduced, and it is shown that emotions can be usefully seen as states produced by instrumental reinforcing stimuli (Rolls 2014b). Instrumental reinforcers are stimuli which, if their occurrence, termination, or omission is made contingent upon the making of a response, alter the probability of the future emission of that response (Cardinal et al. 2002).

Some stimuli are unlearned (innate), “primary”, reinforcers (e.g., the taste of food if the animal is hungry, or pain). Some examples of primary reinforcers are shown in Table 1 (Rolls 2014b). There may be in the order of 100 such primary reinforcers, each specified by different genes (Rolls 2014b). Each primary reinforcer can produce a different type of affective state, for example the taste of a pleasant sweet or sweet/fat texture food such as ice cream is very different from the feel of a pleasant touch vs pain; which are all in turn very different from attraction to or love for someone. Thus different types of affective state are produced by each different primary reinforcer, and the reinforcement contingencies shown in Fig. 1 apply to each of these primary reinforcers. For example, not receiving ice cream is very different emotionally from not receiving pleasant touch.

**Table 1:** Some primary reinforcers and the dimensions of the environment to which they are tuned

**Taste**	
Salt taste	Reward in salt deficiency
Sweet	Reward in energy deficiency
Bitter	Punisher, indicator of possible poison
Sour	Punisher
Umami	Reward, indicator of protein; Produced by monosodium glutamate and inosine monophosphate
Tannic acid	Punisher: it prevents absorption of protein; found in old leaves; probably somatosensory not gustatory (Critchley and Rolls 1996)
**Odor**	
Putrefying odor	Punisher; hazard to health
Pheromones	Reward (depending on hormonal state)
**Somatosensory**	
Pain	Punisher
Touch	Reward
Grooming	Reward; to give grooming may also be a primary reinforcer
Washing	Reward
Temperature	Reward if tends to help maintain normal body temperature; otherwise punisher
**Visual**	
Snakes, etc.	Punisher for, e.g., primates
Youthfulness	Reward, associated with mate choice
Beauty, e.g., symmetry	Reward
Secondary sexual characteristics	Rewards
Face expression	Reward (e.g., smile) or punisher (e.g., threat)
Blue sky, cover, open space	Reward, indicator of safety
Flowers	Reward (indicator of fruit later in the season?)
**Auditory**	
Warning call	Punisher
Aggressive vocalization	Punisher
Soothing vocalization	Reward (part of the evolutionary history of music, which at least in its origins taps into the channels used for the communication of emotions)
**Reproduction**	
Courtship	Reward
Sexual behavior	Reward (different reinforcers, including a low waist-to-hip ratio, and attractiveness influenced by symmetry and being found attractive by members of the opposite sex)
Mate guarding	Reward for a male to protect his parental investment Jealousy results if his mate is courted by another male, because this may ruin his parental investment
Nest building	Reward (when expecting young)
Parental attachment (love)	Reward (good for the parent's genes both when the attachment is to the other parent or an infant)
Infant attachment to parents (love)	Reward (good for the infant's genes)
Crying of infant	Punisher to parents; produced to promote successful development
Power, status, wealth, resources	Attractive to females, who may benefit from resources for their offspring
	Attractive to males as they make males attractive to females
Body size	Large in males may be attractive to females as a signal for the provision of protection and of the ability of her male offspring to compete for a mate
	Small in females may be attractive to males as a neotenous sign of youth, and therefore fertility
**Other**	
Novel stimuli	Rewards (encourage animals to investigate the full possibilities of the multidimensional space in which their genes are operating)
Sleep	Reward; minimizes nutritional requirements and protects from danger
Altruism to genetic kin	Reward (kin altruism)
Altruism to other individuals	Reward while the altruism is reciprocated in a ‘tit-for-tat’ reciprocation (reciprocal altruism). Forgiveness, honesty, and altruistic punishment are some associated heuristics. May provide underpinning for some aspects of what is felt to be moral
Altruism to other individuals	Punisher when the altruism is not reciprocated
Group acceptance, reputation	Reward (social greeting might indicate this) These goals can account for why some cultural goals are pursued
Control over actions	Reward
Play	Reward
Danger, stimulation, excitement	Reward if not too extreme (adaptive because of practice?)
Exercise	Reward (keeps the body fit for action)
Mindreading	Reward; practice in reading others' minds, which might be adaptive
Solving an intellectual problem	Reward (practice in which might be adaptive)
Storing, collecting	Reward (e.g., Food)
Habitat preference, home, territory	Reward
Some responses	Reward (e.g., pecking in chickens, pigeons; adaptive because it is a simple way in which eating grain can be programmed for a relatively fixed type of environmental stimulus)
Breathing	Reward

Other stimuli may become reinforcing by associative learning, because of their association with such primary reinforcers, thereby becoming "secondary reinforcers". An example might be the sight of a painful stimulus. Brain systems that learn and unlearn these associations between stimuli or events in the environment and reinforcers are important in understanding the neuroscience and neurology of emotions, as we will see below.

This foundation has been developed (Rolls 2014b) to show how a very wide range of emotions can be accounted for, as a result of the operation of a number of factors, including the following:

1. The *reinforcement contingency* (e.g., whether reward or punishment is given, or withheld) (see Fig. 1).
2. The *intensity* of the reinforcer (see Fig. 1).
3. Any environmental stimulus might have a *number of different reinforcement associations*. (For example, a stimulus might be associated both with the presentation of a reward and of a punisher, allowing states such as conflict and guilt to arise.)
4. Emotions elicited by stimuli associated with *different primary reinforcers* will be different, as described above, with some primary reinforcers each of which will produce different affective states shown in Table 1.
5. Emotions elicited by *different secondary reinforcing stimuli* will be different from each other (even if the primary reinforcer is similar). For example, the same touch to the arm but by different people might give rise to very different emotions. Cognitive states and semantic knowledge can contribute to emotion in these ways, as well as in other ways that might arise because for example of reasoning in the rational brain system.
6. The emotion elicited can depend on whether an *active or passive behavioural response* is possible. (For example, if an active behavioural response can occur to the omission of a positive reinforcer, then anger might be produced, but if only passive behaviour is possible, then sadness, depression or grief might occur: see Fig. 1.)

By combining these six factors, it is possible to account for a very wide range of emotions, as described by Rolls (2014b). This is important: the range of emotions that can be accounted for in this way is enormous (Rolls 2014b), and is not limited (Adolphs and Anderson 2018). It is also worth noting that emotions can be produced just as much by the recall of reinforcing events as by external reinforcing stimuli; that cognitive processing (whether conscious or not) is important in many emotions, for very complex cognitive processing may be required to determine whether or not environmental events are reinforcing. Indeed, emotions normally consist of cognitive processing that analyses the stimulus, and then determines its reinforcing valence; and then an elicited affective (emotional) state or longer term mood change if the valence is positive or negative. I note that a mood or affective state may occur in the absence of an external stimulus, as in some types of depression, but that normally the mood or affective state is produced by an external stimulus, with the whole process of stimulus representation, evaluation in terms of reward or punishment, and the resulting mood or affect being referred to as emotion (Rolls 2014b).

### The functions of emotions

**Fig. 2:**
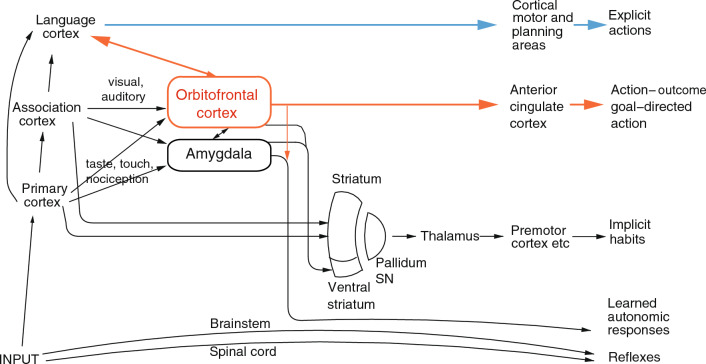
Multiple routes to the initiation of actions and responses to rewarding and punishing stimuli in primates including humans. The lowest (spinal cord and brainstem) levels in the hierarchy are involved in reflexes, including for example reflex withdrawal of a limb to a nociceptive stimulus, and unlearned autonomic responses. The second level in the hierarchy involves associative learning in the amygdala and orbitofrontal cortex between primary reinforcers such as taste, touch and nociceptive stimuli and neutral stimuli such as visual and auditory stimuli from association cortex (e.g. inferior temporal visual cortex) to produce learned autonomic and some other behavioural responses such as approach. The anteroventral viscero-autonomic insula may be one link from the orbitofrontal cortex to autonomic output. A third level in the hierarchy is the route from the orbitofrontal cortex and amygdala via the basal ganglia especially the ventral striatum to produce implicit stimulus–response habits. A fourth level in the hierarchy important in emotion is from the orbitofrontal cortex to the anterior cingulate cortex for actions that depend on the value of the goal in action–outcome learning. For this route, the orbitofrontal cortex implements stimulus-reinforcer association learning, and the anterior cingulate cortex action–outcome learning (where the outcome refers to receiving or not receiving a reward or punisher). A fifth level in the hierarchy is from the orbitofrontal cortex [and much less the amygdala (Rolls et al. 2023a)] via multiple step reasoning systems involving syntax and language. Processing at this fifth level may be related to explicit conscious states. The fifth level may also allow some top-down control of emotion-related states in the orbitofrontal cortex by the explicit processing system. Pallidum/SN—the globus pallidus and substantia nigra

**Fig. 3:**
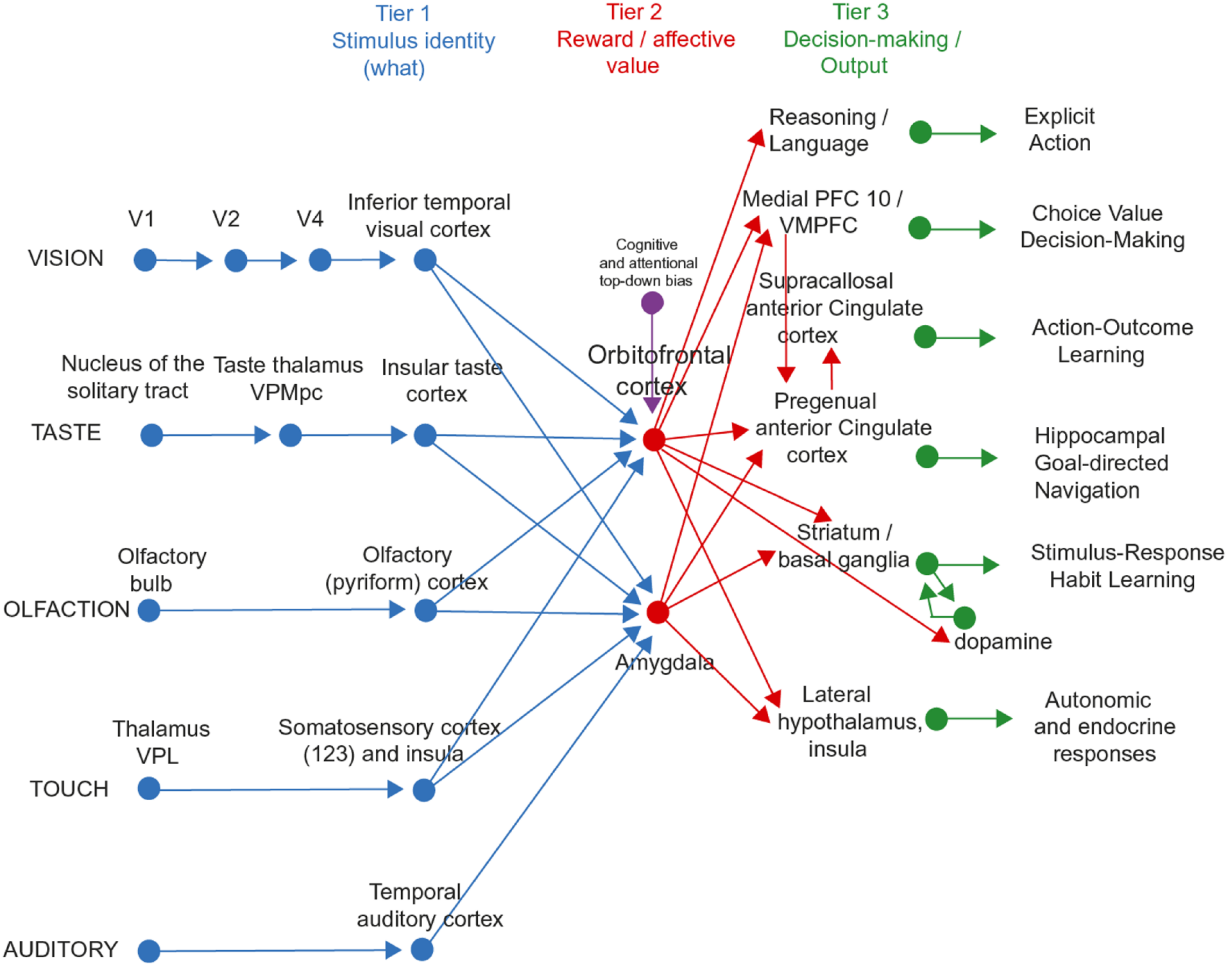
The systems level organization of the brain for emotion in primates including humans. In Tier 1, representations are built of visual, taste, olfactory and tactile stimuli that are independent of reward value and therefore of emotion. In Tier 2, reward value and emotion are represented. A pathway for top-down attentional and cognitive modulation of emotion is shown in purple. In Tier 3 actions are learned in the supracallosal (or dorsal) anterior cingulate cortex to obtain the reward values signaled by the orbitofrontal cortex and amygdala that are relayed in part via the pregenual anterior cingulate cortex and vmPFC. Decisions between stimuli of different reward value can be taken in the ventromedial prefrontal cortex, vmPFC. In Tier 3, orbitofrontal cortex inputs to the reasoning/language systems enable affective value to be incorporated and reported. In Tier 3, stimulus–response habits can also be produced using reinforcement learning. In Tier 3 autonomic responses can also be produced to emotion-provoking stimuli. Auditory inputs also reach the amygdala. V1—primary visual (striate) cortex; V2 and V4—further cortical visual areas. PFC—prefrontal cortex. The Medial PFC area 10 is part of the ventromedial prefrontal cortex (vmPFC). VPL—ventro-postero-lateral nucleus of the thalamus, which conveys somatosensory information to the primary somatosensory cortex (areas 1, 2 and 3). VPMpc—ventro-postero-medial nucleus pars parvocellularis of the thalamus, which conveys taste information to the primary taste cortex

**Fig. 4:**
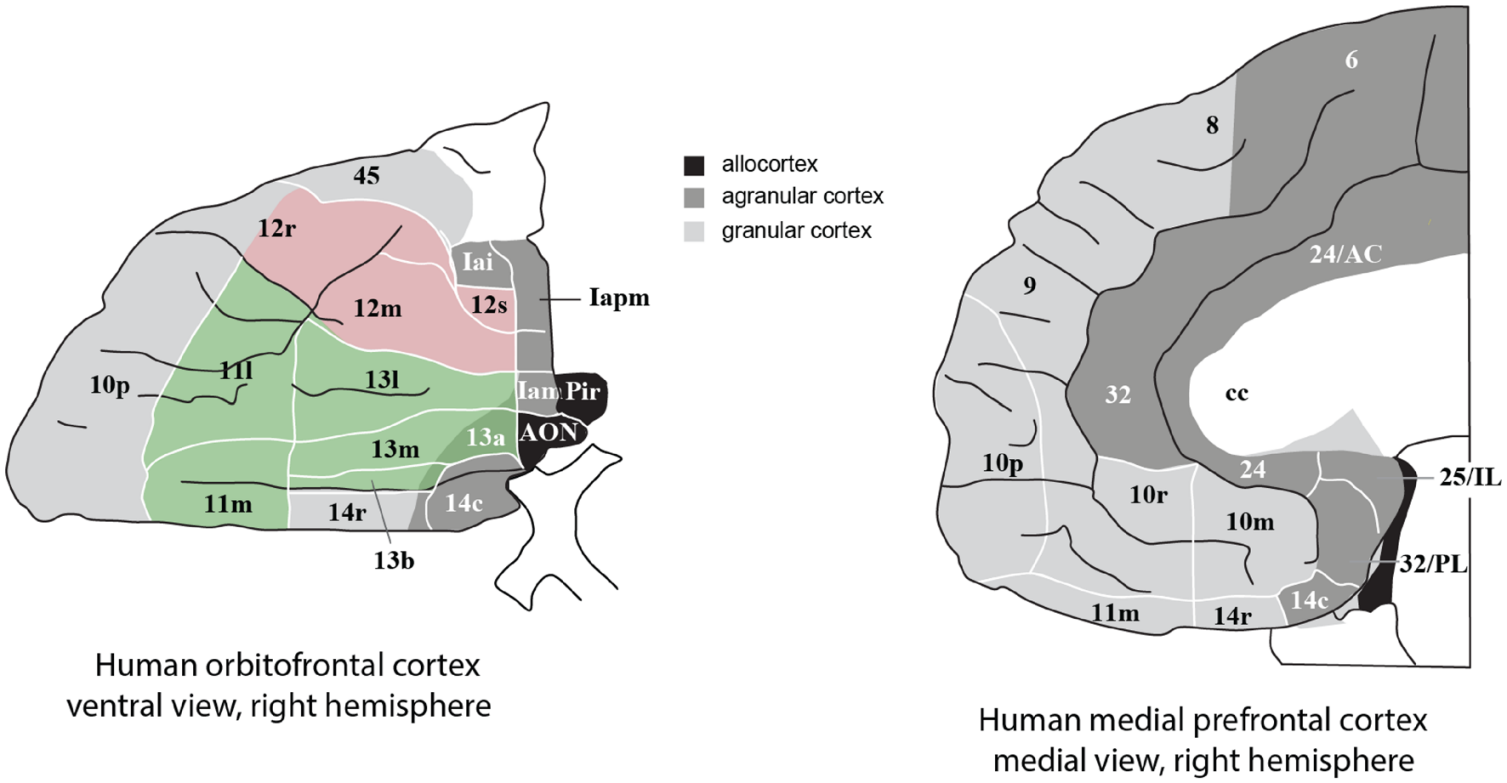
Maps of architectonic areas in the orbitofrontal cortex (left, ventral view of the brain) and medial prefrontal cortex (right, medial view of the brain) of humans. Left: the medial orbitofrontal cortex includes areas 13 and 11 (green). The lateral orbitofrontal cortex includes area 12 (red). (Area 12 is sometimes termed area 12/47 in humans. The figure shows three architectonic subdivisions of area 12.) Almost all of the human orbitofrontal cortex except area 13a is granular. Agranular cortex is shown in dark grey. The part of area 45 shown is the orbital part of the inferior frontal gyrus pars triangularis. Right: the anterior cingulate cortex includes the parts shown of areas 32, 25 (subgenual cingulate), and 24. The ventromedial prefrontal cortex includes areas 14 (gyrus rectus) 10m and 10r. AON—anterior olfactory nucleus; Iai, Ial, Iam, Iapm—subdivisions of the agranular insular cortex. (After Öngür et al. (2003) Journal of Comparative Neurology with permission of John Wiley & Sons, Inc., modified from a redrawn version by Passingham and Wise (2012).)

The most important function of emotion is as part of the processes of learning goal-directed actions to obtain rewards or avoid punishers. The first process is stimulus-reinforcer association learning; emotional states are produced as a result (Rolls 2014b). An example might be learning that the sight of a person is associated with rewards, which might produce the emotion of happiness. This process is implemented in structures such as the orbitofrontal cortex and amygdala (Figs. 2, 3, 4) (Rolls and Grabenhorst 2008; Grabenhorst and Rolls 2011; Rolls 2014b).

The second process is instrumental learning of an action made to approach and obtain the reward (an outcome of the action) or to avoid or escape from the punisher (an outcome). This is action–outcome learning, and involves brain regions such as the anterior cingulate cortex when the actions are being guided by the goals (Rushworth et al. 2011, 2012; Rolls 2014b, 2018, 2019a, 2021b, 2023d). Emotion is an integral part of this, for it is the state elicited in the first stage, by stimuli that are decoded as rewards or punishers (Rolls 2014b). The behaviour is under control of the reward value of the goal, in that if the reward is devalued, for example by feeding a food until satiety is reached, then on the very next occasion that the stimulus (the food) is offered, no action will be performed to try to obtain it (Rolls 2014b).

The striatum, rest of the basal ganglia, and dopamine system can become involved when the behaviour becomes automatic, and habit-based, that is, uses stimulus–response connections (Figs. 2, 3). In this situation, very little emotion may be elicited by the stimulus, as the behaviour has now become automated as a stimulus–response habit. For this type of learning, if the reward is devalued outside the situation, then the very next time that the stimulus is offered, the automated response is likely to be performed, providing evidence that the behaviour is no longer being guided by the reward value of the stimulus. The dopamine system is involved in this type of rather slow habit-based learning, it is thought by providing an error signal to the striatum which implements this type of habit learning (Schultz 2016c, b, 2017). The dopamine system probably receives its inputs from the orbitofrontal cortex (Rolls 2017; Rolls et al. 2023d). These brain systems are considered further below.

Other functions of emotion include the elicitation of autonomic responses, via pathways for example from the orbitofrontal cortex to the anteroventral visceral/autonomic insula and to the subgenual cingulate cortex (Critchley and Harrison 2013; Rolls 2013b, 2014b, 2019b, a; Quadt et al. 2022).

Stimuli can elicit behaviours in a number of ways via different routes to action in primates including humans, as shown in Fig. 2. An important point made by Fig. 2 is that there are multiple routes to output including to action that can be produced by stimuli that produce emotional states. Here emotional states are the states elicited by reward and punishing/non-reward stimuli, as illustrated in Fig. 1. The multiple routes are organized in a set of hierarchies, with each level in the system added later in evolution, but with all levels left in operation over the course of evolution (Rolls 2016c). The result of this is that a response such as an autonomic response to a stimulus that happens also to be rewarding might be produced by only the lower levels of the system operating, without necessarily the highest e.g. explicit levels being involved. The lowest levels in the hierarchy illustrated in Fig. 2 are involved in reflexes, including for example reflex withdrawal of a limb to a nociceptive stimulus, and autonomic responses. The second level in the hierarchy can produce learned autonomic and some other behavioural responses to for example a previously neutral visual or auditory stimulus after it has been paired with a nociceptive stimulus or with a good taste stimulus. This route involves stimulus-reinforcer learning in the amygdala and orbitofrontal cortex. A third level in the hierarchy shown in Fig. 2 is the route from the orbitofrontal cortex and amygdala via the basal ganglia especially the ventral striatum to produce implicit stimulus–response habits. A fourth level in the hierarchy that is important in emotion is from especially the orbitofrontal cortex to the anterior cingulate cortex for goal-directed action. The emotional states implemented at this level may not necessarily be conscious. A fifth level in the hierarchy shown in Fig. 2 is from the orbitofrontal cortex [and much less the amygdala (Rolls et al. 2023a)] via multiple step reasoning systems involving syntax and language, which can be associated with explicit conscious states (especially I argue if a higher order syntactic thought system for correcting lower order thoughts is involved (Rolls 2008, 2014b, 2020, 2023d), see "A reasoning, rational, route to action"). It is emphasized that each of these types of output have adaptive value in preparing individuals to deal physiologically and behaviourally with what may generally be described as emotion-provoking events.

## The neuroscience of emotion in humans and other primates

### A framework for understanding the neuroscience of emotion in humans and other primates

A framework is shown in Fig. 3, and is built on evidence from neuronal recordings, the effects of brain damage, and fMRI in humans and macaques some of which is summarized below (Rolls 2014b, 2018, 2019a, 2021b, 2023d; Rolls et al. 2020b). Part of the evidence for what is shown in Fig. 3 comes from reward devaluation, in which when the reward value is changed, for example by feeding to satiety, neural responses to stimuli are little affected in Tier 1, but decrease to zero in Tier 2. Part of the evidence comes from the learning of associations between stimuli and reward value, which occurs mainly in Tier 2. Part of the evidence comes from the effects of brain damage on emotion, which occur primarily after damage to the orbitofrontal cortex and amygdala in Tier 2, and the cingulate cortex in Tier 3 (Rolls 2021c). The organization of reward value processing and therefore emotion in the rodent brain is very different (Rolls 2019b, 2021b, 2023d), and a brief summary about this is provided in "Brain systems for emotion and motivation in primates including humans compared to those in rodents".

In the context of what is shown in Fig. 3, the focus next is on key brain areas involved in emotion in humans and other primates, the orbitofrontal cortex, anterior cingulate cortex, and amygdala.

### The orbitofrontal cortex

#### The connections and connectivity of the orbitofrontal cortex

**Fig. 5:**
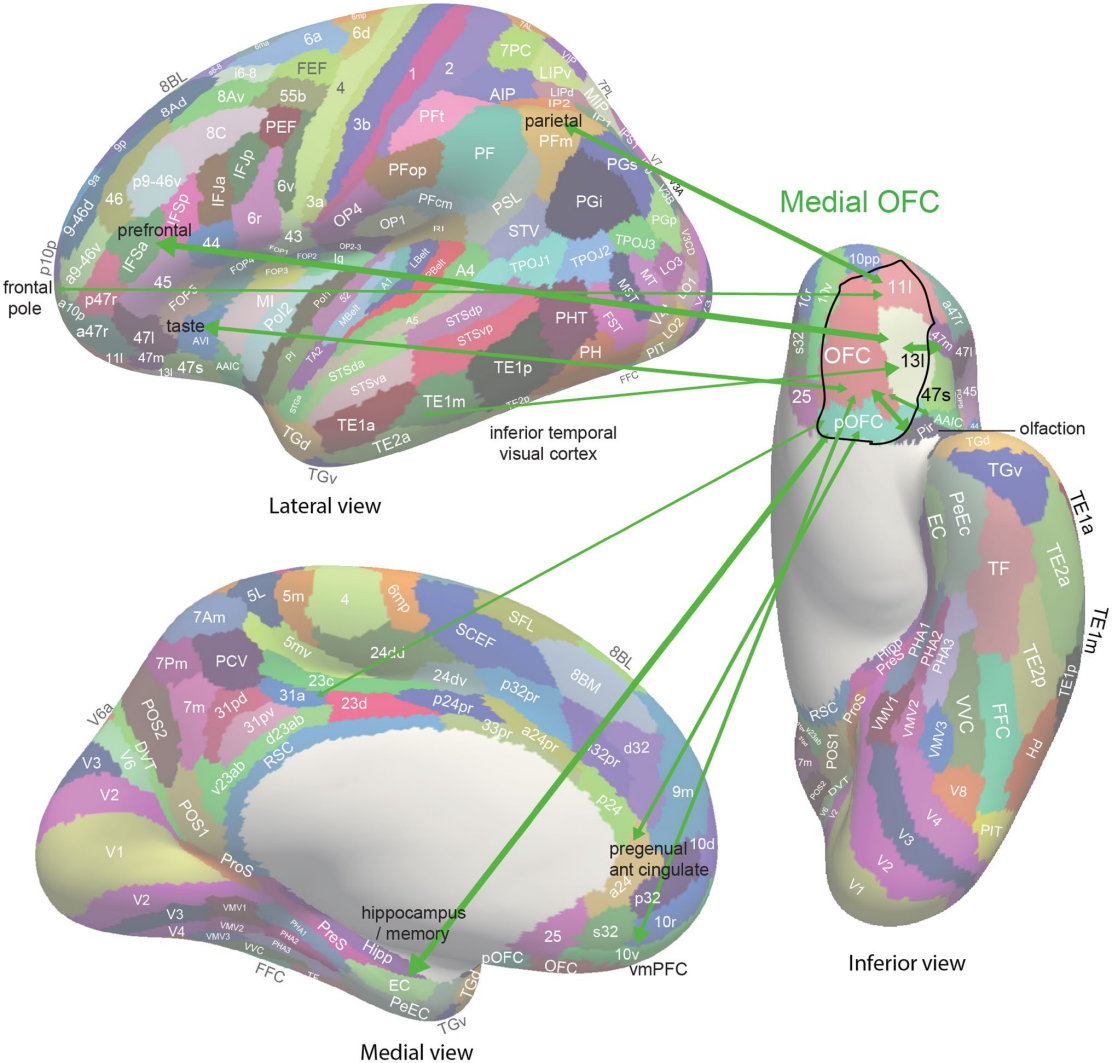
Summary of the effective connectivity of the human medial orbitofrontal cortex. The medial orbitofrontal cortex has taste, olfactory and inferior temporal visual cortex inputs, and connectivity with the hippocampus, pregenual anterior cingulate cortex, ventromedial prefrontal cortex (vmPFC), posterior cingulate cortex (e.g. 31), parietal cortex, inferior prefrontal cortex, and frontal pole. The main regions with which the medial OFC has connectivity are indicated by names with the words in black font. The width of the arrows and the size of the arrow heads in each direction reflects the strength of the effective connectivity. The abbreviations are listed in Rolls et al. (2023d)

**Fig. 6:**
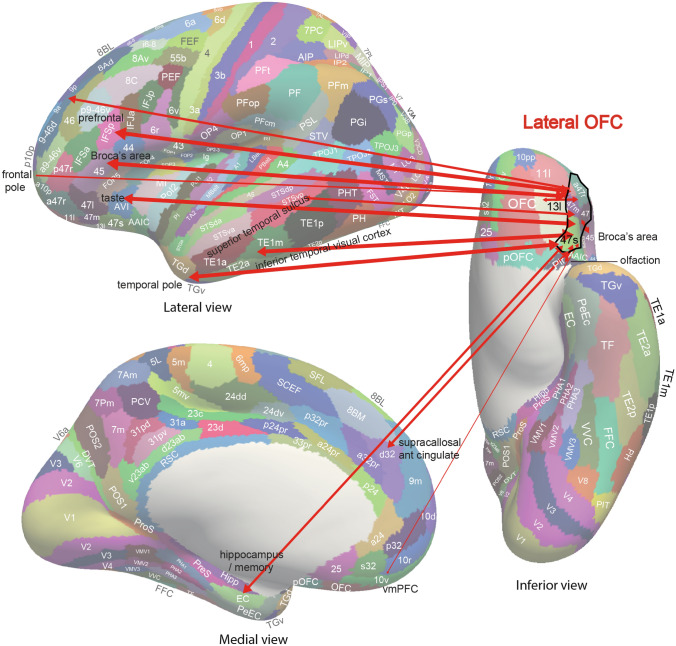
Summary of the effective connectivity of the human lateral orbitofrontal cortex. The lateral orbitofrontal cortex has taste, olfactory and inferior temporal visual cortex inputs, and connectivity with the hippocampus, supracallosal (dorsal) anterior cingulate cortex, inferior and dorsolateral prefrontal cortex, and frontal pole. However, the lateral OFC also has connectivity with language regions (the cortex in the superior temporal sulcus and Broca’s area). The main regions with which the lateral OFC has connectivity are indicated by names with the words in black font. The width of the arrows and the size of the arrow heads in each direction reflects the strength of the effective connectivity. The abbreviations are listed in Rolls et al. (2023d)

The orbitofrontal cortex cytoarchitectonic areas of the human brain are shown in Fig. 4 (left). The medial orbitofrontal cortex includes areas 13 and 11 (Öngür et al. 2003). The lateral orbitofrontal cortex includes area 12 (sometimes in humans termed 12/47) (Öngür et al. 2003). The anterior cingulate cortex includes the parts shown in Fig. 4 (right) of areas 32, 25 (subgenual cingulate), and 24 (see also Figs. 5 and 6). The ventromedial prefrontal cortex includes areas 14 (gyrus rectus), 10m and 10r.

Some of the main connections of the orbitofrontal cortex in primates are shown schematically in Fig. 3 (Carmichael and Price 1994, 1995; Barbas 1995, 2007; Petrides and Pandya 1995; Pandya and Yeterian 1996; Ongür and Price 2000; Price 2006, 2007; Saleem et al. 2008; Mackey and Petrides 2010; Petrides et al. 2012; Saleem et al. 2014; Henssen et al. 2016; Rolls 2017, 2019d, b, Rolls et al. 2020b). The orbitofrontal cortex receives inputs from the ends of every ventral cortical stream that processes the identity of visual, taste, olfactory, somatosensory, and auditory stimuli (Rolls 2019b, 2023d). At the ends of each of these cortical processing streams, the identity of the stimulus is represented independently of its reward value (Rolls 2023d). This is shown by neuronal recordings in primates (Rolls 2019b). For example, the inferior temporal cortex represents objects and faces independently of their reward value as shown by visual discrimination reversals, and by devaluation of reward tests by feeding to satiety (Rolls et al. 1977; Rolls 2012c, 2016c, 2019b). Similarly, the insular primary taste cortex represents what the taste is independently of its reward value (Yaxley et al. 1988; Rolls 2015, 2016d, 2019b, 2023d).

Outputs of the orbitofrontal cortex reach the anterior cingulate cortex, the striatum, the insula, and the inferior frontal gyrus (Rolls 2019a, 2023d; Rolls et al. 2023d), and enable the reward value representations in the orbitofrontal cortex to influence behaviour (Fig. 3, green). The orbitofrontal cortex projects reward value outcome information (e.g. the taste of food) to the anterior cingulate cortex, where it is used to provide the reward outcomes for action–outcome learning (Rushworth et al. 2012; Rolls a, 2019b, 2023d). The orbitofrontal cortex also projects expected reward value information (e.g. the sight of food) to the anterior cingulate cortex where previously learned actions for that goal can be selected. The orbitofrontal cortex projects reward-related information to the ventral striatum (Williams et al. 1993), and this provides a route, in part via the habenula, for reward-related information to reach the dopamine neurons (Rolls 2017), which respond inter alia to positive reward prediction error (Bromberg-Martin et al. 2010; Schultz 2016b). The striatal/basal ganglia route is used for stimulus–response, habit, learning (Everitt and Robbins 2013; Rolls 2014b, 2023d), with dopamine used to provide reward prediction error in reinforcement learning (Schultz 2016c; Cox and Witten 2019). As that system uses dopamine in reinforcement learning of stimulus–response habits, it is much less fast to learn than the orbitofrontal cortex (outcome) with anterior cingulate cortex (action) system for action-outcome goal-based learning, and for emotion (Rolls 2021b). The orbitofrontal cortex projects to the insula as an output pathway and includes a projection to the viscero-autonomic cortex in the antero-ventral insula (Hassanpour et al. 2018; Quadt et al. 2022) that helps to account for why the insula is activated in some tasks in which the orbitofrontal cortex is involved (Rolls 2016d, 2019b, 2023d). This antero-ventral part of the insula (Quadt et al. 2022) is just ventral to the primary taste cortex, and has very strong connections in primates to (and probably from) the orbitofrontal cortex (Baylis et al. 1995). The orbitofrontal cortex also projects to the inferior frontal gyrus, a region that on the right is implicated in stopping behaviour (Aron et al. 2014).

**Fig. 7:**
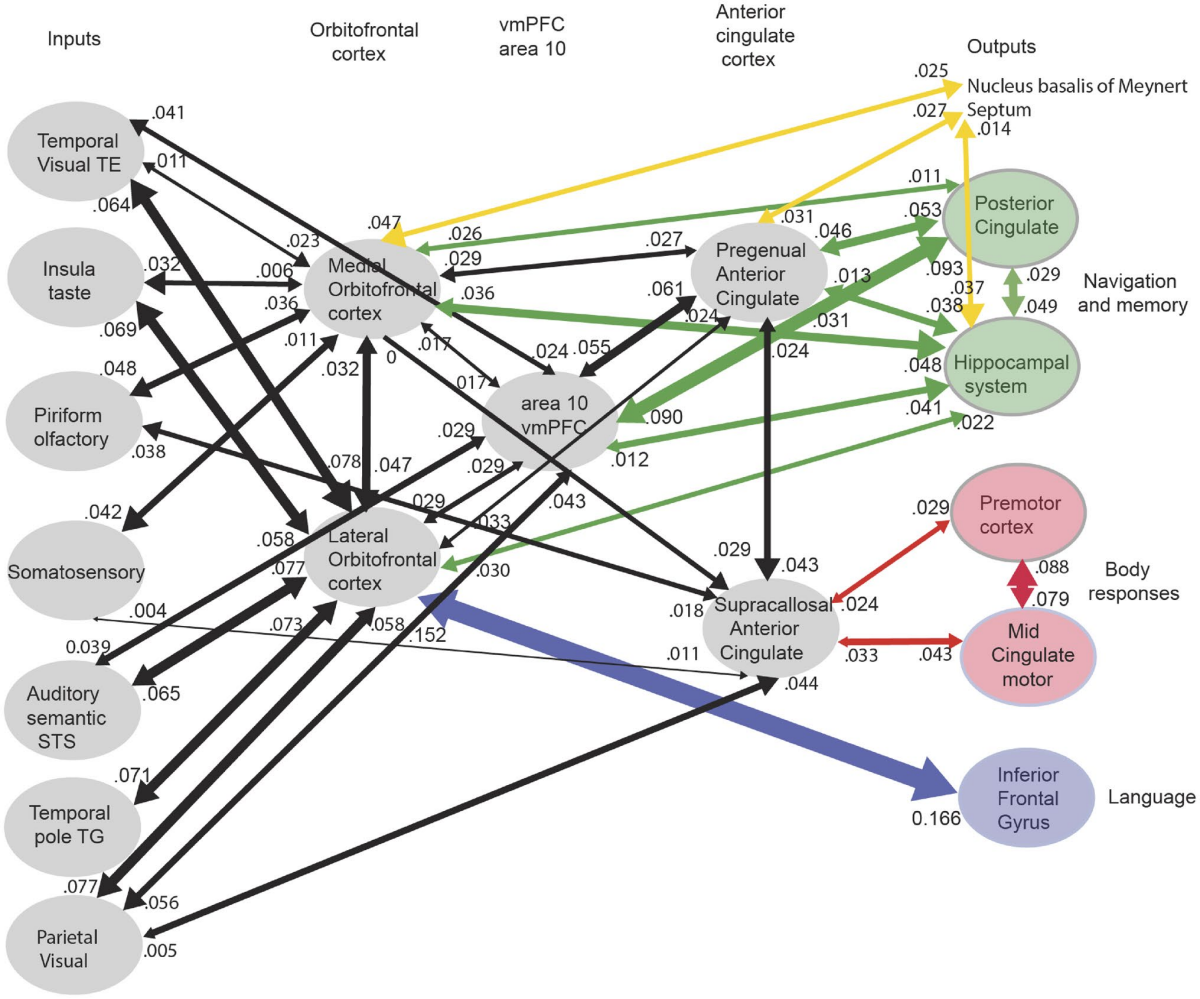
Effective connectivity of the human orbitofrontal cortex, vmPFC, and anterior cingulate cortex shown in the middle, with inputs on the left and outputs on the right. The effective connectivity was measured in 171 participants imaged at 7 T by the Human Connectome Project, and was measured between the 360 cortical regions in the HCP-multimodal parcellation atlas (Glasser et al. 2016a), with subcortical regions using the HCPex atlas (Huang et al. 2022). The effective connectivity measures the effect in each direction between every pair of cortical regions, uses time delays to assess the directionality using a Hopf computational model which integrates the dynamics of Stuart–Landau oscillators in each cortical region, has a maximal value of 0.2, and is described in detail elsewhere (Rolls et al. 2022a; b, 2023d). The width of the arrows is proportional to the effective connectivity in the highest direction, and the size of the arrowheads reflects the strength of the effective connectivity in each direction. The effective connectivities shown by the numbers are for the strongest link where more than one link between regions applies for a group of brain regions. Effective connectivities with hippocampal memory system regions are shown in green; with premotor/mid-cingulate regions in red; with the inferior prefrontal language system in blue; and in yellow to the basal forebrain nuclei of Meynert which contains cholinergic neurons that project to the neocortex and to the septal nuclei which contain cholinergic neurons that project to the hippocampus. The Somatosensory regions include 5 and parietal PF and PFop, which also connect to the pregenual anterior cingulate but are not shown for clarity; the Parietal regions include visual parietal regions 7, PGi and PFm. (From Rolls et al (2023d))

New evidence on the connectivity of the orbitofrontal cortex in humans is shown in Figs. 5, 6, 7, based on measurements of effective connectivity between 360 cortical regions and 24 subcortical regions measured in 171 humans from the Human Connectome Project, and complemented with functional connectivity and diffusion tractography (Rolls et al. 2023d). Effective connectivity measures ‘causal’ effects (in that they take into account time delays) in each direction between every pair of brain regions. (Although time delays are a signature of causality, further evidence is needed to prove causality, such as interventions (Rolls 2021f, e).) The effective connectivities of the orbitofrontal cortex with other brain regions are summarised in Figs. 5, 6, 7 (Rolls et al. 2023a; d). The medial and lateral orbitofrontal cortex between them (and they have effective connectivity with each other) receive taste, somatosensory, olfactory, visual, and auditory inputs that are needed to build the reward and punishment value representations that are found in these regions but much less in the preceding cortical areas that provide these inputs (Rolls 2019d, 2019b, 2021a, 2023d). Taste and somatosensory inputs provide information about primary reinforcers or outcome value, and the orbitofrontal cortex contains visual and olfactory neurons that can learn and reverse in one trial the associations with primary reinforcers and so represent expected value (Thorpe et al. 1983). This is consistent with the schematic diagram in Fig. 3.

In more detail (Fig. 5) (Rolls et al. 2023a; d), parts of the medial orbitofrontal cortex (11l, 13l, OFC and pOFC, which are interconnected) have effective connectivity with the taste/olfactory/visceral anterior agranular insular complex (AAIC); the piriform (olfactory) cortex; the entorhinal cortex (EC); the inferior temporal visual cortex (TE1p, TE2a, TE2p); superior medial parietal 7Pm; inferior parietal PF which is somatosensory (Rolls et al. 2023e, f); with parts of the posterior cingulate cortex (31pv, 7m, d23ab) related to memory (Rolls et al. 2023i); with the pregenual anterior cingulate cortex (s32, a24, p24, p32, d32) and much less with the supracallosal anterior cingulate cortex (only 33pr); with ventromedial prefrontal 10r, 10d and 9m; with the frontal pole (10pp, p10p, a10p); with lateral orbitofrontal cortex (47m, 47s, a47r); and dorsolateral prefrontal cortex (46 and a9-46v) (Rolls et al. 2023e). Medial orbitofrontal cortex regions also have effective connectivity directed towards the caudate nucleus and nucleus accumbens (Rolls et al. 2023d).

Also with some detail, the lateral orbitofrontal cortex areas a47r, p47r and 47m share generally similar effective connectivities (Fig. 6) (Rolls et al. 2023a; d) from the visual inferior temporal cortex (TE areas); from parts of the parietal cortex [PFm which receives visual and auditory object-level information and IP2 which is visuomotor (Rolls et al. 2023f)]; from the medial orbitofrontal cortex (11l, 13l, pOFC); from the inferior frontal gyrus regions including IFJ, IFS and BA45; from the dorsolateral prefrontal cortex (8Av, 8BL, a9-46v and p9-46v) implicated in short-term memory (Rolls 2023d; Rolls et al. 2023e); and from the frontal pole (a10p, p10p, 10pp) (Rolls et al. 2023a; d). 47m (which is relatively medial in this group) also has effective connectivity with the hippocampal system (Hipp, EC, perirhinal, and TF), and with ventromedial prefrontal region 10r; and with the frontal pole [10d, and 9m (Rolls et al. 2023c)]. The diffusion tractography provides in addition evidence for connections of these parts of the lateral orbitofrontal cortex with the anterior ventral insular region (AVI) and the frontal opercular areas FOP4 and FOP5 which include the insular primary taste cortex (Rolls 2015, 2016d; Rolls et al. 2023a, d); with the anterior agranular insular complex (AAIC) which may be visceral (Rolls 2016d) and also has taste-olfactory convergence (De Araujo et al. 2003a); with the middle insular region (MI) which is somatosensory (Rolls et al. 2023e); and with the piriform (olfactory) cortex.

The human orbitofrontal cortex has connectivity to the hippocampal memory/navigation system that is both direct, and via the ventromedial area 10 regions (10r, 10d, 10v and 9m), pregenual anterior cingulate cortex, and the memory-related parts of the posterior cingulate cortex (Fig. 7). It is proposed that this connectivity provides a key input about reward/punishment value for the hippocampal episodic memory system, adding to the ‘what’, ‘where’, and ‘when’ information that are also key components of episodic memory (Rolls 2022b; Rolls et al. 2023d). Damage to the vmPFC/anterior cingulate cortex system is likely to contribute to episodic memory impairments by impairing a key component of episodic memory, the reward/punishment/emotional value component (Rolls 2022b; Rolls et al. 2023d). Moreover, the medial orbitofrontal cortex connects to the nucleus basalis of Meynert and the pregenual cingulate to the septum, and damage to these cortical regions may contribute to memory impairments by disrupting cholinergic influences on the neocortex and hippocampus (Rolls 2022b; Rolls et al. 2023d). Navigation is generally towards goals, usually rewards, and it is proposed that this connectivity provides the goals for navigation to the hippocampal system to enable the hippocampus to be involved in navigation towards goals (Rolls 2022b, 2023c; Rolls et al. 2023d).

Two regions of the lateral orbitofrontal cortex, 47l and 47s, are especially connected with language systems in the temporal pole, cortex in the superior temporal sulcus (STS), and inferior frontal gyrus including Broca’s area 45 and 44 (Rolls et al. 2022a). This provides a route for subjective reports to be made about the pleasantness or unpleasantness of stimuli and events (Rolls 2023d).

In the context that the anterior cingulate cortex is implicated in learning associations between actions and the rewards or punishers associated with the actions (Noonan et al. 2011; Rushworth et al. 2012; Rolls 2019a, 2023d), the part of the anterior cingulate cortex that is most likely to be involved in action–outcome learning is the supracallosal (or dorsal) anterior cingulate cortex. That part has effective connectivity with somato-motor areas involved in actions, but which as shown in Fig. 7 receives inputs from the medial orbitofrontal cortex and pregenual anterior cingulate cortex that it is proposed provide the reward/punishment ‘outcome’ signals necessary for action–outcome learning (Rolls 2023d; Rolls et al. 2023d).

#### The human medial orbitofrontal cortex represents reward value

**Fig. 8:**
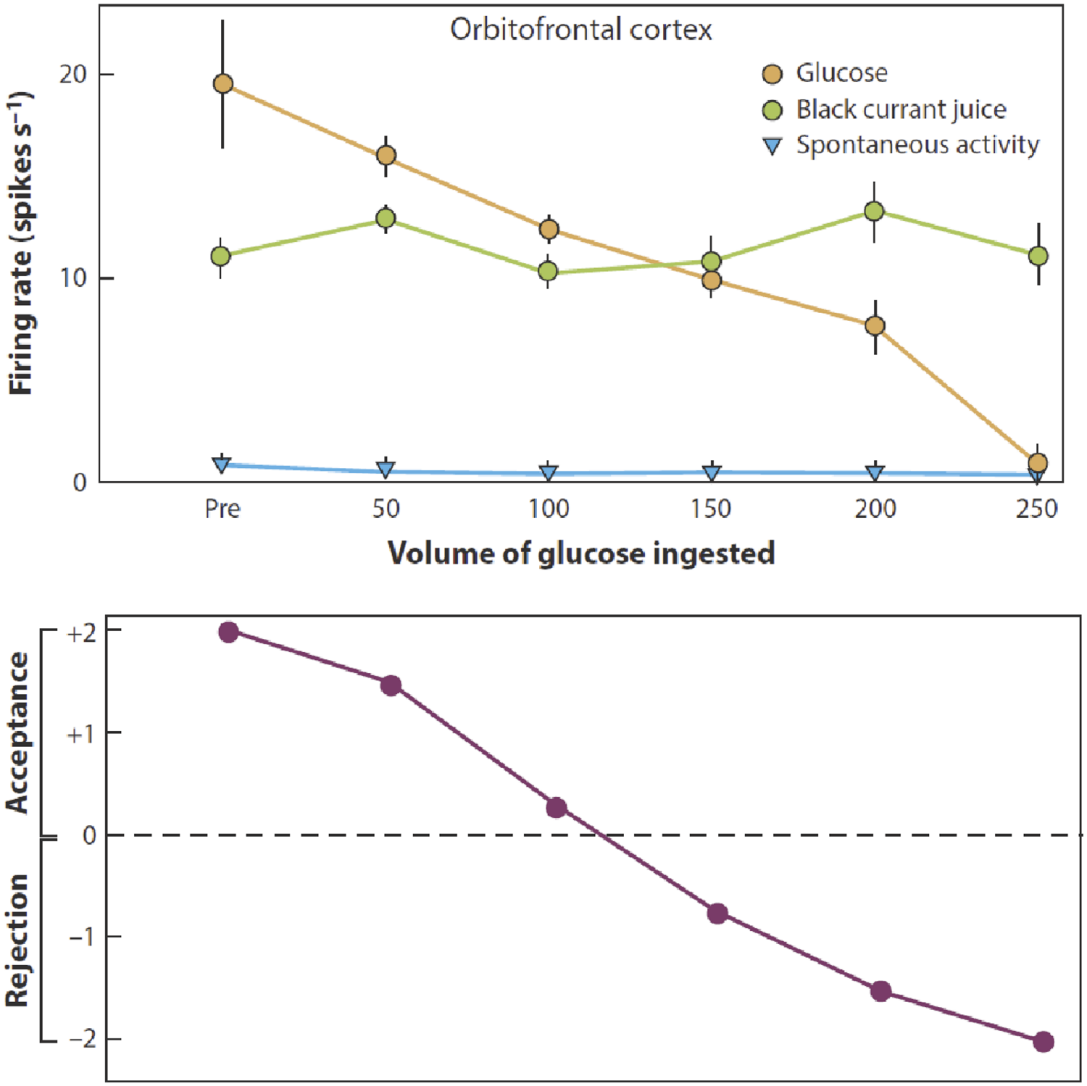
The effect of feeding to satiety with glucose solution on the responses (firing rate ± s.e.m.) of a neuron in the orbitofrontal (secondary taste) cortex to the taste of glucose (open circles) and of blackcurrant juice (BJ). The spontaneous firing rate is also indicated (SA). Below the neuronal response data, the behavioural measure of the acceptance or rejection of the solution on a scale from + 2 (strong acceptance) to − 2 (strong rejection) is shown. The solution used to feed to satiety was 20% glucose. The monkey was fed 50 ml of the solution at each stage of the experiment as indicated along the abscissa, until he was satiated as shown by whether he accepted or rejected the solution. Pre is the firing rate of the neuron before the satiety experiment started. (Reproduced from Rolls et al. 1989, Copyright 1989 Society for Neuroscience.)

The primate including human orbitofrontal cortex is the first stage of cortical processing that represents reward value (red in Fig. 3) (Rolls 2019b, d, 2021b). For example, in devaluation experiments, taste, olfactory, visual, and oral texture neurons in the macaque orbitofrontal respond to food when hunger is present, and not after feeding to satiety when the food is no longer rewarding (Rolls et al. 1989; Critchley and Rolls 1996). An example of a devaluation experiment is shown in Fig. 8, which shows that as the value of the taste of glucose is reduced by feeding glucose to satiety, a typical orbitofrontal cortex neuron responding to the taste of food when it is rewarding at the start of the experiment gradually reduces its response to zero as the reward value reaches zero because glucose had been consumed. In fact, the experiment shows more than this, for the effect is relatively specific to the food eaten to satiety: there was little reduction of the firing rate to the flavour of fruit (black currant) juice after glucose had been fed to satiety. Correspondingly, the black currant juice was still rewarding after feeding to satiety with glucose (Fig. 8). Thus satiety is somewhat specific to the reward that has been received, and this is termed sensory-specific satiety. In fact, sensory-specific satiety was discovered when we were recording from lateral hypothalamic neurons responding to the taste and/or sight of food (Rolls et al. 1986). We traced back the computation to the orbitofrontal cortex, in which neurons show sensory-specific satiety to a primary reinforcer, the taste of food (Rolls et al. 1989), and to a secondary reinforcer, the sight and smell of food (Critchley and Rolls 1996). Devaluation effects are not found in the stages that provide taste information to the orbitofrontal cortex, the insular/opercular primary taste cortex (Rolls et al. 1988; Yaxley et al. 1988), nor in the brain region that provides visual inputs to the orbitofrontal cortex, the inferior temporal visual cortex (Rolls et al. 1977). This is some of the evidence on which Fig. 3 is based. The devaluation procedure has been adopted by others (Rudebeck et al. 2017; Murray and Rudebeck 2018; Murray and Fellows 2022).

This discovery of sensory-specific satiety has enormous implications, for it is proposed to apply to all rewards and to no punishers (Rolls 2014b, 2018, 2022a), and has the evolutionary adaptive value that behaviour switches from one reward to another. This ensures for example that a wide range of nutrients will be ingested [as we showed in experiments we performed with Oxford undergraduates after the neurophysiological discovery (Rolls et al. 1981a, b, c)] (though obesity is a resulting risk if a wide range of nutrients becomes easily available for humans) (Rolls 2016a); and more generally tends to promote reproductive success for the genes, in that a wide range of possible rewards will be explored (Rolls 2014b, 2018) (see "Some implications and extensions of the understanding of emotion, motivation, and their brain mechanisms"). Sensory-specific satiety is thus a key factor in emotion.

Further evidence that reward value is represented in the orbitofrontal cortex is that in visual discrimination reversal experiments, neurons in the macaque orbitofrontal cortex reverse the visual stimulus to which they respond in as little as one trial when the reward vs punishment taste received as an outcome for the choice reverses (Thorpe et al. 1983; Rolls et al. 1996). This is rule-based reversal, in that after a previously rewarded visual stimulus is no longer rewarded, the macaques choose the other stimulus on the very next trial, even though its previous reward association was with punishment, as illustrated in Fig. 10c which also illustrates a non-reward neuron active at the time of the reversal (Thorpe et al. 1983). (Non-reward refers here to not obtaining an expected reward.) This capability requires a rule to be held in memory and reversed by non-reward (Deco and Rolls 2005c; Rolls and Deco 2016) (which is described as model-based), is very appropriate for primates including humans who in social situations may benefit from being very responsive to non-reward vs reward signals, and may not occur in rodents (Rolls 2019b, 2021b; Hervig et al. 2020). The macaque orbitofrontal cortex also contains visual neurons that reflect face expression and face identity (both necessary to decode the reward/punishment value of an individual) (Thorpe et al. 1983; Rolls et al. 2006), and also social categories such as young faces (Barat et al. 2018). Information about face expression and movements important in social communication probably reaches the orbitofrontal cortex from neurons we discovered in the cortex in the macaque superior temporal sulcus that respond to these stimuli (Hasselmo et al. 1989a, b), in what is a region now accepted as important for decoding visual stimuli relevant to social behaviour (Pitcher et al. 2019; Pitcher and Ungerleider 2021). Economic value is represented in the orbitofrontal cortex, in that for example single neurons reflect the trade-off between the quality of a reward and the amount that is available (Padoa-Schioppa and Cai 2011; Padoa-Schioppa and Conen 2017). These investigations show that some orbitofrontal cortex neurons respond to outcome value (e.g. the taste of food), and others to expected value (of future rewards). The expected value neurons are not positive reward prediction error neurons, for they keep responding to the expected reward even when there is no prediction error (Rolls 2021b). Consistent with this, lesions of the macaque medial orbitofrontal cortex areas 13 and 11 make the animals less sensitive to reward value, as tested in devaluation experiments in which the animal is fed to satiety (Rudebeck et al. 2017). Neurotoxic lesions of the macaque orbitofrontal cortex produce effects that are difficult to interpret (Murray and Rudebeck 2018; Sallet et al. 2020), perhaps because these lesions have not always been based on knowledge of where neurons and activations related to reversal learning are found, and the difficulty of disabling all such orbitofrontal cortex neurons. Further, the tasks used in these studies are sometimes complicated, whereas a prototypical task is deterministic one-trial Go-NoGo rule-based visual discrimination reversal between the a visual stimulus and taste (Thorpe et al. 1983; Rolls et al. 1996), or in humans between a visual stimulus and winning or losing points or money (Rolls et al. 2020c). Rodents appear not to be able to perform this one-trial rule-based visual-reward reversal task (Hervig et al. 2020).

**Fig. 9:**
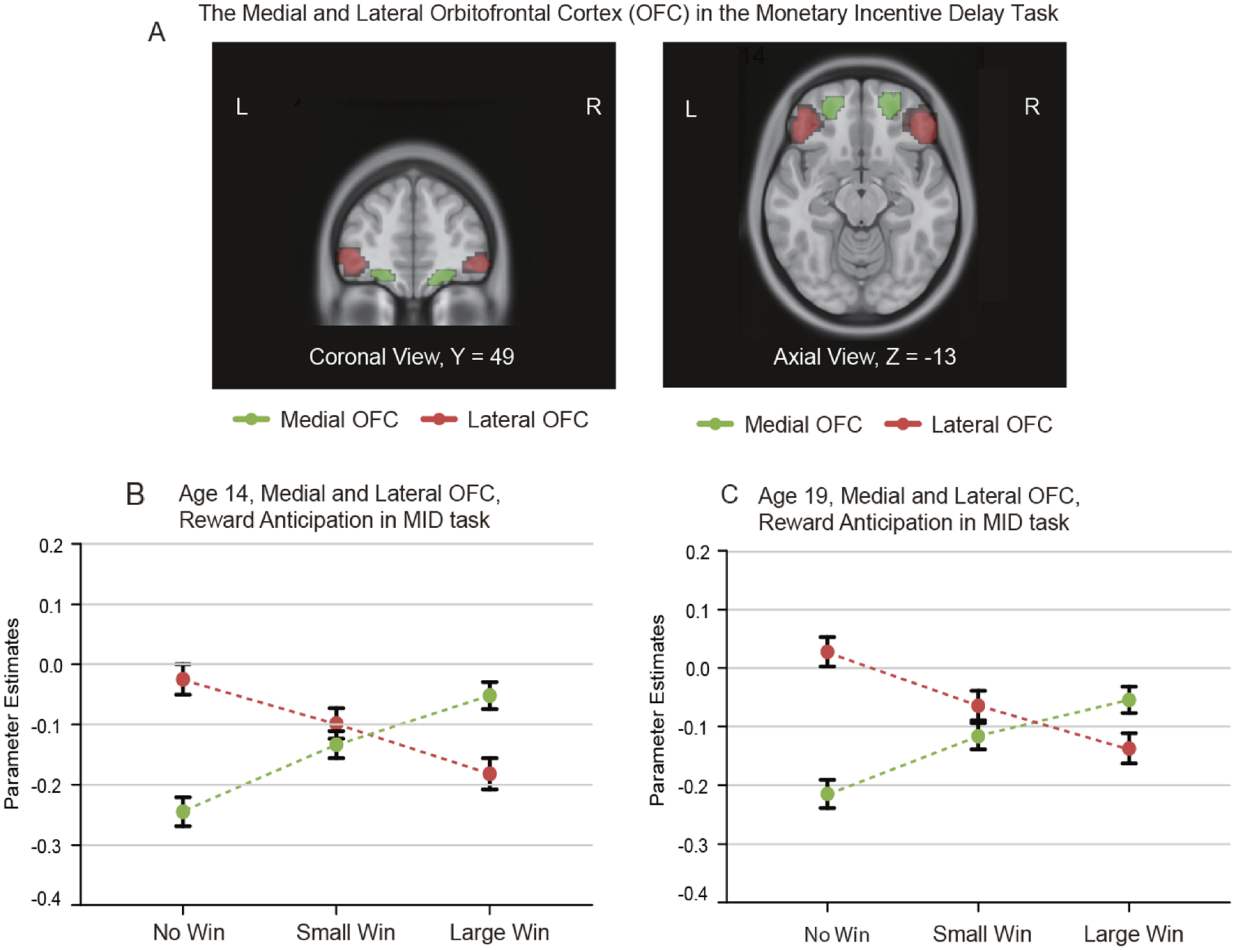
The lateral orbitofrontal cortex is activated by not winning, and the medial orbitofrontal cortex by winning, in the monetary incentive delay task. The lateral orbitofrontal cortex region in which activations increased towards no reward (No Win) in the monetary incentive delay task are shown in red in 1140 participants at age 19 and in 1877 overlapping participants at age 14. The conditions were Large win (10 points) to Small Win (2 points) to No Win (0 points) (at 19; sweets were used at 14). The medial orbitofrontal cortex region in which activations increased with increasing reward from No Win to Small Win to High Win) is shown in green. The parameter estimates are shown from the activations for the participants (mean ± sem) with the lateral orbitofrontal in red and medial orbitofrontal cortex in green. The interaction term showing the sensitivity of the medial orbitofrontal cortex to reward and the lateral orbitofrontal cortex to non-reward was significant at *p* = 10^–50^ at age 19 and *p* < 10^–72^ at age 14. In a subgroup with depressive symptoms as shown by the Adolescent Depression Rating Scale, it was further found that there was a greater activation to the No Win condition in the lateral orbitofrontal cortex; and the medial orbitofrontal cortex was less sensitive to the differences in reward value. (Modified from Xie et al. 2021.)

Neuroimaging experiments in humans produce consistent evidence (De Araujo et al. 2003a; Kringelbach et al. 2003; Kringelbach and Rolls 2003; Grabenhorst and Rolls 2008; Grabenhorst et al. 2008a), and allow the types of reward to be extended to include monetary reward (O'Doherty et al. 2001; Xie et al. 2021), face expressions (Kringelbach and Rolls 2003), and face beauty (O'Doherty et al. 2003). Further, in humans activations of the medial orbitofrontal cortex are linearly related to the subjective (conscious) pleasantness of stimuli (Grabenhorst and Rolls 2011; Rolls 2019b). These reward-related effects are found for odors (Rolls et al. 2003b), flavor (De Araujo et al. 2003a; Kringelbach et al. 2003), pleasant touch (Rolls et al. 2003c; McCabe et al. 2008), monetary reward (O'Doherty et al. 2001; Xie et al. 2021), and amphetamine (Völlm et al. 2004). A recent study with 1140 participants emphasizes these points, by showing that the medial orbitofrontal cortex is activated by reward [such as winning money or candies), and that the lateral orbitofrontal cortex is activated by not winning (Fig. 9 (Xie et al. 2021)].

Further, humans with orbitofrontal cortex lesions may also be less sensitive to reward, as shown by their reduced subjective emotional feelings (Hornak et al. 2003), and their difficulty in identifying face and voice emotion-related expressions, which are important for emotional and social behaviour (Hornak et al. 1996, 2003).

#### The human lateral orbitofrontal cortex represents punishers and non-reward, and is involved in changing emotional behaviour

**Fig. 10:**
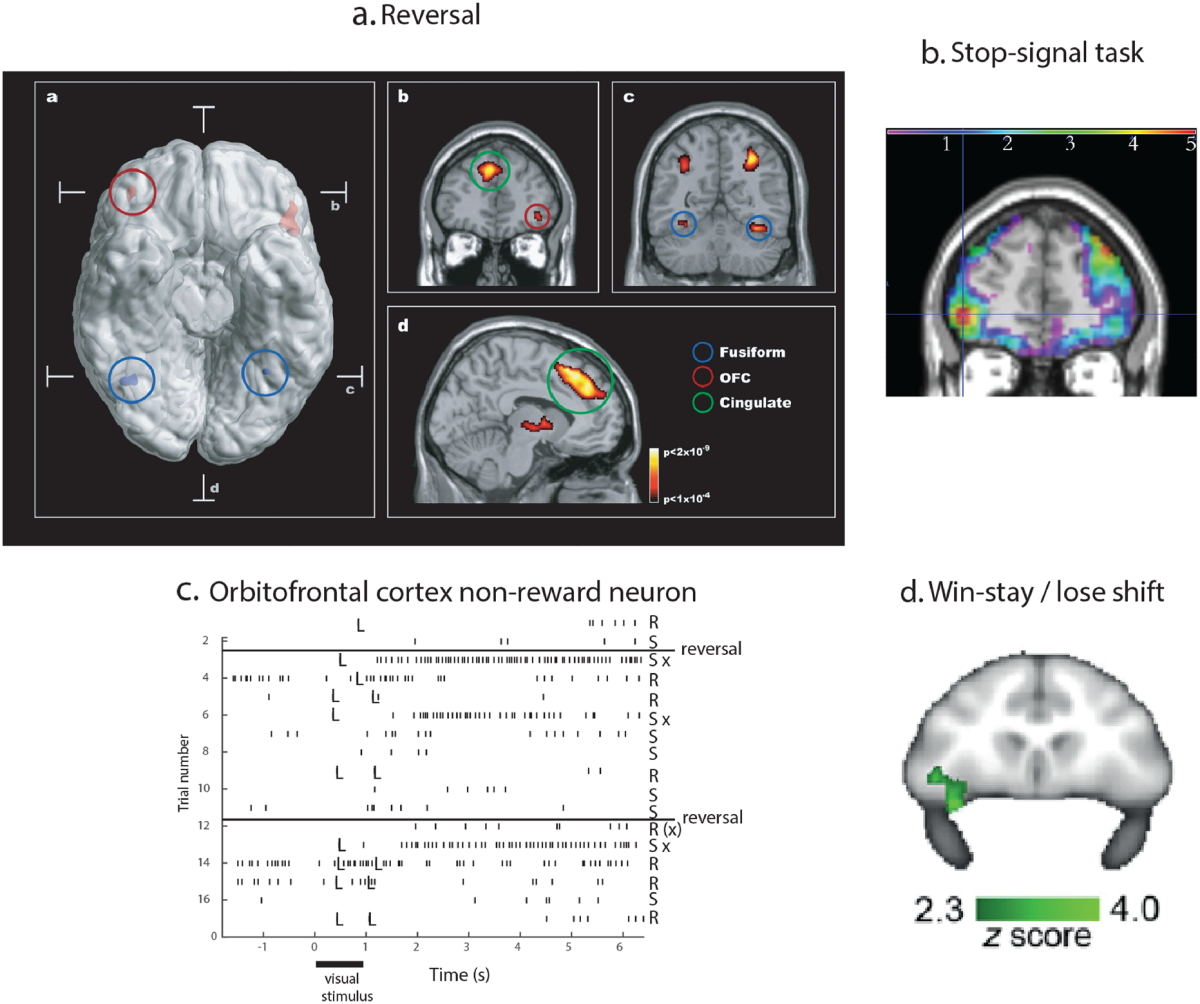
**a** Evidence that the human lateral orbitofrontal cortex is activated by non-reward. Activation of the lateral orbitofrontal cortex in a visual discrimination reversal task on reversal trials, when a face was selected but the expected reward was not obtained, indicating that the subject should select the other face in future to obtain the reward. **a** A ventral view of the human brain with indication of the location of the two coronal slices (**b**, **c**) and the transverse slice (**d**). The activations with the red circle in the lateral orbitofrontal cortex (OFC, peaks at [42 42 − 8] and [− 46 30 − 8]) show the activation on reversal trials compared to the non-reversal trials. For comparison, the activations with the blue circle show the fusiform face area produced just by face expressions, not by reversal, which are also indicated in the coronal slice in **c**. **b** A coronal slice showing the activation in the right orbitofrontal cortex on reversal trials. Activation is also shown in the supracallosal anterior cingulate region (Cingulate, green circle) that is also known to be activated by many punishing, unpleasant, stimuli (see Grabenhorst and Rolls (2011)). (From NeuroImage 20 (2), Morten L. Kringelbach and Edmund T. Rolls, Neural correlates of rapid reversal learning in a simple model of human social interaction, pp. 1371–83, Copyright, 2003, with permission from Elsevier.). **b** Activations in the human lateral orbitofrontal cortex are related to a signal to change behaviour in the stop-signal task. In the task, a left or right arrow on a screen indicates which button to touch. However on some trials, an up-arrow then appears, and the participant must change the behaviour, and stop the response. There is a larger response on trials on which the participant successfully changes the behaviour and stops the response, as shown by the contrast stop-success–stop-failure, in the ventrolateral prefrontal cortex in a region including the lateral orbitofrontal cortex, with peak at [− 42 50 − 2] indicated by the cross-hairs, measured in 1709 participants. There were corresponding effects in the right lateral orbitofrontal cortex [42 52 − 4]. Some activation in the dorsolateral prefrontal cortex in an area implicated in attention is also shown. (After Deng, Rolls et al. 2016). **c** Non-reward error-related neurons maintain their firing after non-reward is obtained. Responses of an orbitofrontal cortex neuron that responded only when the macaque licked to a visual stimulus during reversal, expecting to obtain fruit juice reward, but actually obtained the taste of aversive saline because it was the first trial of reversal (trials 3, 6, and 13). Each vertical line represents an action potential; each L indicates a lick response in the Go-NoGo visual discrimination task. The visual stimulus was shown at time 0 for 1 s. The neuron did not respond on most reward (R) or saline (S) trials, but did respond on the trials marked S x, which were the first or second trials after a reversal of the visual discrimination on which the monkey licked to obtain reward, but actually obtained saline because the task had been reversed. The two times at which the reward contingencies were reversed are indicated. After responding to non-reward, when the expected reward was not obtained, the neuron fired for many seconds, and was sometimes still firing at the start of the next trial. It is notable that after an expected reward was not obtained due to a reversal contingency being applied, on the very next trial the macaque selected the previously non-rewarded stimulus. This shows that rapid reversal can be performed by a non-associative process, and must be rule-based. (After Thorpe et al. 1983.) **d** Bold signal in the macaque lateral orbitofrontal related to win-stay/lose-shift performance, that is, to reward reversal performance. (After Chau et al 2015)

The macaque orbitofrontal cortex has neurons that respond when an expected reward is not received (Thorpe et al. 1983), and these have been termed *non-reward neurons* (Rolls 2014b, 2019b, d, 2021b) (see example in Fig. 10c). They can be described as negative reward prediction error neurons, in that they respond when a reward outcome is less than was expected (Rolls 2019b). These neurons do not respond to expected punishers [e.g. the discriminative stimulus for saline in Fig. 10c (Thorpe et al. 1983)], but other neurons do respond to expected punishers (Rolls et al. 1996), showing that non-reward and punishment are represented by different neurons in the orbitofrontal cortex. The finding of non-reward neurons is robust, in that 18/494 (3.6%) of the neurons in the original study responded to non-reward (Thorpe et al. 1983), consistent results were found in different tasks in a complementary study (10/140 non-reward neurons in the orbitofrontal cortex or 7.1%) (Rosenkilde et al. 1981), and an fMRI study has shown that the macaque lateral orbitofrontal cortex is activated when an expected reward is not obtained during reversal (Chau et al. 2015) (Fig. 10d). The hypothesis is that the non-reward responsiveness of these neurons is computed in the orbitofrontal cortex, because this is the first brain region in primates at which expected value and outcome value are represented, as summarized in Fig. 3 and with the evidence set out fully by Rolls (2019b, 2021b, 2023d), and these two signals are those required to compute non-reward, that is, that reward outcome is less than the expected value.

Corresponding to this, the human lateral orbitofrontal cortex is activated when a reward is not obtained in a visual discrimination reversal task (Kringelbach and Rolls 2003) (Fig. 10a), and when money is not received in a monetary reward task (O'Doherty et al. 2001; Xie et al. 2021), and in a one-trial reward reversal task (Rolls et al. 2020c). Further, the human lateral orbitofrontal cortex is also activated by punishing, subjectively unpleasant, stimuli (Grabenhorst and Rolls 2011; Rolls 2019b, d, 2021b). Examples include unpleasant odors (Rolls et al. 2003b), pain (Rolls et al. 2003c), losing money (O'Doherty et al. 2001), and receiving an angry face expression indicating that behaviour should change in a reversal (Kringelbach and Rolls 2003). The human right lateral orbitofrontal cortex/inferior frontal gyrus is also activated when behavioural correction is required in the stop-signal task (Fig. 10b) (Aron et al. 2014; Deng et al. 2017). These discoveries show that one way in which the orbitofrontal cortex is involved in decision-making and emotion is by representing rewards, punishers, and errors made during decision-making. This is supported by the problems that orbitofrontal cortex damage produces in decision-making, which including failing to respond correctly to non-reward, as described next.

Consistent with this neurophysiological and neuroimaging evidence, lesions of the orbitofrontal cortex can impair reward reversal learning during decision-making in humans (Rolls et al. 1994; Hornak et al. 2004; Fellows 2011), who continue responding to the previously rewarded, now non-rewarded, stimulus. The change in contingency between the stimulus and reward vs non-reward is not processed correctly. In macaques, damage to the lateral orbitofrontal cortex impairs reversal and extinction (Butter 1969; Iversen and Mishkin 1970), and damage of the lateral orbitofrontal cortex area 12 extending around the inferior convexity impaired the ability to make choices based on whether reward vs non-reward had been received (Rudebeck et al. 2017; Murray and Rudebeck 2018). Further evidence that the lateral orbitofrontal cortex is involved in learning contingencies between stimuli and reward vs non-reward is that in humans, lateral orbitofrontal cortex damage impaired this type of 'credit assignment' (Noonan et al. 2017). This type of flexibility of behaviour is important in primate including human social interactions, and indeed many of the effects of damage to the human orbitofrontal cortex, including the difficulty in responding appropriately to the changed circumstances of the patient, and the changed personality including impulsivity, can be related to these impairments in responding to non-reward and punishers (Rolls et al. 1994; Berlin and Rolls 2004; Berlin et al. 2004; Hornak et al. 2004; Rolls 2018, 2019b, d, 2021c, b; Rolls et al. 2020b).

#### The ventromedial prefrontal cortex and reward-related decision-making

The ventromedial prefrontal cortex (vmPFC, which can be taken to include the gyrus rectus area 14 and parts of 10m and 10r, Fig. 4) receives inputs from the orbitofrontal cortex, and has distinct connectivity (with strong functional connectivity with the superior medial prefrontal cortex, cingulate cortex, and angular gyrus Du et al. 2020; Rolls et al. 2023d)). The vmPFC has long been implicated in reward-related decision-making (Bechara et al. 1997, 2005; Glascher et al. 2012), this region is activated during decision-making contrasted with reward valuation (Grabenhorst et al. 2008b; Rolls and Grabenhorst 2008), and it has the signature of a decision-making region of increasing its activation in proportion to the difference in the decision variables, which correlates with decision confidence (Rolls et al. 2010a, b; Rolls 2019b, 2021b). Consistently, in macaques single neurons in the ventromedial prefrontal cortex rapidly come to signal the value of the chosen offer, suggesting that this vmPFC system serves to produce a choice (Strait et al. 2014), also consistent with the attractor model of decision-making (Rolls and Deco 2010; Rolls et al. 2010a, b; Rolls 2014b, 2016c, 2021b).

**Fig. 11:**
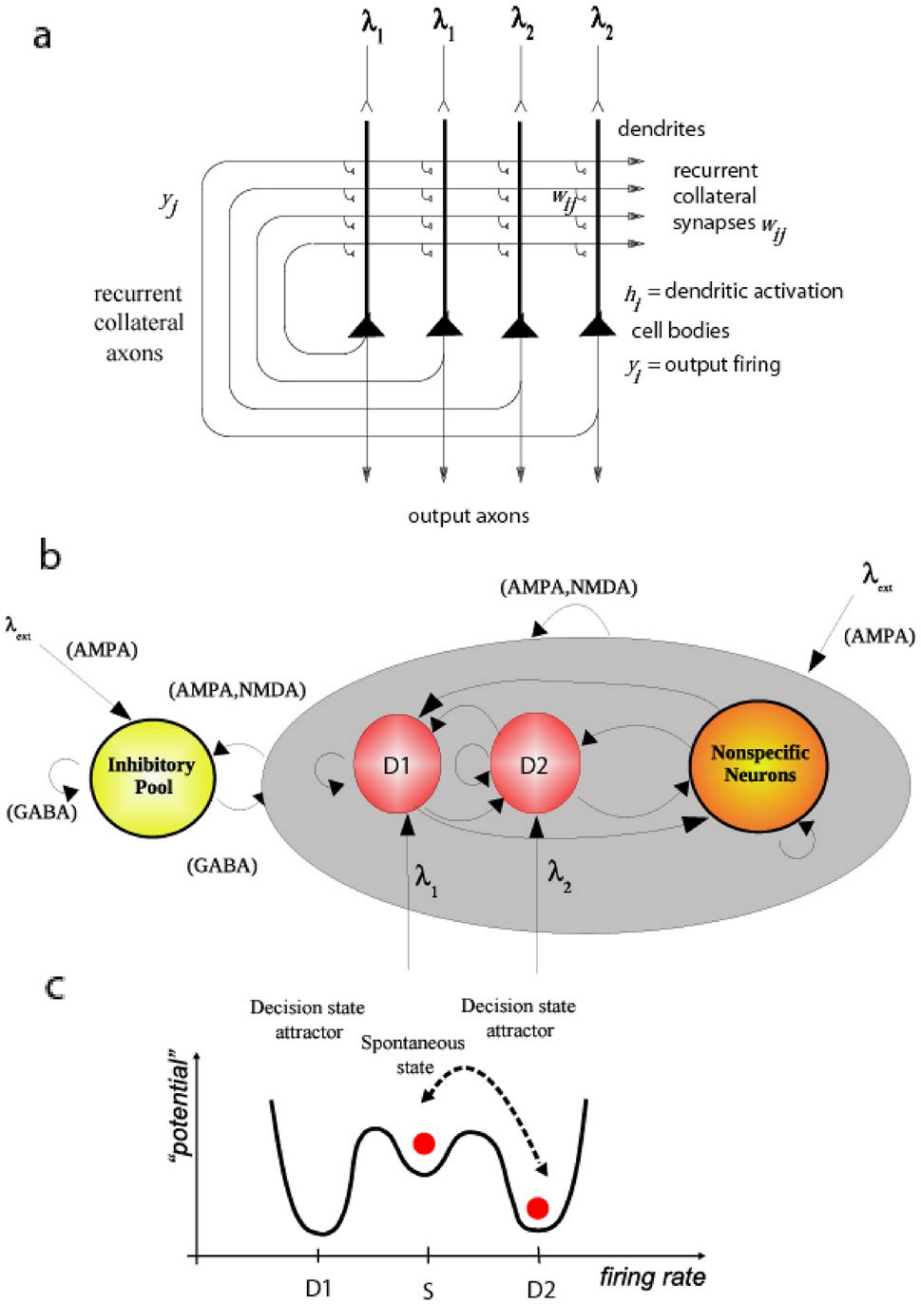
**a** Attractor or autoassociation single network architecture for decision-making. The evidence for decision 1 is applied via the *λ*_1_ inputs, and for decision 2 via the *λ*_2_ inputs. The synaptic weights have been associatively modified during training in the presence of *λ*_1_ and at a different time of *λ*_2_. When *λ*_1_ and *λ*_2_ are applied, each attractor competes through the inhibitory interneurons (not shown), until one wins the competition, and the network falls into one of the high firing rate attractors that represents the decision. The noise in the network caused by the random spiking of the neurons means that on some trials, for given inputs, the neurons in the decision 1 (D1) attractor are more likely to win, and on other trials the neurons in the decision 2 (D2) attractor are more likely to win. This makes the decision-making probabilistic, for, as shown in **c**, the noise influences when the system will jump out of the spontaneous firing stable (low energy) state S, and whether it jumps into the high firing state for decision 1 (D1) or decision 2 (D2). **b** The architecture of the integrate-and-fire network used to model decision-making (see text). **c** A multistable “effective energy landscape” for decision-making with stable states shown as low “potential” basins. Even when the inputs are being applied to the network, the spontaneous firing rate state is stable, and noise provokes transitions into the high firing rate decision attractor state D1 or D2 (see Rolls and Deco 2010; Rolls et al. 2010a, b; Rolls 2021b)

The attractor model of decision-making is a neuronal network with associatively modifiable recurrent collateral synapses between the neurons of the type prototypical of the cerebral cortex (Wang 2002; Rolls and Deco 2010; Rolls 2021b) (see Fig. 11). The decision variables (the inputs between which a decision needs to be made) are applied simultaneously, and the network, after previous training with these decision variables, reaches a state where the population of neurons representing one of the decision variables has a high firing rate (Rolls and Deco 2010; Deco et al. 2013; Rolls 2016c, 2021b). There is noise or randomness in this model of decision-making that is related to the approximately Poisson distributed firing times of neurons for a given mean firing rate. This approach to decision-making (see also Rolls et al. 2010a, b), illustrated in Fig. 11, provides a much more biologically well-founded model with integrate-and-fire neurons coupled in an attractor network than accumulator models of decision-making in which noise is added to two variables to see which one wins (Deco et al. 2013; Shadlen and Kiani 2013).

A key conceptual point can be made here about reward-related decision-making, which will typically be between two or more rewards. The inputs (decision variables, *λ*_1_ and *λ*_2_ in Fig. 11) that drive each of the reward attractor neuronal populations in Fig. 11, need to produce as output the identity of the reward signal, so that behaviour can be directed towards making these goal neurons fire. Effectively, the two sets of output neurons in Fig. 11, each driven by *λ*_1_ and *λ*_2_ in Fig. 11, are the reward neurons, competing with each other through the inhibitory interneurons. This results in the output of the decision-making network being the identity of the reward that won, and that can be used as the goal for behaviour. It is not useful to have a common currency for reward, if common currency means some general reward representation (Cabanac 1992). Instead, the output of the decision-making needs to be the specific reward that won in the computation, and the fact that this is an attractor network provides a way to maintain the firing of the winning neurons so that they can continue firing to act as the goal for the motivated behaviour (Rolls 2014b, 2021b). To place this in the context of emotion: each pleasure associated with each type of reward (with examples in Table 1) must be different, and feel different, so that we know that we have been successful in obtaining the correct reward that was being sought. Of course, having different rewards on the same scale of magnitude is useful, so that the decision-making network weights the two inputs on the same reward value scale (Grabenhorst et al. 2010a).

#### The amygdala

The amygdala in rodents, in which the orbitofrontal cortex is much less developed than in primates (Passingham and Wise 2012; Passingham 2021), has been implicated in emotion-related responses such a conditioned autonomic responses, conditioned freezing behavior, cortical arousal, and learned incentive effects in fear conditioning in which an auditory tone is associated with foot shock (LeDoux 1995, 1996; Quirk et al. 1996). Synaptic modification in the amygdala is implicated in the learning of these types of response (Davis 1992, 1994; Davis et al. 1995; Rogan et al. 1997; LeDoux 2000a, b; Davis 2011). In macaques, bilateral lesions of the amygdala impair the learning of fear-potentiated startle to a visual cue (Antoniadis et al. 2009). In macaques, connections reach the lateral and basal amygdala from the inferior temporal visual cortex, the superior temporal auditory cortex, the cortex of the temporal pole, and the cortex in the superior temporal sulcus (Van Hoesen 1981; Amaral et al. 1992; Ghashghaei and Barbas 2002; Freese and Amaral 2009). The visual and auditory inputs from these cortical regions may be associated in the primate amygdala with primary reinforcers such as taste from the anterior insular primary taste cortex, and with touch and nociceptive input from the insular somatosensory cortex (Leonard et al. 1985; Rolls 2000c, 2014b; Kadohisa et al. 2005a, b; Wilson and Rolls 2005; Rolls et al. 2018). The outputs of the primate amygdala include connections to the hypothalamus, autonomic centres in the medulla oblongata, and ventral striatum (Heimer et al. 1982; Amaral et al. 1992; Freese and Amaral 2009; Rolls 2014b). In addition, the monkey amygdala has direct projections back to many areas of the temporal, orbitofrontal, and insular cortices from which it receives inputs (Amaral et al. 1992), including even V1 (Freese and Amaral 2005), and to the hippocampal system (Stefanacci et al. 1996). In addition, different fMRI responses of the macaque inferior temporal cortex to different face expressions were reduced after amygdala lesions (Hadj-Bouziane et al. 2012).

**Fig. 12:**
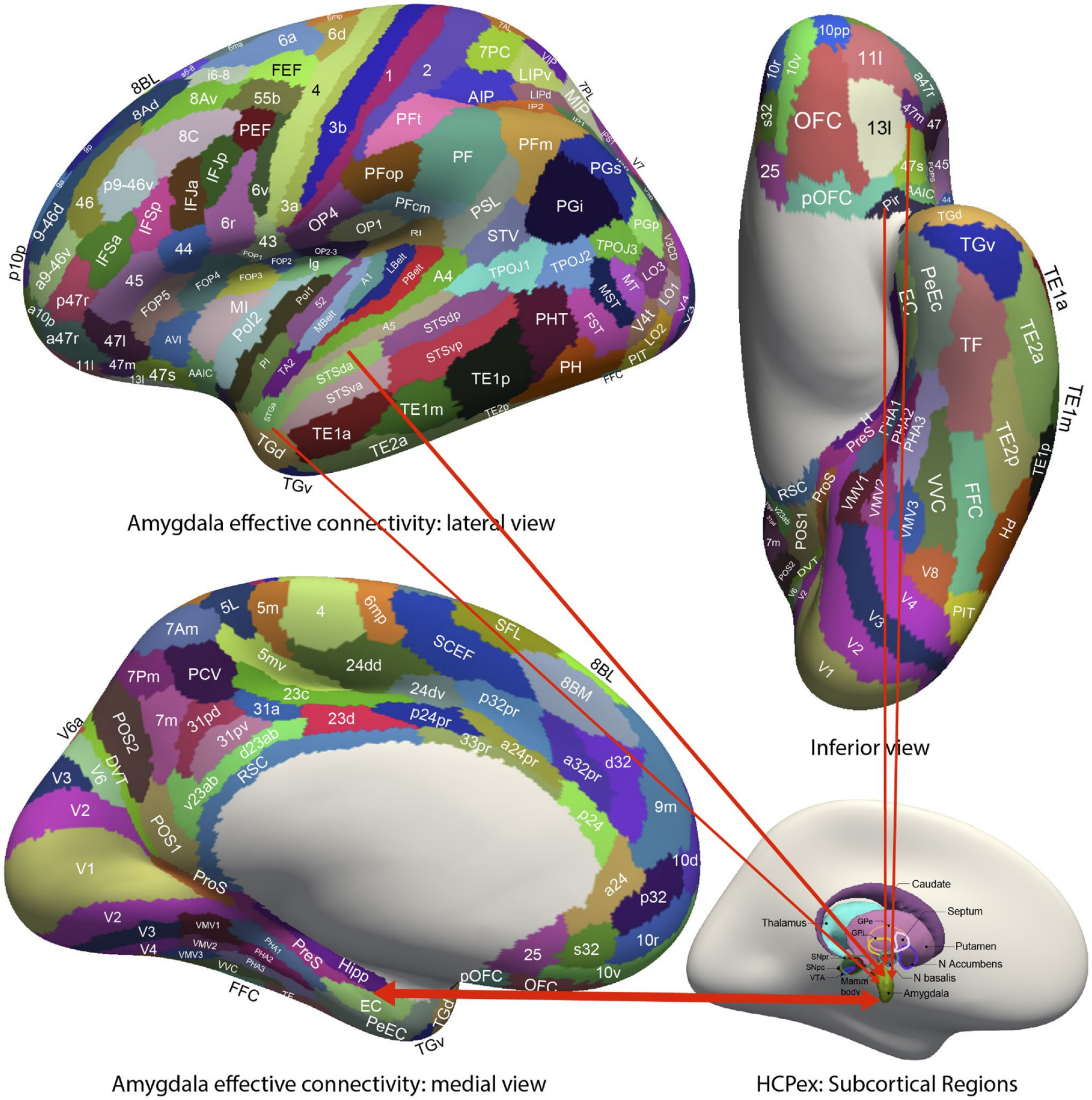
Effective connectivity of the human amygdala: schematic diagram. The width of the arrows reflects the effective connectivity with the size of the arrowheads reflecting the connectivity in each direction. The connectivity from most cortical areas (anterior temporal lobe STGa and TGd, STSda and A5, and pyriform olfactory cortex) is only towards the amygdala. The connectivity with the hippocampal system (Hipp, entorhinal cortex EC, and perirhinal cortex PeEc) is in both directions. The sulci have been opened sufficiently to show the cortical regions in the sulci. The cortical regions are defined in the Human Connectome Project Multi-Modal Parcellation atlas (Glasser et al. 2016a; Huang et al. 2022). The abbreviations are provided elsewhere (Huang et al. 2022; Rolls et al. 2023a)

Although the primate amygdala thus has some of the same connections as the orbitofrontal cortex in monkeys (see Fig. 3) (Rolls 2014b, 2023d), in humans it has much less connectivity with the neocortex than the orbitofrontal cortex (Fig. 12) (Rolls et al. 2023a). In humans, the amygdala receives primarily from auditory cortex A5, and semantic regions in the superior temporal gyrus and temporal pole regions; the piriform (olfactory) cortex; the lateral orbitofrontal cortex 47m; somatosensory cortex; the memory-related hippocampus, entorhinal cortex, perirhinal cortex, and parahippocampal cortex; and from the cholinergic nucleus basalis (Rolls et al. 2023a). The amygdala has effective connectivity to the hippocampus, entorhinal and perirhinal cortex; to the temporal pole; and to the lateral orbitofrontal cortex (Fig. 12) (Rolls et al. 2023a). Given the paucity of amygdalo-neocortical effective connectivity in humans, and the richness of its subcortical outputs in rodents (Quirk et al. 1996) and in humans (Klein-Flugge et al. 2022), it is proposed that the human amygdala is involved primarily in autonomic and conditioned responses via brainstem connectivity, rather than in reported (declarative) emotion (Rolls et al. 2023a).

This new evidence about the connectivity of the human amygdala is consistent with the evidence that the amygdala is an evolutionarily old brain region, and appears to be overshadowed by the orbitofrontal cortex in humans (Rolls 2014b, 2019b, 2021b, c, 2023d; Rolls et al. 2020b). For example, the effects of damage to the human amygdala on emotion and emotional experience are much more subtle (Adolphs et al. 2005; Whalen and Phelps 2009; Delgado et al. 2011; Feinstein et al. 2011; Kennedy and Adolphs 2011; Damasio et al. 2013; LeDoux and Pine 2016; LeDoux et al. 2018; Rolls et al. 2023a) than of damage to the orbitofrontal cortex (Rolls et al. 1994; Hornak et al. 1996, 2003, 2004; Camille et al. 2011; Fellows 2011; Rolls 2019b). Indeed, LeDoux and colleagues have emphasized the evidence that the human amygdala is rather little involved in subjective emotional experience (LeDoux 2012; LeDoux and Pine 2016; LeDoux and Brown 2017; LeDoux et al. 2018; LeDoux 2020; Taschereau-Dumouchel et al. 2022). That is in strong contrast to the orbitofrontal cortex, which is involved in subjective emotional experience, as shown by the evidence just cited. LeDoux’s conundrum is: if not the amygdala for subjective emotional experience, then what (LeDoux 2020)? My answer is: the human orbitofrontal cortex is the key brain region involved in subjective emotion (Rolls 2014b, 2019b, 2023d; Rolls et al. 2023a). Further, consistent with the poor rapid reversal learning found by amygdala neurons (Sanghera et al. 1979; Rolls 2014b, 2021b) compared to orbitofrontal cortex neurons, it has been found that neuronal responses to reinforcement predictive cues in classical conditioning update more rapidly in the macaque orbitofrontal cortex than amygdala, and activity in the orbitofrontal cortex but not the amygdala was modulated by recent reward history (Saez et al. 2017).

The problem of over-interpreting the role of the amygdala in emotion was that rodent studies showed that some responses such as classically conditioned autonomic responses and freezing are elicited by the amygdala with its outputs to brainstem systems, and it was inferred that therefore the amygdala is involved in emotion in the way that it is experienced by humans (LeDoux 1995, 1996, 2000a; Quirk et al. 1996). It turned out later that humans with amygdala damage had similar response-related changes, but little impairment in subjectively experienced and reported emotions (Whalen and Phelps 2009; Delgado et al. 2011; Damasio et al. 2013; LeDoux and Pine 2016; LeDoux et al. 2018; Rolls et al. 2023a). It is important therefore it is argued not to infer subjective reported emotional states in humans from responses such as conditioned autonomic and freezing responses (Rolls et al. 2023a). This dissociation of autonomic response systems from subjectively felt and reported emotions in humans is further evidence against the James-Lange theory of emotion and the related somatic marker hypothesis (Damasio 1994, 1996) (see Rolls (2014b) and the Appendix).

Although as described further below the amygdala may be overshadowed in humans by the orbitofrontal cortex, which has connectivity with the amygdala and that could influence amygdala neuronal responses, it is of interest that in macaques, some amygdala neurons not only respond to faces (Leonard et al. 1985), but also respond to socially relevant stimuli when macaques interact socially (Grabenhorst et al. 2019; Grabenhorst and Schultz 2021).

#### The anterior cingulate cortex

Based on cytoarchitecture, connectivity and function, the anterior cingulate cortex can be divided into a pregenual part (regions s32, a24, p24, p32, and d32 in Fig. 5) that is activated by rewards, and a supracallosal or dorsal part (regions a32pr, a24pr, 33pr, p32pr and p23pr in Fig. 6) activated by punishers and non-reward (Grabenhorst and Rolls 2011; Rolls et al. 2023d), with further background provided in Vogt (2009, 2019).

The human pregenual cingulate cortex is activated by many of the same rewards as the medial orbitofrontal cortex; and the supracallosal anterior cingulate cortex is activated by many of the same punishers, and by non-reward during reward reversal, as the lateral orbitofrontal cortex (Grabenhorst and Rolls 2011; Rolls 2019a, 2021b; Rolls et al. 2020c) (see e.g. Fig. 10a). Thus value representations reach the anterior cingulate cortex (ACC). To provide examples, pain activates an area typically 10–30 mm posterior to and above the most anterior (i.e. pregenual) part of the ACC, in what can be described as the supracallosal (or dorsal) anterior cingulate cortex (Vogt et al. 1996; Vogt and Sikes 2000; Rolls et al. 2003c). Pleasant touch activated the pregenual cingulate cortex (Rolls et al. 2003c; McCabe et al. 2008). Pleasant temperature applied to the hand also produces a linear activation proportional to its subjective pleasantness in the pregenual cingulate cortex (Rolls et al. 2008b). Somatosensory oral stimuli including viscosity and the pleasantness of the texture of fat in the mouth also activate the pregenual cingulate cortex (De Araujo and Rolls 2004; Grabenhorst et al. 2010b). Pleasant (sweet) taste also activates the pregenual cingulate cortex (de Araujo et al. 2003b; De Araujo and Rolls 2004) where attention to pleasantness (Grabenhorst and Rolls 2008) and cognition (Grabenhorst et al. 2008a) also enhances activations. Pleasant odours also activate the pregenual cingulate cortex (Rolls et al. 2003b), and these activations are modulated by word-level top-down cognitive inputs that influence the pleasantness of odours (De Araujo et al. 2005), and also by top-down inputs that produce selective attention to odour pleasantness (Rolls et al. 2008a). Unpleasant odours activate the supracallosal ACC (Rolls et al. 2003b). The pregenual cingulate cortex is also activated by the ‘taste’ of water when it is rewarding because of thirst (de Araujo et al. 2003c), by the flavour of food (Kringelbach et al. 2003), and by monetary reward (O'Doherty et al. 2001). Moreover, the outcome value and the expected value of monetary reward activate the pregenual cingulate cortex (Rolls et al. 2008c). Grabenhorst and Rolls (2011) show the brain sites of some of these activations.

In these investigations, the anterior cingulate activations were linearly related to the subjective pleasantness or unpleasantness of the stimuli, providing evidence that the anterior cingulate cortex represents value on a continuous scale, which is characteristic of what is found in the sending region, the orbitofrontal cortex (Rolls 2019a, b, d,2021b). Moreover, evidence was found that there is a common scale of value in the pregenual cingulate cortex, with the affective pleasantness of taste stimuli and of thermal stimuli delivered to the hand producing identically scaled BOLD activations (Grabenhorst et al. 2010a).

We now consider how these value representations are used in the anterior cingulate cortex (ACC). We start with the evidence that primate orbitofrontal cortex neurons represent value, but not actions or behavioural responses (Thorpe et al. 1983; Padoa-Schioppa and Assad 2006; Grattan and Glimcher 2014; Rolls 2019b, d, 2023d), and therefore project value-related information but not action information to the anterior cingulate cortex. In contrast, there is evidence that the anterior cingulate cortex is involved in associating potential actions with the value of their outcomes, in order to select an action that will lead to the desired goal (Walton et al. 2003; Rushworth et al. 2007, 2011; Grabenhorst and Rolls 2011; Kolling et al. 2016; Morris et al. 2022). Indeed, consistent with its strong connections to motor areas (Morecraft and Tanji 2009), lesions of the ACC impair reward-guided action selection (Kennerley et al. 2006; Rudebeck et al. 2008), in humans the ACC is activated when information about outcomes guides choices (Walton et al. 2004; Morris et al. 2022), and neurons in the ACC encode information about actions, outcomes, and prediction errors for actions (Matsumoto et al. 2007; Luk and Wallis 2009; Kolling et al. 2016). For example, if information about three possible outcomes (different juice rewards) had to be associated with two different actions, information about both specific actions and specific outcomes was encoded by neurons in the ACC (Luk and Wallis 2009).

Given the evidence described above, and the connectivity shown in Fig. 7 (Rolls et al. 2023d), it is now proposed that the part of the anterior cingulate cortex involved in action–outcome learning is the supracallosal (dorsal) part, because this has effective connectivity to premotor cortical areas involved in actions with the body, including the mid-cingulate cortex. The route for value input to reach the supracallosal anterior cingulate cortex appears to be from the pregenual anterior cingulate cortex and medial orbitofrontal cortex (Fig. 7 (Rolls et al. 2023d)). The findings that aversive stimuli including pain activate the supracallosal anterior cingulate cortex may relate to the fact that actions to escape from or avoid aversive, unpleasant, stimuli often involve actions of the body, such as those involved in fight, flight or limb withdrawal (Rolls et al. 2023d). The supracallosal anterior cingulate cortex was also implicated in human action–outcome learning in a learning theory-based analysis (Morris et al. 2022).

Further, given the evidence described above, and the connectivity shown in Fig. 7 (Rolls et al. 2023d), it is now proposed that the pregenual anterior cingulate cortex, which receives from the medial orbitofrontal cortex and ventromedial prefrontal cortex (vmPFC), and connects to the hippocampal system (Rolls et al. 2023d), in part via the memory-related parts of the posterior cingulate cortex (Rolls et al. 2023i), provides a route for affective value to be incorporated into hippocampal system episodic memory (Rolls 2022b, 2023a, c), and also to provide the information about goals that is required for navigation (Rolls 2022b, 2023c; Rolls et al. 2023d). Indeed, it has been pointed out that navigation typically involves multistep routes to reach a goal (Rolls 2021d, 2023c; Rolls et al. 2023d).

Further, the pregenual anterior cingulate cortex has connectivity to the septal region which has cholinergic neurons that project to the hippocampus (Fig. 7) (Rolls et al. 2023d), and this may contribute (Rolls 2022b) to the memory problems that can be present in humans with damage to the anterior cingulate cortex and vmPFC region (Bonnici and Maguire 2018; McCormick et al. 2018; Ciaramelli et al. 2019).

Putting all this evidence together, it appears that the anterior cingulate cortex is a key brain region in emotion, for it provides part of the route via which actions can be learned and guided by the reward/punishment outcomes that are received via the orbitofrontal cortex after a goal-directed action is performed. When instrumental learning under control of the goal was referred to previously, this is the brain region that appears to be involved in this aspect of emotion. This capability requires a computational ability to remember previous actions, which could be implemented by attractor networks in the anterior cingulate cortex, and then to associate the remembered actions with the reward or punishment outcome. However, an interesting new extension to this concept is that while the supracallosal anterior cingulate cortex is implicated in body responses such as an action with the limbs to obtain a goal or avoid a punisher, or, fight, or flight, the pregenual anterior cingulate cortex has outputs to the hippocampal system to enable actions such as navigation to obtain goals, as well as to allow reward information to be incorporated into hippocampal episodic memory, for possible future use in finding goals again (Rolls 2021d, 2022b, 2023c; Rolls et al. 2023d).

Consistent with these concepts that the anterior cingulate cortex is involved in emotion, damage to the human anterior cingulate cortex can produce emotional changes and problems in identifying face and voice emotional expressions (Hornak et al. 2003). Also consistent with inputs from reward systems in the orbitofrontal cortex driving actions via the anterior cingulate cortex, the functional connectivity between the medial orbitofrontal cortex and the anterior cingulate cortex is higher in sensation-seekers (Wan et al. 2020), in risk-takers (Rolls et al. 2022c), and in those with a high BMI that may relate to being over-stimulated by the sensory properties of food (Rolls et al. 2023g).

The subgenual cingulate cortex (area 25), and also the orbitofrontal cortex, may link rewards and punishers to autonomic output (Critchley and Harrison 2013; Rolls 2016d, 2019a; Quadt et al. 2018, 2022). Although it has been argued by Rolls (2014b) that the autonomic system is not normally in a circuit through which behavioural responses are produced (i.e., against the James-Lange and related somatic theories of emotion (Damasio 1996), see Appendix), there may be some influence from effects produced through the endocrine system (and possibly the autonomic system, through which some endocrine responses are controlled) on behaviour (Quadt et al. 2018, 2022), or on the dual emotional and rational systems ("A reasoning, rational, route to action") that control behaviour.

A comparison of my theory of emotion with other theories of emotion is provided in the Appendix, but to maintain the continuity of the argument presented in this paper, I now move to relate my theory of emotion to my theory of motivation.

## A theory of motivation, and brain systems that implement motivation

I now describe Rolls’ theory of motivation, which complements and utilises many of the same brain systems, as Rolls’ theory of emotion described above.

### The outline of a theory of motivation

First, the essence of Rolls’ approach to motivation is described. My definition of motivation is that motivational states are states that are present when rewards and punishers, that is, instrumental reinforcers, are the goals for action (Rolls 2014b, 2016f). A reward is anything for which an animal (and this includes humans) will work. A punisher is anything that an animal will work to escape or avoid, or that will suppress actions on which it is contingent (Rolls 2014b). The force of ‘instrumental’ in this definition is that the motivational states are seen as defining the goals for arbitrary behavioural actions, made to obtain the instrumental reinforcer. This is very different from classical conditioning, in which a response, typically autonomic, may be elicited to a stimulus without any need for an intervening state (Rolls 2014b). The motivational states modulate the reward value of instrumental reinforcers that have particular functions (Rolls 2014b, 2016f). It is important in this definition that the reward values of potential goals are controlled appropriately, with for example factors such as plasma glucose, gastric distension and absorbed food acting to control the reward value of food (Rolls 2016a), and cellular and extracellular dehydration modulating the reward value of water (Rolls et al. 1980a, b; Rolls and Rolls 1981).

An example of a motivational state might thus be a hunger state in which the animal will perform goal-directed actions to obtain the reinforcer or goal. Another example is that the omission or termination of a reward (‘extinction’ and ‘time out’ respectively) can produce a motivational state of frustration, in which the probability of the action may become reduced if no action is possible to regain the reward, or may increase if further motivated attempts are likely to lead to the reward (Rolls 2014b, 2016f).

**Fig. 13:**
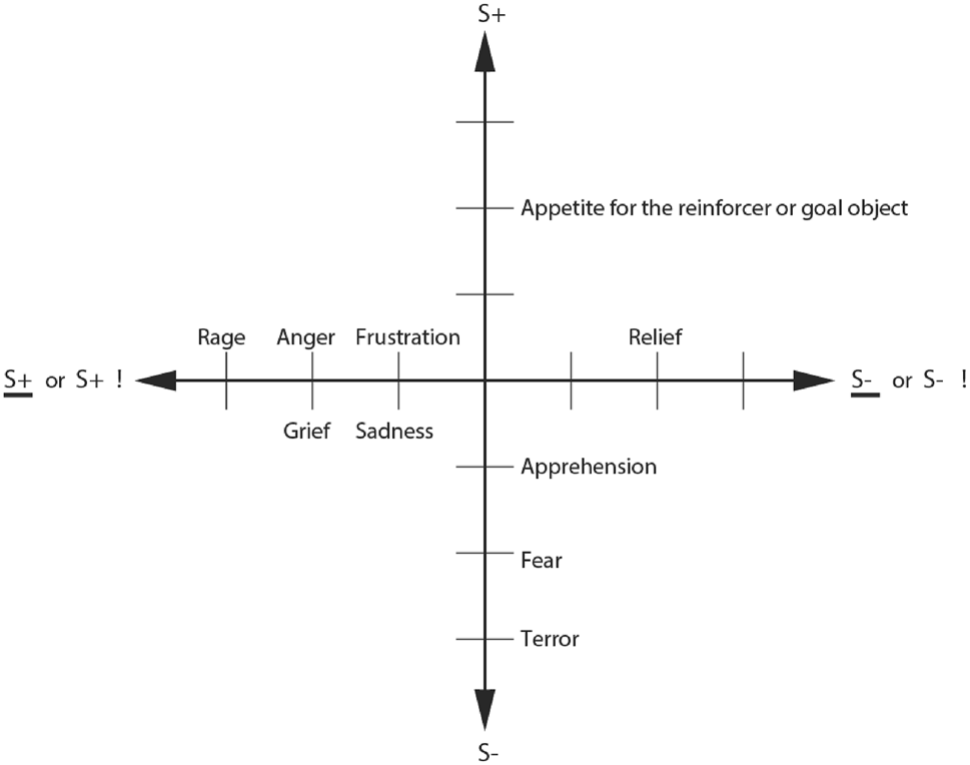
Some of the motivational states associated with different reinforcement contingencies are indicated. Intensity increases away from the centre of the diagram, on a continuous scale. The classification scheme shows how different motivational states created by the different reinforcement contingencies consist of being in a state that leads to (1) performing an action to obtain a reward (S+), (2) performing an action to escape from or avoid a punisher (S−), (3) performing an action, or not, because of the omission of a reward (S+) (extinction) or the termination of a reward (S+!) (time out), and (4) performing an action, or not, because of the omission of a punisher (S−) (avoidance) or the termination of a punisher (S−!) (escape). Note that the vertical axis describes motivational states associated with the delivery of a reward (up) or punisher (down). The horizontal axis describes motivational states associated with the non-delivery of an expected reward (left) or the non-delivery of an expected punisher (right). The diagram summarizes motivational states that might arise for one reinforcer as a result of different contingencies. Every separate reinforcer has the potential to operate according to contingencies such as these. Each different reinforcer will produce different motivational states, but the contingencies will operate as shown to produce different specific motivational states for each different reinforcer

These examples show that the reinforcement contingency as well as the particular reinforcer or goal object (e.g. food, water, aversive stimulation) lead to particular motivational states. The types of motivational state produced by different reinforcement contingencies are illustrated in Fig. 13. The diagram summarizes motivational states that might arise for one reinforcer as a result of different contingencies. Every separate reinforcer has the potential to operate according to contingencies such as these. Each different reinforcer will produce different motivational states, but the contingencies will operate as shown to produce different specific motivational states for each different reinforcer. Thus hunger might be present when the appetite is for the goal object of food, and thirst when the appetite is for the goal object of water. Definitions of reinforcers, and of the contingencies with which they operate, are elaborated by Rolls (2014b).

We must be clear about the difference between motivation and emotion. According to Rolls’ theory of emotion described above, emotion is the state that results from having received, or not having received, the instrumental reinforcer, the goal object (Rolls 2014b, 2018). In contrast, motivation is the state when the instrumental reinforcer is being worked for, before the outcome stage, where the outcome is the delivery or not of the reinforcer. An important attribute of this theory of motivation and emotion is that the goal objects can be the same for motivation and emotion, simplifying the biological specification, with the difference being that motivation is the phase before the outcome, and emotion is the phase after the outcome. An additional property is that emotions, states occurring after the delivery or not of the reinforcer, can be motivating (Rolls 2014b). A good example is that if an expected reward is not obtained, then the frustrative non-reward can be motivating, and make the animal (including humans) work harder to obtain the goal object (Rolls 2014b).

As described above for emotion, reinforcers, that is rewards or punishers, may be unlearned or *primary reinforcers*, or learned, that is secondary reinforcers. An example of a primary reinforcer is pain, which is innately a punisher. The first time a painful stimulus is ever delivered, it will be escaped from, and no learning that it is aversive is needed. Similarly, the first time a sweet taste is delivered, it acts as a positive reinforcer, so it is a primary positive reinforcer or reward. Other stimuli become reinforcing by learning, because of their association with primary reinforcers, thereby becoming ‘*secondary reinforcers*’. For example, the sight of a food that regularly precedes the flavour of the food can rapidly become a secondary reinforcer.

Some examples of primary reinforcers are provided in Table 1. All of the primary reinforcers or goal objects can elicit different, specific, motivational states. As these are primary reinforcers, they are likely to be gene-specified.

### Motivational states are states that modulate the reward or punishment value of instrumental reinforcers, and are different from the mechanisms involved in taxes, approach responses, classical conditioning, and stimulus–response habits

#### Taxes

A simple design principle is to incorporate mechanisms for taxes into the design of organisms. Taxes consist at their simplest of orientation towards stimuli in the environment, for example the bending of a plant towards light that results in maximum light collection by its photosynthetic surfaces. When just turning rather than locomotion is possible, such responses are called tropisms. With locomotion possible, as in animals, taxes include movements toward sources of nutrient, and movements away from hazards such as very high temperatures. The design principle here is that animals have, through a process of natural selection, built receptors for certain dimensions of the wide range of stimuli in the environment, and have linked these receptors to response mechanisms in such a way that the stimuli are approached or escaped from.

#### Habit or stimulus–response learning

In this second level of complexity, involving reinforcers, learning may occur. If an organism performs trial-and-error responses, and as the result of performing one particular response is more likely to obtain a reward, then the response may become linked by a learning process to that stimulus as a result of the reinforcement received. The reward is said to reinforce the response to that stimulus, and we have what is described as stimulus–response or habit learning. The reward acts as a positive reinforcer in that it increases the probability of a response on which it is made contingent. A punisher reduces the probability of a response on which it is made contingent. It should be noted that this is an operational definition, and that there is no implication that the punisher feels like anything in particular—the punisher just has, in the learning mechanism, to reduce the probability of responses followed by the punisher. Stimulus–response or habit learning is typically evident after over-training, and once habits are being executed, the behaviour becomes somewhat independent of the reward value of the goal, as shown in experiments in which the reward is devalued. This is described in more detail in the "Wanting vs liking and goal-directed motivational behaviour". (Stimulus–response habit learning is quite different from action–outcome learning, in which actions are performed and learned to obtain a goal, and in which the value of the goal therefore influences the actions Cardinal et al. 2002; Rolls 2014b).)

Once a behaviour becomes automated as a habit, aversive stimuli can be avoided with very little sign of an emotional state.

The dopamine system is implicated in providing the training signal for this type of habit learning implemented in the striatum (Rolls 2014b, 2021b, 2023d; Schultz 2016a, c).

#### Rewards and punishers: instrumental goals for action towards which motivation is directed

As soon as we have approach to stimuli at one end of a dimension (e.g. a source of nutrient) and away from stimuli at the other end of the dimension (in this case lack of nutrient), we can start to wonder when it is appropriate to introduce the terms ‘rewards’ and ‘punishers’ for the stimuli at the different ends of the dimension. By convention, if an animal’s response consists of a fixed response to obtain the stimulus (e.g. locomotion up a chemical gradient), we shall call this a taxis not a reward. If a fixed behavioural response or action pattern such as skeletomotor freezing and autonomic responses are elicited by a stimulus, they may be adaptive, but are essentially stimulus–response reflexes, with no need for an intervening motivational state, such as the representation of a goal to be reached. On the other hand, if an arbitrary operant action (an instrumental action) can be performed by the animal in order to approach the stimulus or goal, then we will call this rewarded behaviour, and the stimulus that the animal works to obtain a reward, the goal for the action, and the state of wanting and being willing to work for the goal a motivational state. The arbitrary operant response can be thought of as any arbitrary action that the animal will perform to obtain the stimulus. This criterion, of an arbitrary operant response, is often tested by bidirectionality. For example, if a rat can be trained to either raise its tail, or lower its tail, in order to obtain a piece of food, then we can be sure that there is no fixed relationship between the stimulus (e.g. the sight of food) and the response, as there is in a taxis. I, and a number of other authors (Teitelbaum 1974), reserve the term ‘motivated behaviour’ for that in which an arbitrary operant action, an instrumental action, will be performed to obtain a reward or to escape from or avoid a punisher. This is the action–outcome learning described above in which the anterior cingulate cortex is implicated. If this criterion is not met, and only a fixed response can be performed, then the term ‘drive’ can be used to describe the state of the animal when it will work to obtain or escape from the stimulus.

We can thus distinguish a first level of approach/avoidance mechanism complexity in a taxis, with a fixed response available for the stimulus, from a second level of complexity in which any arbitrary response (or action) can be performed, in which case we use the term reward when a stimulus is being approached, and punisher when the action is to escape from or avoid the stimulus. The motivational, intervening, state is one in which the animal will perform an arbitrary action to obtain a goal. Again, we should distinguish habit-related stimulus–response implemented instrumental behaviour, from action-outcome instrumental behaviour that is under the control of the goal.

The role of natural selection in this process is to guide animals to build sensory systems that will respond to dimensions of stimuli in the natural environment along which actions of the animals can lead to better survival to enable genes to be passed on to the next generation, which is what we mean by fitness. Fitness refers to the fitness of genes, but this must be measured by the effects that the genes have on the organism. The animals must be built by such natural selection to perform actions that will enable them to obtain more rewards, that is to work to obtain stimuli that will increase their fitness. Correspondingly, animals must be built to perform actions that will enable them to escape from, or avoid (when learning mechanisms are introduced), stimuli that will reduce their fitness. There are likely to be many dimensions of environmental stimuli along which actions of the animal can alter fitness. Each of these dimensions may be a separate reward-punisher dimension. An example of one of these dimensions might be food reward. It increases fitness to be able to sense nutrient need, to have sensors that respond to the taste of food, and to perform behavioural responses to obtain such reward stimuli when in that need or motivational state. Similarly, another dimension is water reward, in which the taste of water becomes rewarding when there is body-fluid depletion (Rolls 2005).

One aspect of the operation of these reward-punisher systems that these examples illustrate is that with very many reward-punisher dimensions for which actions may be performed, there is a need for a selection mechanism for actions performed to these different dimensions. In this sense, each specific reward and punisher is on a common scale (Grabenhorst et al. 2010a) to facilitate the operation of action selection mechanisms. Evolution must set the magnitudes of each of the different reward systems so that each will be chosen for action in such a way as to maximize overall fitness. Food reward must be chosen as the aim for action if some nutrient depletion is present, but water reward as a target for action must be selected if current water depletion poses a greater threat to fitness than does the current degree of food depletion. This indicates that for a competitive selection process for rewards, each reward must be carefully calibrated in evolution to have the right value on a common scale for the selection process (but not converted into a common currency) (Rolls 2014b). Other types of behaviour, such as sexual behaviour, must be performed sometimes, but probably less frequently, in order to maximize fitness (as measured by gene transmission into the next generation).

There are many processes that contribute to increasing the chances that a wide set of different environmental rewards will be chosen over a period of time, including not only need-related satiety mechanisms that reduce the rewards within a dimension (such as hunger signals described below), but also sensory-specific satiety mechanisms, which facilitate switching to another reward stimulus (sometimes within and sometimes outside of the same main dimension), and attraction to novel stimuli. Attraction to novel stimuli, i.e. finding novel stimuli rewarding, is one way that organisms are encouraged to explore the multidimensional space within which their genes are operating. The suggestion is that animals should be built to find somewhat novel stimuli rewarding, for this encourages them to explore new parts of the environment in which their genes might do better than others’ genes. Unless animals are built to find novelty somewhat rewarding, the multidimensional genetic space being explored by genes in the course of evolution might not find the appropriate environment in which they might do better than others’ genes (Rolls 2014b). The primate orbitofrontal cortex contains neurons that respond to novel stimuli (Rolls et al. 2005).

#### Motivation, and instrumental, action-outcome, goal-directed, learning

When behaviour is under control of the goal, the reward or punisher, then we call this motivated behaviour. A test of whether the behaviour is under the control of the goal is reward devaluation. For example, if humans and other animals are fed to satiety with a food, they show sensory-specific satiety for the food, rate its subjective pleasantness as zero, and are no longer motivated to obtain and ingest it. The motivation for other foods not eaten to satiety usually remains. The hallmark of a devaluation experiment showing that a behaviour is under the control of the goal and therefore qualifies for being described as ‘motivated’ is that when the goal is devalued, the human or other animal will not perform an instrumental action to obtain it the first time that the stimulus is presented (see "Wanting vs liking and goal-directed motivational behaviour").

Two-stages of learning may be involved in such motivational goal-controlled instrumental learning. Rewards and punishers provide the basis for guiding behaviour within a dimension, and for selecting the dimension towards which action should be directed.

The first stage of the learning is stimulus-reinforcer association learning, in which the reinforcing value of a previously neutral, e.g. visual or auditory, stimulus is learned because of its association with a primary reinforcer, such as a sweet or salt taste (Kehoe and Blass 1985) or a painful touch. This learning is of an association between one stimulus, the conditioned or secondary reinforcer, and the primary reinforcer, and is thus stimulus-stimulus association learning. This stimulus-reinforcer learning can be very fast, in as little as one trial. For example, if a new visual stimulus is seen and then placed in the mouth and a sweet taste is obtained, an instrumental action such as reaching for the object will be made on the next trial. Moreover, this stimulus-reinforcer association learning can be reversed very rapidly, at least in primates including humans though not in rodents. For example, if subsequently the object is made to taste of salt, then the visual stimulus is no longer reached for, and the stimulus is even likely to be actively pushed away. This stimulus-reinforcer association learning is implemented in the primate including human orbitofrontal cortex, and leads to representations of expected value (Rolls 2014b, 2018, 2019b, 2021b, 2023d).

The second process or stage in this type of learning is instrumental learning of an action (or ‘operant response’) made in order to obtain the stimulus now associated with reward (or avoid the stimulus associated by learning with the punisher). This is action–outcome learning (implemented in brain regions such as the anterior cingulate cortex as described above Grabenhorst and Rolls 2011; Rushworth et al. 2011; Rolls 2014b, 2019a, 2023d)). The outcome could be a primary reinforcer, but often involves a secondary reinforcer learned by stimulus-reinforcer association learning. The action–outcome learning may be much slower than the stimulus-reinforcer learning, for action–outcome learning may involve trial-and-error learning of which action is successful in enabling the animal to obtain the stimulus now associated with reward or avoid the stimulus now associated with a punisher. However, this second stage may be greatly speeded if an operant response or strategy that has been learned previously to obtain a different type of reward (or avoid a different punisher) can be used to obtain (or avoid) the new stimulus now known to be associated with reinforcement. It is in this flexibility of the action that two-factor learning has a great advantage over stimulus–response learning. The advantage is that any action (even, at its simplest, approach or withdrawal) can be performed once an association has been learned between a stimulus and a primary reinforcer. This flexibility in the action is much more adaptive (and could provide the difference between survival or not) than no learning, as in taxes, or stimulus–response habit learning. The different processes that are involved in instrumental learning are described in more detail by Rolls (2014b).

Another key advantage of this type of two-stage learning is that after the first stage the different rewards and punishers available in an environment can be compared in a selection mechanism, using the common scale of different rewards and punishers for the comparison and selection process (Grabenhorst et al. 2010a; Rolls 2014b). In this type of system, the many dimensions of rewards and punishers are again the basis on which the selection of an action to perform is made (Rolls 2014b).

#### Gene-specified rewards and the mechanisms of evolution

Part of the process of evolution can be seen as identifying the factors or dimensions that affect the (reproductive) fitness of an animal, and providing the animal with sensors that lead to rewards and punishers that are tuned to the environmental dimensions that influence fitness. The example of sweet or salt taste receptors being set up by evolution to provide reward when physiological nutrient need is present (Kehoe and Blass 1985) has been given above, and shows how genes are involved in specifying motivational states.

We can ask whether there would need to be a separate sensing mechanism tuned to provide primary (unlearned) reinforcers for every dimension of the environment to which it may be important to direct motivational behaviour. (The motivated behaviour has to be directed to climb up the reward gradient to obtain the best reward, or to climb a gradient up and away from punishers). It appears that there may not be. For example, in the case of the so-called specific appetites, for perhaps a particular vitamin lacking in the diet, it appears that a type of stimulus-reinforcer association learning may actually be involved, rather than having every possible flavour set up to be a primary reward or punisher. The way that this happens is by a form of association learning. If an animal deficient in one nutrient is fed a food with that nutrient, it turns out that the animal’s physiological state is ‘better’ some time after ingesting the new food, and the animal associates this better physiological state with the taste of that particular food. Later, that food will be chosen. The point here is that the first time the animal is in the deficient state and tastes the new food, that food may not be chosen instead of other foods. It is only after the post-ingestive conditioning that, later, that particular food will be selected (Rozin and Kalat 1971; Berthoud et al. 2021; Rolls 2023e). Thus in addition to a number of specific primary (unlearned) reward systems (e.g. sweet taste for nutrient need, salt taste for salt deficiency (Kehoe and Blass 1985), pain for potentially damaging somatosensory stimulation), there may be great opportunity for other arbitrary sensory stimuli to become conditioned rewards or punishers by association with some quite general change in physiological state. The implication here is that a number of bodily signals can influence a general bodily state, and we learn to improve the general state, rather than to treat the signal as a specific reinforcer that directs us to a particular goal. Another example might be social reinforcers. It would be difficult to build-in a primary reinforcer system for every possible type of social reinforcer. Instead, there may be a number of rather general primary social reinforcers, such as acceptance within a group, approbation, greeting, face expression, and pleasant touch, which are among the primary rewards; and by association with these primary rewards, other stimuli can become secondary social reinforcers.

To help specify the way in which stimulus-reinforcer association learning operates, a list of what may be in at least some species primary reinforcers is provided in Table 1. The reader will doubtless be able to add to this list, and it may be that some of the reinforcers in the list are actually secondary reinforcers. The reinforcers are categorized where possible by modality, to help the list be systematic. Possible dimensions to which each reinforcer is tuned are suggested.

In Rolls’ theories of motivation and emotion, there may be a set of approximately 100 gene-specified primary reinforcers of the type described in Table 1. Each primary reinforcer accounts for a motivational state in which the reward is the target of an instrumental action, and for the emotional state that is produced when the reward or punisher is or is not received. These motivational and emotional states must all be specific; for example hunger must increase food reward but not water reward. These reward value systems must be modulated by the correct selective signals; for example, sensors of metabolic state that relate to hunger must increase the reward value of food but not of water. In so doing, there must be mechanisms to lead animals, when in a motivational state, to navigate and perform appropriate actions to find a specific reward (Deutsch 1960). The reward is produced by the sensory input produced by taste, smell, flavour, touch, sight, and sound, etc., and not by a reduction in the motivational signal. Some of the evidence for this is that very small sensory inputs, such as a drop of food, act as powerful rewards, but reducing hunger by placing food into the stomach produces little reward (Rolls 2014b, 2023e). Consistent with this, reducing the firing of hunger neurons has only a minor rewarding effect (Sternson 2013), so reducing hunger or more generally motivation does not normally act as the reward for instrumental behaviour.

In the reward-based motivational system that I describe, each reward must be scaled to a similar range, so that the different rewards are selected at least sometimes by competing in a decision-making process, so that each reward can contribute to survival and reproductive success (Rolls 2014b). Motivational behaviour can be seen from this approach as an animal operating with a set of initially gene-specified goals for actions (though subject to learned re-evaluation) which compete in a high-dimensional space of rewards for a decision to be taken about which is most rewarding at the time, depending on modulators such as hunger signals, sensory-specific satiety, etc. (Rolls 2014b). The decision taken will also reflect the costs of the actions required to obtain the different rewards (Rolls 2014b). Evidence about how the underlying mechanisms operate are described in *Emotion and Decision-Making Explained* (Rolls 2014b) and elsewhere (Rolls 2018, 2021b, 2023d).

#### Biological economy in the specification of rewards and punishers, for they can be used to implement both motivation and emotion

We now come to the heart of the adaptive value of my approach to motivation and emotion.

My proposal is that the same gene-specified rewards and punishers can be used for both motivation and emotion. This produces great simplification in the genetic specification of motivation and emotion, for the genes have to specify just one set of primary rewards and punishers. The reward has to be motivating, in that animals need to be built to want to perform actions to obtain rewards. Each gene-specified reward then needs to be modulated by the appropriate motivational state. For example, the motivational state of hunger, which modulates the reward value of the taste, smell and sight of food, is signalled by many factors including plasma and gut nutrients and metabolic hormones, as described in detail elsewhere (Rolls 2014b, 2016a, f, 2018). The motivational state of thirst, which modulates the reward value of the taste and sight of water, is signalled by cellular and extracellular fluid volume (Rolls et al. 1980a, b; Rolls and Rolls 1982; de Araujo et al. 2003c; Rolls 2005). Factors that influence the reward value of stimuli involved in sexual behaviour are numerous, and typically adaptive for the genes (Buss 1989, 2015; Rolls 2014b, 2018). For example, in males, the reward value of sexual behaviour typically decreases soon after ejaculation, as a further ejaculate in the same female soon would be unlikely to increase markedly the probability of reproductive success, and it may be adaptive to conserve some sperm for a possible opportunity for reproductive success with another female, with sensory-specific satiety here being referred to as the Coolidge effect (Buss 1989, 2015; Rolls 2014b, 2018). The reward value of sexual behaviour in females is also subject to modulation by many factors that influence reproductive success (Buss 1989, 2015; Rolls 2014b, 2018). The key point here is that the value of each type of reward must be modulated by appropriate motivational signals, such as gut and plasma nutrient signals for food reward, cellular and extracellular volume reductions for water reward, and factors such as the probability of reproductive success in passing on genes to the next generation for sex rewards (Buss 1989, 2015; Rolls 2005, 2014b, 2018).

The same set of rewards, and punishers, when received after for example an instrumental action, lead to emotional states, as described above.

The great utility of both emotional and motivational states relating to rewards and punishers is that this is a highly efficient way for behaviour to be organised, in that the genes specify stimuli that are rewards and punishers, and leave it open to the animal to perform any instrumental action to obtain the reward or avoid the punisher. This is very much more efficient than having genes specify a fixed response to stimuli, such as pecking at small grains as they may be food. The latter type of mechanism of gene-specified responses can have utility for a few responses to a few stimuli, as in the case of chickens pecking at grains of corn. But the genetic specification of many such stimulus–response pairs would be genetically expensive, and would have the great disadvantage that there would be no or little flexibility of the response. Instead, when genes are used to specify rewards and punishers, of the type set out in Table 1, then an almost unlimited set of actions can be learned to obtain the rewards or avoid the punishers. For this reason, I argue that the specification of rewards and punishers by genes, rather than fixed behavioural responses, is a major factor in the design of brains for evolutionary success.

These concepts (including that an important way for genes to influence behaviour is by specifying the reward and punishment value of stimuli) were developed and made clear by Rolls (2005, 2014b, 2016f, 2018), but were not featured in *The Selfish Gene* and subsequent books (Dawkins 1976, 1982, 1986). These concepts are key to understanding how in practice genes frequently increase their (selfish) success by specifying stimuli that are rewards and punishers. Operating in this way, so that the same genes specify rewards and punishers appropriate for both motivation and emotion, and do not specify actions, leads to great adaptiveness and elegance in brain design (Rolls 2016c, f, 2021b, 2023d).

#### Wanting vs liking and goal-directed motivational behaviour

Rolls’ theory of motivation holds that each gene-specified reward is a goal for action, that is, accounts for motivation (Rolls 2016f); and also, when the reward is received, it generates emotion (Rolls 2014b, 2018). An important attribute of these theories of motivation and emotion is that the same specification of a goal object, a reward, perhaps genetically or by stimulus-reward learning, accounts for both the motivation, which has to be produced if the animal is ever to seek the reward, and the emotion, which is associated with the reward when it is received. This makes for great economy in evolution, for genes are needed to specify goal objects, and in doing this, have to produce both working to obtain those goal objects (‘wanting’) and the emotional state when the goal object is received or not received (‘liking’) (Rolls 2014b).

It is useful in this context to discuss an apparent dissociation between ‘wanting’ and ‘liking’ (or ‘desire’ vs ‘pleasure’) that has been raised (Berridge 1996; Berridge and Robinson 1998; Berridge et al. 2009). ‘Wanting’ or conditioned ‘incentive salience’ effects are used to describe classically conditioned approach behaviour to rewards (Berridge and Robinson 1998, 2003), and this learning is implemented via the amygdala and ventral striatum, is under control of dopamine (Cardinal et al. 2002), and contributes to addiction (Robinson and Berridge 2003). Conditioned ‘incentive salience’ effects can influence instrumental responses made, for example, to obtain food.

A first point is that Berridge and Robinson (1998) suggest that ‘liking’ can be measured by orofacial reflexes such as ingesting sweet solutions or rejecting bitter solutions. There is evidence that brain opioid systems are involved in influencing the palatability of and hedonic reactions to foods, in that humans report a reduction in the pleasantness of sucrose solution following administration of naltrexone which blocks opiate receptors, but can still discriminate between sucrose solutions (Gosnell and Levine 2009; Stice et al. 2013). One problem here is that orofacial reflexes may reflect brainstem mechanisms that are not at all closely related to the reward value of food as reflected in instrumental actions performed to obtain food (see Fig. 2). Some of the evidence for this is that these responses occur after decerebration, in which the brainstem is all that remains to control behaviour (Grill and Norgren 1978) [with consistent evidence from anencephalic humans (Steiner et al. 2001)]. Care must be taken about such inferences as there are many routes to behavioural responses (Rolls 2014b, 2021b, 2023d; Balleine 2019) (Fig. 2).

A second point is that normally the rated reward value or pleasantness given in humans to food is closely related to instrumental actions performed to obtain food, as shown by the close relation between pleasantness ratings (‘liking’) by humans given to a food in a sensory-specific satiety experiment, and whether that food is subsequently eaten in a meal (‘wanting’) (Rolls et al. 1981c).

Third, a confusion may arise when a stimulus–response habit is formed by overlearning, and persists even when the reward is devalued by, for example, feeding to satiety. This persistence of stimulus–response habits after reward devaluation should not necessarily be interpreted as ‘wanting’ when not ‘liking’, for it may just reflect the operation of a stimulus–response habit system that produces responses after overlearning without any guidance from reward, pleasantness, and liking (Cardinal et al. 2002; Rolls 2014b; Balleine 2019). Indeed, I emphasize that after overtraining, responses may become inflexibly linked to stimuli, and the goals, and the reward value of the goals, may no longer be directly influencing behaviour in an ongoing way. If behaviour becomes overlearned and a habit or stimulus–response connection is built up by another brain system (such as the basal ganglia), then animals may make automatic responses that are not goal directed (Cardinal et al. 2002; Rolls 2014b; Balleine 2019). There has been considerable confusion in the literature caused by overlooking this point (Berridge and Robinson 1998; Berridge et al. 2009; Berridge and Dayan 2021; Nguyen et al. 2021; Warlow and Berridge 2021). Indeed, just as in the research on the amygdala described above in which LeDoux inferred full emotions from conditioned responses, it is unwarranted and potentially misleading to use subjective emotion-laden words such as ‘wanting’ and ‘liking’ that describe emotional feelings (Berridge 1996; Berridge and Robinson 2003; Robinson and Berridge 2003; Berridge et al. 2009; Berridge and Dayan 2021; Nguyen et al. 2021; Warlow and Berridge 2021), when classically conditioned responses such as Pavlovian-Instrumental Transfer, and orofacial reflexes and stimulus–response habits are what has been measured (Cardinal et al. 2002; Rolls 2014b; Balleine 2019). The fact that behaviour can become stimulus–response and no longer under the control of the goal need not surprise us. Normally, and certainly during learning before habits set in, we want a goal, and when we get the goal we like it: goal stimuli normally specify what is wanted, and what is liked. Indeed, my theory is that normally we want because we like. This is inherent in my theory, for the genes that make a stimulus (such as a sweet taste) rewarding (i.e. wanted, a goal for action) also make the stimulus liked (i.e. accepted, with a subjective correlate of pleasure, pleasantness, and affective liking).

My approach is that I believe that liking, defined by pleasantness ratings of stimuli, is normally very closely related to wanting, that is being willing to perform behaviour (instrumental actions) to obtain a reward of the pleasant stimulus (Rolls 2014b, 2016f). Thus motivational behaviour is normally controlled by reward stimuli or goals (unless the behaviour is overlearned), and motivational state (e.g. hunger) modulates the reward value of unconditioned and conditioned stimuli such as the taste and sight of food. Thus normally, liking a goal object and wanting it are different aspects of how reward systems control instrumental behaviour, and this follows from the approach to gene-specified goal or value representations which in a unifying way account for wanting a goal, and liking the goal object when it is obtained (Rolls 2014b, 2016f, 2018, 2021b, 2023d). For further clarification, consider a probabilistic decision-making task in which the probability *P* of obtaining a reward outcome (such as the taste of food) is 0.5, and the reward outcome value is 1 if the reward is delivered (e.g. fruit juice), and is 0 when the reward is not delivered. Then the Expected (reward) Value when the offer is made is 0.5 (Expected reward Value = *P* × Outcome Value (Rolls 2014b)), and the value of the motivational state at that time (which is *before* the outcome is known) is 0.5. Then later in the trial, the affective/emotional state (which is *after* the outcome is delivered) is 1 for the fruit juice reward, and 0 if nothing is obtained as the outcome.

Thus, it is possible to identify the brain mechanisms involved in ‘wanting’ and ‘liking’ experimentally, and to distinguish them from the classically conditioned ‘incentive salience’ stimuli that influence approach and instrumental actions and which influence ‘appetitive’ behaviour, implemented in part separately from the reward systems that are activated by a primary reinforcer such as the taste of food during ‘consummatory’ behaviour (Cardinal et al. 2002; Rolls 2014b).

## Some implications and extensions of the understanding of emotion, motivation, and their brain mechanisms

### Top-down cognitive effects on reward value and affective responses, for example on the reward value and pleasantness of taste, olfactory, and flavor stimuli

To what extent does cognition influence the reward value of stimuli, and how far down into the sensory system does the cognitive influence reach? Alternatively, is the reward value in brain regions such as the orbitofrontal cortex independent of cognitive factors, with reward value being interfaced to cognition in other, perhaps language-related, brain regions?

We discovered that word-level cognitive effects have top-down modulatory effects on reward value processing in the orbitofrontal cortex and anterior cingulate cortex. This was shown for olfactory (De Araujo et al. 2005), taste (Grabenhorst et al. 2008a), and touch and the sight of touch (McCabe et al. 2008) reward value. For example, a standard test odor (isovaleric acid combined with cheddar cheese odor, presented orthonasally using an olfactometer) was paired with a descriptor word on a screen, which on different trials was “Cheddar cheese” or “Body odor”. Participants rated the affective value of the standard test odor, isovaleric acid, as significantly more pleasant when labelled “Cheddar Cheese” than when labeled “Body odor”, and these effects reflected activations in the medial orbitofrontal cortex and pregenual anterior cingulate cortex (De Araujo et al. 2005). The implication is that cognitive factors can have profound effects on our responses to the reward value and subjective reported pleasantness of olfactory stimuli, in that these effects are manifest quite far down into reward value processing (in the orbitofrontal cortex), so that hedonic representations of odors are affected (De Araujo et al. 2005).

Similar cognitive effects and mechanisms have also been found for the taste and flavor of food, where the cognitive word level descriptor was, for example, ‘rich delicious flavor’ and activations to flavor were increased in the orbitofrontal cortex and regions to which it projects including the pregenual anterior cingulate cortex and ventral striatum, but were not influenced in the insular primary taste cortex where activations reflected the intensity (concentration) of the stimuli (Grabenhorst et al. 2008a).

For the sight of touch, the cognitive modulation was produced by word labels, ‘Rich moisturizing cream’ or ‘Basic cream’, while cream was being applied to the forearm, or was seen being applied to a forearm. The cognitive labels influenced the activations to the sight of touch and also the correlations with pleasantness in the pregenual anterior cingulate/orbitofrontal cortex and ventral striatum (McCabe et al. 2008).

The wider implication of these discoveries is that our cognitive processes can actually modulate the representation of reward value and subjective pleasantness in brain regions involved in reward value representations such as the orbitofrontal and pregenual anterior cingulate cortex, and this can potentially provide important ways in which the appreciation of other rewards such as music, art and aesthetics can be influenced by cognitive factors acting on the reward value representations in parts of the brain that represent reward value and subjective pleasantness. In this way, the appropriate top-down cognitive bias could enhance the pleasure being experienced.

The mechanisms of top-down cognitive modulation are understood as biased activation being applied to the orbitofrontal cortex from brain regions such as the prefrontal cortex which maintain the biasing information in short-term memory (Deco and Rolls 2003, 2004, 2005a, 2005b; Deco et al. 2005; Rolls and Deco 2006, 2010; Rolls 2013a, 2016c, 2021b, 2023d).

### Effects of top-down selective attention to affective value versus intensity on representations of stimuli including those involved in taste, olfactory, and flavour processing

We have found that with taste, flavor, and olfactory food-related stimuli, selective attention to pleasantness modulates representations in the orbitofrontal cortex, whereas selective attention to intensity modulates activations in areas such as the primary taste cortex (Grabenhorst and Rolls 2008, 2010; Rolls et al. 2008a; Ge et al. 2012; Luo et al. 2013; Rolls 2013a).

This differential biasing of brain regions engaged in processing a sensory stimulus depending on whether the cognitive or attentional demand is for affect-related vs more sensory-related processing may be an important aspect of cognition and attention which has implications for how strongly the reward system is driven by food, and thus for eating and the control of appetite, but also for other types of reward (Grabenhorst and Rolls 2008, 2011; Rolls et al. 2008a; Rolls 2012a, 2014b).

The wider implication is that top-down attention directed to the reward value and subjective pleasantness of stimuli can enhance activations to these stimuli in reward-related brain regions, and this has potential applications to enhance the subjective pleasantness of many types of reward, including aesthetic types of reward (e.g. music and art). Attention applied in this way may divert brain systems from maintaining unpleasant ruminating events in memory, and this is of potential use in for example the treatment of depression and other unpleasant states including pain (Rolls 2016b, 2018, 2021b; Rolls et al. 2020b).

The mechanisms of top-down attentional modulation are understood as biased competition and biased activation being applied to the orbitofrontal cortex from brain regions such as the prefrontal cortex which maintain the biasing information in short-term memory (Deco and Rolls 2003, 2004, 2005a, b; Deco et al. 2005; Rolls and Deco 2006, 2010; Luo et al. 2013; Rolls 2013a, 2016c, 2021b, 2023d).

### Individual differences in the reward systems, evolution, and personality

An important hypothesis is that different humans may have reward systems that differ in how strongly each of their reward systems are activated, driven by the sensory and cognitive factors that make stimuli attractive. In a test of this, we showed that activations to the sight and flavor of chocolate in the orbitofrontal and pregenual cingulate cortex were much higher in chocolate cravers than non-cravers (Rolls and McCabe 2007), though there were no differences at the level of the insular taste cortex where taste perception is represented. This provides evidence that differences in specific reward systems, and not necessarily in earlier sensory processing, can lead to individual differences in behaviour to different types of rewarding stimuli. (Rolls 2014b, 2018). This concept that individual differences in responsiveness to food reward are reflected in brain activations in regions related to the control of food intake (Beaver et al. 2006; Rolls and McCabe 2007) may provide a way for understanding and helping to control food intake and obesity (Rolls 2012a, 2014b, 2016a, 2018).

But the concept is much more general than this. The wider implication is that part of the way in which evolution operates is by utilizing natural variation in each of the specific reward systems (examples of which are shown in Table 1), and selecting for reward systems with sensitivities that lead to reproductive success. This results in each individual having a different set of sensitivities of perhaps 100 different gene-specified reward systems of the type shown in Table 1 (Rolls 2014b, 2018).

The sensitivity of an individual to different rewards and punishers, and the ability to learn and be influenced by rewards and punishers, provide a basis for different personalities (Rolls 2014b, 2018). Part of the basis of personality may be differential sensitivity to different rewards and punishers, and omission and termination of different rewards and punishers (the reinforcement contingencies shown in Fig. 1), and this could give rise to many types of personality when we take into account that there are many different types of reinforcer. This can be related to brain function, in that for example the medial orbitofrontal cortex involved in different rewards and the lateral orbitofrontal cortex involved in different punishers and non-rewards could have different sensitivities of these systems for different types of reward. An extreme example might be that if humans were insensitive to social punishers following orbitofrontal cortex damage, we might expect social problems and impulsive behaviour, and indeed Tranel et al (2002) have used the term ‘acquired sociopathy’ to describe some of these patients. Indeed, we might expect sensitivity to different types of reinforcer (including social reinforcers) to vary between individuals both as a result of gene variation and as a result of learning, and this, operating over a large number of different social reinforcers, might produce many different variations of personality based on the sensitivity to a large number of different reinforcers.

Hans J. Eysenck developed the theory that personality might be related to different aspects of conditioning. He analysed the factors that accounted for the variance in the differences between the personality of different humans (using, for example, questionnaires), and suggested that the first two factors in personality (those which accounted for most of the variance) were introversion vs extraversion, and neuroticism (related to a tendency to be anxious). He performed studies of classical conditioning on groups of subjects, and also obtained measures of what he termed arousal. Based on the correlations of these measures with the dimensions identified in the factor analysis, he suggested that introverts showed greater conditionability (to weak stimuli) and are more readily aroused by external stimulation than extraverts; and that neuroticism raises the general intensity of emotional reactions (Eysenck and Eysenck 1968).

Jeffrey Gray (1970) reinterpreted the findings, suggesting that introverts are more sensitive to punishment and frustrative non-reward than are extraverts; and that neuroticism reflects the extent of sensitivity to both reward and punishment. However, in addition extraverts may perform less well at vigilance tasks (in which the subject must detect stimuli that occur with low probability); may tend to be more impulsive; and perform better when arousal is high (e.g. later in the day), and when rapid responses rather than reflective thought is needed (Matthews and Gilliland 1999).

In functional neuroimaging studies, it has been found that happy face expressions are more likely to activate the human amygdala in extraverts than in introverts (Canli et al. 2002). This supports the conceptually important point made above that part of the basis of personality may be differential sensitivity to different rewards and punishers, and omission and termination of different rewards and punishers.

In one update of this approach, links have been made to behavioural economics by relating loss aversion to greater negative valuation sensitivity compared to positive valuation sensitivity; by suggesting that tendencies to approach or avoid have distinct sensitivities to those of the valuation systems; that approach-avoidance conflict is a distinct process from the basic approach and avoidance systems; and linking these to a reinforcer sensitivity theory of personality (Corr and McNaughton 2012).

Another example is the impulsive behaviour that is a part of Borderline Personality Disorder (BPD), which could reflect factors such as less sensitivity to the punishers associated with waiting for rational processing to lead to a satisfactory solution, or changes in internal timing processes that lead to a faster perception of time (Berlin and Rolls 2004; Berlin et al. 2004). It was of considerable interest that the BPD group (mainly self-harming patients), as well as a group of patients with damage to the orbitofrontal cortex, scored highly on a Frontal Behaviour Questionnaire that assessed inappropriate behaviours typical of orbitofrontal cortex patients including disinhibition, social inappropriateness, perseveration, and uncooperativeness. In terms of measures of personality, using the Big Five personality measure (which identifies five major components of personality (Trull and Widiger 2013), both groups were also less open to experience (i.e. less open-minded). In terms of other personality measures and characteristics, the orbitofrontal and BPD patients performed differently: BPD patients were less extraverted and conscientious and more neurotic and emotional than the orbitofrontal group (Berlin and Rolls 2004; Berlin et al. 2004, 2005). Thus some aspects of personality, such as impulsiveness and being less open to experience, but not other aspects, such as extraversion, neuroticism and conscientiousness, were especially related to orbitofrontal cortex function.

However, in terms of detailed understanding of how the computations in different brain regions relate to personality, research is still in its early stages (DeYoung et al. 2022).

### A reasoning, rational, route to action

Routes to action that are related to the emotional system are described in "The neuroscience of emotion in humans and other primates", and are indicated in Fig. 2.

Another main route to action in humans, and perhaps some other species, involves a computation with many "if … then" statements, to implement a multi-step plan to obtain a reward. In this case, the reward may actually be *deferred* as part of the plan, which might involve working first to obtain one reward, and only then to work for a second more highly valued reward, if this was thought to be overall an optimal strategy in terms of resource usage (e.g., time). In this case, syntax is required, because the many symbols (e.g., names of people) that are part of the plan must be correctly linked or bound. Such linking might be of the form: "if A does this, then B is likely to do this, and this will cause C to do this …". The requirement of syntax for this type of planning implies that involvement of language systems in the brain is required for this type of planning (Rolls 2014b, 2018, 2021b, 2023d). Thus the explicit language system in humans may allow working for deferred rewards by enabling use of a one-off, individual, plan appropriate for each situation.

Another building block for such planning operations in the brain may be the type of short-term memory in which the prefrontal cortex is involved. This short-term memory may be for example in non-human primates of where in space a response has just been made. A development of this type of short-term response memory system in humans to enable multiple short-term memories to be held in place correctly, preferably with the temporal order of the different items in the short-term memory coded correctly, may be another building block for the multiple step "if …. then" type of computation in order to form a multiple step plan. Such short-term memories are implemented in the (dorsolateral and inferior convexity) prefrontal cortex of non-human primates and humans (Goldman-Rakic 1996; Fuster 2014; Fuster 2015; Rolls 2016c; Miller et al. 2018; Kolb and Whishaw 2021; Rolls 2021b, 2023d; Rolls et al. 2023e), and may be part of the reason why prefrontal cortex damage impairs planning (Shallice and Cipolotti 2018).

#### Decisions between the emotional and reasoning systems

The question then arises of how decisions are made in animals such as humans that have both the implicit, direct reward-based, and the explicit, rational, planning systems (Rolls 2011, 2014b, 2018, 2019c, 2021b, 2023b). One particular situation in which the first, implicit, system may be especially important is when rapid reactions to stimuli with reward or punishment value must be made, for then the direct connections from structures such as the orbitofrontal cortex to the basal ganglia may allow rapid actions (Rolls 2014b). Another is when there may be too many factors to be taken into account easily by the explicit, rational, planning, system, when the implicit system may be used to guide action. In contrast, when the implicit system continually makes errors, it would then be beneficial for the organism to switch from direct, action based on obtaining what the orbitofrontal cortex system decodes as being the most positively rewarding choice currently available, to the explicit conscious control system which can evaluate with its long-term planning algorithms what action should be performed next. Indeed, it would be adaptive for the explicit system to regularly be assessing performance by the goal-based and habit-based systems, and to switch itself in to control behaviour quite frequently, as otherwise the adaptive value of having the explicit system would be less than optimal.

There may also be a flow of influence from the explicit, verbal system to the implicit system, in that the explicit system may decide on a plan of action or strategy, and exert an influence on the implicit system that will alter the reinforcement evaluations made by and the signals produced by the implicit system (Rolls 2014b, 2018).

It may be expected that there is often a conflict between these systems, in that the first, implicit, emotional, system is able to guide behaviour particularly to obtain the greatest immediate reinforcement, whereas the explicit system can potentially enable immediate rewards to be deferred, and longer-term, multi-step, plans to be formed (Rolls 2019c, 2023b). This type of conflict will occur in animals with a syntactic planning ability, that is in humans and any other animals that have the ability to process a series of "if … then" stages of planning. This is a property of the human language system, and the extent to which it is a property of non-human primates is not yet fully clear. In any case, such conflict may be an important aspect of the operation of at least the human mind, because it is so essential for humans to correctly decide, at every moment, whether to invest in a relationship or a group that may offer long-term benefits, or whether to directly pursue immediate benefits (Rolls 2014b, 2018, 2019c, 2023b).

The thrust of the argument (Rolls 2014b, 2018, 2019c, 2020) thus is that much complex animal including human behaviour can take place using the implicit, emotional, often unconscious, route to action. We should be very careful not to postulate intentional states (i.e., states with intentions, beliefs and desires) unless the evidence for them is strong, and it seems to me that a flexible, one-off, linguistic processing system that can handle propositions is needed for intentional states. What the explicit, linguistic, system does allow is exactly this flexible, one-off, multi-step planning ahead type of computation, which allows us to defer immediate rewards based on such a plan.

#### The selfish gene vs the selfish phene, and evolution

I have provided evidence above that there are two main routes to decision-making and goal-directed action. The first route selects actions by gene-defined goals for action, and is closely associated with emotion. The second route involves multistep planning and reasoning which requires syntactic processing to keep the symbols involved at each step separate from the symbols in different steps (Rolls 2019c, 2020, 2023b, d). (This second route is used by humans and perhaps by closely related animals.) Now the ‘interests’ of the first and second routes to decision-making and action are different. As argued very convincingly by Richard Dawkins in *The Selfish Gene* (Dawkins 1989), and by others (Hamilton 1964, 1996; Ridley 1993), many behaviours occur in the interests of the survival of the genes, not of the individual (nor of the group), and much behaviour can be understood in this way.

I have extended this approach by arguing that an important role for some genes in evolution is to define the goals (i.e. the rewards and punishers) for actions that will lead to better survival of those genes; that emotions are the states associated with these gene-defined goals; and that the defining of goals for actions rather that actions themselves is an efficient way for genes to operate, as it leaves flexibility of choice of action open until the animal is alive (Rolls 2014b, 2018). This provides great simplification of the genotype as action details do not need to be specified, just rewarding and punishing stimuli, and also flexibility of action in the face of changing environments faced by the genes. This is a useful and interesting advance beyond what was considered in *The Selfish Gene* and later books (Dawkins 1976, 1982, 1986, 1989). In any case, the interests that are implied when the first route to action is chosen are those of the “selfish genes”, not those of the individual, the phenotype.

However, the second route to action allows, by reasoning, decisions to be taken that might not be in the interests of the genes, might be longer term decisions, and might be in the interests of the individual, the phenotype (Rolls 2011, 2014b). An example might be a choice not to have children, but instead to devote oneself to science, medicine, music, or literature. The reasoning, rational, system presumably evolved because taking longer-term decisions involving planning rather than choosing a gene-defined goal might be advantageous at least sometimes for genes. But an unforeseen consequence of the evolution of the rational system might be that the decisions would, sometimes, not be to the advantage of any genes in the organism. After all, evolution by natural selection operates utilizing genetic variation like a Blind Watchmaker (Dawkins 1986). In this sense, the interests when the second route to decision-making is used are at least sometimes those of the “selfish phenotype”. (Indeed, we might euphonically say that the interests are those of the “selfish phene” (where the etymology is Gk phaino, ‘appear’, referring to appearance, hence the thing that one observes, the individual. The term ‘phene’ was coined (Rolls 2011, 2014b) to refer to the individual or phenotype, but to emphasize that here we have an individual who can choose between the goals specified by the genes from earlier stages of evolution, and the goals that may be relevant to the reasoning individual who might make a choice using reasoning that might not be in the interests of those emotion-related genes.) Hence the decision-making referred to above is between a first system where the goals are gene-defined, and a second rational system in which the decisions may be made in the interests of the genes, or in the interests of the phenotype and not in the interests of the genes. Thus we may speak of the choice as sometimes being between the “Selfish Genes” and the “Selfish Phene”.

Now what keeps the decision-making between the “Selfish Genes” and the “Selfish Phene” more or less under control and in balance? If the second, rational, system chose too often for the interests of the “Selfish Phene”, the genes in that phenotype would not survive over generations. Having these two systems in the same individual will only be stable if their potency is approximately equal, so that sometimes decisions are made with the first route, and sometimes with the second route. If the two types of decision-making, then, compete with approximately equal potency, and sometimes one is chosen, and sometimes the other, then this is exactly the scenario in which stochastic processes in the decision-making mechanism are likely to play an important role in the decision that is taken (Rolls and Deco 2010; Rolls 2014b, 2016c, 2019c, 2023b). The same decision, even with the same evidence, may not be taken each time a decision is made, because of noise in the system.

The system itself may have some properties that help to keep the system operating well. One is that if the second, rational, system tends to dominate the decision-making too much, the first, gene-based emotional system might fight back over generations of selection, and enhance the magnitude of the reward value specified by the genes, so that emotions might actually become stronger as a consequence of them having to compete in the interests of the selfish genes with the rational decision-making processes.

Another property of the system may be that sometimes the rational system cannot gain all the evidence that would be needed to make a rational choice. Under these circumstances the rational system might fail to make a clear decision, and under these circumstances, basing a decision on the gene-specified emotions is an alternative. Indeed, Damasio (1994) argued that under circumstances such as this, emotions might take an important role in decision-making. In this respect, I agree with him, basing my reasons on the arguments above. He called the emotional feelings gut feelings, and, in contrast to me, hypothesized that actual feedback from the gut was involved. His argument seemed to be that if the decision was too complicated for the rational system, then rely on outputs sent to the viscera, and whatever is sensed by what they send back could be used in the decision-making, and would account for the conscious feelings of the emotional states. My reading of the evidence is that the feedback from the periphery is not necessary for the emotional decision-making, or for the feelings, nor would it be computationally efficient to put the viscera in the loop given that the information starts from the brain, but that is a matter considered in the Appendix and elsewhere (Maia and McClelland 2004; Rolls 2014b, 2018).

Another property of the system is that the interests of the second, rational, system, although involving a different form of computation, should not be too far from those of the gene-defined emotional system, for the arrangement to be stable in evolution by natural selection. One way that this could be facilitated would be if the gene-based goals felt pleasant or unpleasant in the rational system, and in this way contributed to the operation of the second, rational, system. This is something that I propose is the case (Rolls 2011, 2014b, 2018, 2020).

If the multistep syntactic reasoning/planning system fails, there may be a credit assignment problem, in that the faulty step in the series of steps needs to be identified. It has been argued that this computation requires a higher order thought system, which can think about the first order thought (the plan), and can correct it. This higher order thought system needs syntax, as it has to perform computations on the first order syntactic thoughts, the plan. I have proposed that it is a property of this Higher Order Syntactic Thought system that when it operates, it would feel like something, to be thinking about one’s own first order thoughts, and that is a basis for my HOST theory of consciousness (Rolls 2007b, 2008, 2011, 2020). It is suggested that anything that is being dealt with by the HOST computational system becomes conscious, and that is also how qualia, raw sensory feels, are produced (Rolls 2020).

This raises the issue of what the relation is between the mind and the brain (Descartes 1644; Dennett 1991; Block 2005; Carruthers 2019). In the neuroscience-based approach that I propose for the relation between the mind and the brain, the proposal is that events at the sub-neuronal, neuronal, and neuronal network levels take place simultaneously to perform a computation that can be described at a high level as a mental state, with content about the world (Rolls 2021e, f). It is argued that as the processes at the different levels of explanation take place at the same time, they are linked by a non-causal relationship: causality can best be described in brains as operating within but not between levels. This mind-brain theory allows mental events to be different in kind from the mechanistic events that underlie them; but does not lead one to argue that mental events cause brain events, or vice versa: they are different levels of explanation of the operation of the computational system (Rolls 2021e, f). This computational neuroscience levels of explanation approach to causality (Rolls 2023d) provides an opportunity to proceed beyond Cartesian dualism (Descartes 1644) and physical reductionism (Carruthers 2019) in considering the relations between the mind and the brain (Rolls 2020, 2021e, f).

### The dopamine system in a broader context of brain reward systems and emotion

The primate striatum receives from all cortical regions including motor and premotor cortical regions, and is implicated in stimulus–response habit learning, probably by having the ability to associate any stimulus with any response (Rolls 2016c, 2021b, 2023d). Dopamine neurons, by responding to reward prediction errors, are thought to strengthen or weaken particular stimulus–response connections depending on whether the reward outcome is better or worse than predicted (Schultz 2013, 2016b, c, 2017). That hypothesis does not specify where the dopamine neurons receive their reward prediction error input from. It has been proposed that the dopamine system receives its inputs from the orbitofrontal cortex, partly directly, and partly via the ventral striatum and habenula (Rolls 2017), and this hypothesis is supported by the effective connectivity recently demonstrated in humans from the orbitofrontal cortex to the dopaminergic substantia nigra pars compacta (Rolls et al. 2023d) (Fig. 7). The proposal is also supported by the fact that the primate ventral striatum and adjoining part of the head of the caudate nucleus receive connections from the orbitofrontal cortex and amygdala (Haber and Knutson 2010; Rolls 2014b). Consistent with this, some neurons in these striatal regions respond to the taste, flavor, and/or sight of food (Rolls et al. 1983b; Rolls and Williams 1987; Williams et al. 1993; Rolls 2014b; Strait et al. 2015).

One comment is that this dopaminergic system is a small part of the circuitry involved in emotion-related responses described here, as is evident in Fig. 3, where is it shown as part of the habit system, which is not the instrumental system involved in action under control of the goal that is related to emotion in this paper. Other brain systems than the basal ganglia/dopamine system appear to be more involved in goal-dependent actions to obtain rewards and avoid punishers such as the orbitofrontal cortex, vmPFC and anterior cingulate cortex, and which are therefore closely involved in emotion. It is true that there are some dopamine inputs to the orbitofrontal and anterior cingulate cortex, but these regions already have the relevant reward/punisher information, so the roles of dopamine inputs to these regions are not clear. There are also some dopamine inputs to temporal cortex regions, but whether they implement some stamping in of connectivity as proposed for the striatum is not known.

A second comment is that although the striatum receives a dopaminergic input that it has been suggested is a positive reward prediction error signal (Schultz 2013), there may be too much diversity in the activity of dopamine neurons for this to apply in a simple way (Bromberg-Martin et al. 2010; Rolls 2014b, 2023d). Moreover, there is no evidence that the dopamine neurons encode a specific reward signal (for example for the taste of food vs. the texture of fat) in the way that is required to account for the control of goal-directed motivated behaviour and that is present in the primate orbitofrontal cortex (Rolls 2014b). Further, the activity of ventral striatal neurons appears to be more influenced by orbitofrontal cortex types of signals rather than by positive reward prediction error signals (Strait et al. 2015). The role of the striatum and dopamine in the control of behaviour is considered in more detail elsewhere (Rolls 2014b, 2021b, 2023d), but they appear to be much less important than the orbitofrontal cortex and anterior cingulate cortex in emotion, as described here.

### Decision-making and noise in the brain

The attractor model of decision-making is a neuronal network with associatively modifiable recurrent collateral synapses between the neurons of the type prototypical of the cerebral cortex as shown in Fig. 11 (Wang 2002; Rolls and Deco 2010; Rolls 2021b). The decision variables are applied simultaneously, and the network, after previous training with these decision variables, reaches a state where the population of neurons representing one of the decision variables has a high firing rate (Rolls and Deco 2010; Deco et al. 2013; Rolls 2016c, 2021b). There is noise or randomness in this model of decision-making that is related to the approximately Poisson distributed firing times of neurons for a given mean firing rate (Rolls and Deco 2010; Deco et al. 2013; Rolls 2016c, 2021b). It is this noise that makes decision-making probabilistic.

An implication is that if the odds are almost equal, it is wise to take any decision at least 3 times, as noise in the brain might have influenced a single decision. Another implication is that variability in behaviour can be produced by the randomness in this type of decision-making, and this is important for understanding the variability of emotional states, for understanding how decisions are made between the emotional and reasoning systems in our brains, and for understanding many related processes including the advantageous unpredictability of some behaviour, and how creativity is facilitated in the brain by this ‘noise in the brain’ (Rolls and Deco 2010).

### The neurology of human emotion

Some disorders of human emotion produced by brain damage or disease can be understood using the approach taken here (Rolls 2021c). Humans with damage to the orbitofrontal cortex and anterior cingulate cortex fail to reverse correctly in a stimulus-reward reversal task, revealing that they cannot change their behaviour rapidly when the reinforcement contingencies change (Rolls et al. 1994; Hornak et al. 2004; Fellows 2011). These patients also have an impaired ability to identify facial and voice expressions of emotions, and this is likely to contribute to their changes in social behaviour (Hornak et al. 1996, 2003; Tsuchida and Fellows 2012). For these reasons, these patients are often impulsive and disinhibited, have an altered personality, and have impaired subjective feelings of emotion.

In an fMRI study, we showed that the human orbitofrontal cortex is especially involved in pleasant touch and pain, relative to the somatosensory cortex which is more activated by physically strong somatosensory stimuli (Rolls et al. 2003c; Rolls 2010b, 2016e). This raises the issue of where the emotional aspects of pain are represented in the brain. The orbitofrontal cortex, together with the supracallosal anterior cingulate cortex (Vogt and Sikes 2000; Rolls et al. 2003c, 2023d), are thereby implicated in pain processing, and consistent with this, clinical reports provide evidence that patients with orbitofrontal cortex damage know when a painful stimulus is applied, but have a reduced emotion pain reaction. There is room for this evidence on the important contributions of the orbitofrontal cortex in the emotional, affective, subjective, aspects of pain to be incorporated into investigations of pain systems in the brain (Segerdahl et al. 2015; Tracey 2017).

In humans, amygdala damage has much less effect on emotion than does orbitofrontal cortex damage (Rolls 2021c). For example, the effects of damage to the human amygdala on emotion and emotional experience are much more subtle (Whalen and Phelps 2009; Delgado et al. 2011; LeDoux and Pine 2016; LeDoux et al. 2018) than the effects of damage to the orbitofrontal cortex (Rolls et al. 1994; Hornak et al. 1996, 2003, 2004; Camille et al. 2011; Fellows 2011; Rolls 2019b). Indeed, LeDoux and colleagues have emphasized the evidence that the human amygdala is rather little involved in subjective emotional experience (LeDoux and Pine 2016; LeDoux and Brown 2017; LeDoux et al. 2018). This is in strong contrast to the orbitofrontal cortex, which is involved in subjective emotional experience, as described above. The orbitofrontal cortex provides the answer to LeDoux’s conundrum: if not the amygdala for subjective emotional experience, then what? The role of the amygdala in the processing of emotions may be reduced in humans because of the great evolutionary development of the orbitofrontal cortex, which with its cortical design contains attractor networks that are useful in remembering previous emotion-related inputs, and that are useful in decision-making (Rolls 2021c, 2023d).

### A psychiatric disorder of emotion: depression

**Fig. 14:**
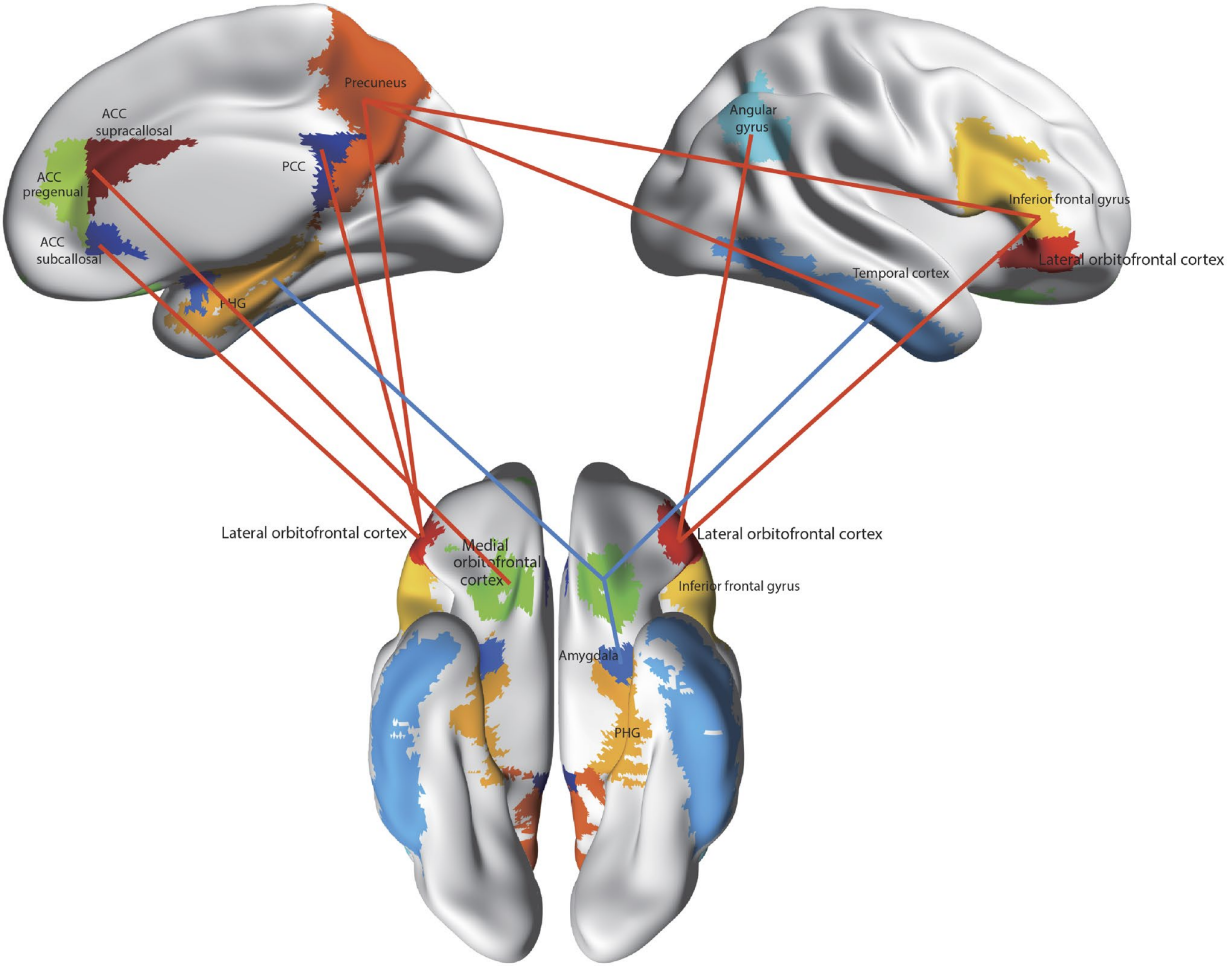
Functional connectivity (FC) differences of the medial and lateral orbitofrontal cortex in major depressive disorder. Higher functional connectivity in depression is shown by red connecting lines, and includes higher functional connectivity of the non-reward/punishment-related lateral orbitofrontal cortex with the precuneus, posterior cingulate cortex (PCC), subcallosal anterior cingulate cortex (ACC), angular gyrus, and inferior frontal gyrus. Lower functional connectivity in depression is shown by blue connecting lines, and includes lower functional connectivity of the medial orbitofrontal cortex with the parahippocampal gyrus memory system (PHG), amygdala, temporal cortex and supracallosal anterior cingulate cortex (ACC). The part of the medial orbitofrontal cortex in which voxels were found with lower functional connectivity in depression is indicated in green. The areas apart from the medial orbitofrontal cortex shown are as defined in the automated anatomical labelling atlas (Rolls et al. 2015), although the investigations that form the basis for the summary were at the voxel level

Not obtaining an expected reward can lead to sadness, and feeling depressed (Rolls 2018; Rolls et al. 2020b) (Fig. 1). The concept is advanced that an important brain region in depression is the orbitofrontal cortex, with depression related to over-responsiveness and over-connectedness of the non-reward-related lateral orbitofrontal cortex, and to under-responsiveness and under-connectivity of the reward-related medial orbitofrontal cortex (Rolls 2016b, 2017, 2019d; Zhang et al. 2023). Evidence from large-scale voxel-level studies and supported by an activation study has been described that provides support for this hypothesis (Rolls 2016b, 2017; Rolls et al. 2020b) (Fig. 14).

Increased functional connectivity of the lateral orbitofrontal cortex with brain areas that include the precuneus and posterior cingulate cortex and angular gyrus is found in patients with depression, and is reduced towards the levels in controls when treated with medication (Cheng et al. 2016, 2018a, 2018b; Rolls et al. 2020a). This is interpreted as related to negative self-esteem and enhanced language-related rumination in depression (Cheng et al. 2016; Rolls et al. 2020b; Zhang et al. 2023).

Decreased functional connectivity of the medial orbitofrontal cortex with medial temporal lobe areas involved in memory is found in patients with depression (Cheng et al. 2016). This is interpreted as being related to less processing or efficacy of systems involved in happy memories in depression (Rolls et al. 2020b; Zhang et al. 2023).

In an activation study with more than 1000 participants, it was found that in individuals with some symptoms of depression, the lateral orbitofrontal cortex has increased sensitivity to not winning, and the medial orbitofrontal cortex has decreased sensitivity to winning in the monetary incentive delay task (Xie et al. 2021). This provides support for Rolls’ theory of depression (Rolls 2016b, 2018; Rolls et al. 2020b).

Some treatments for depression may act by reducing activity or connectivity of the lateral orbitofrontal cortex (Rolls et al. 2020b). New treatments are needed that increase activity or connectivity of the medial orbitofrontal cortex as a possible treatment for depression (Rolls et al. 2020b), and one possibility is that ketamine implements this (Zhang et al. 2023).

These concepts, and that of increased activity in non-reward attractor networks, have potential for advancing our understanding and treatment of depression. Indeed, the hypothesis is developed that the orbitofrontal cortex has a special role in emotion and decision-making and depression in part because as a cortical area and because of its connectivity with other cortical regions it can implement attractor networks useful in maintaining reward and emotional states online including ruminating thoughts, and in decision-making (Rolls et al. 2020b; Rolls 2021b). Maintaining language-related ruminating thoughts because of cortico-cortical feedback loops involving attractor networks may make depressive states particularly severe in humans (Rolls 2016b, 2018).

### Role of reward and emotion in episodic and semantic memory

**Fig. 15:**
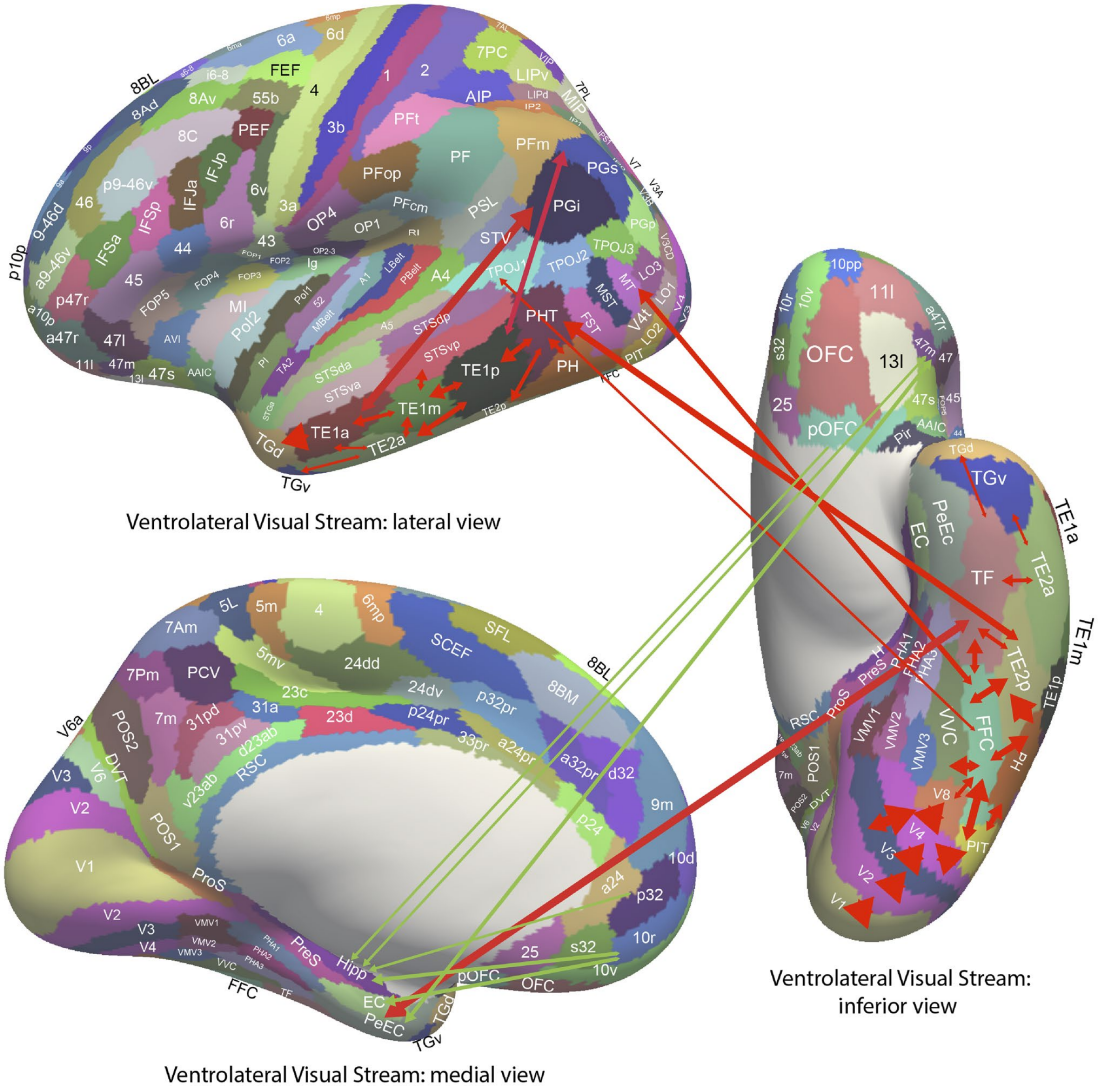
Effective connectivity of the Ventrolateral Visual Stream which reaches inferior temporal cortex TE regions in which objects and faces are represented (red arrows): schematic overview. One of the red arrows shows how the Ventrolateral Visual Stream provides ‘what’ input to the hippocampal memory system via parahippocampal gyrus TF to perirhinal PeEc connectivity from FFC, PH, TE1p, TE2a and TE2p. The green arrows show how reward regions of the orbitofrontal cortex, vmPFC (pOFC, 10r, 10v) and pregenual anterior cingulate (a24 and p32), and punishment/non-reward regions of the lateral orbitofrontal cortex (47m) have effective connectivity with the hippocampus (Hipp), entorhinal cortex (EC), and perirhinal cortex (PeEC). The Ventrolateral Visual Stream also provides input to the semantic language system via TGd. The Ventrolateral Visual Stream also has connectivity to the inferior parietal visual area PFm, PGs and PGi as indicated by 2 green arrows. The widths of the lines and the size of the arrowheads indicate the magnitude and direction of the effective connectivity. After Rolls et al (2023i)

The human orbitofrontal cortex connecting with the vmPFC and anterior cingulate cortex provides a route to the hippocampus for reward and emotional value to be incorporated into episodic memory, enabling memory of where a reward was seen (Rolls 2022b; Rolls et al. 2023b, 2023d) (Figs. 5 and 15). In particular, the green arrows in Fig. 15 show how reward regions of the orbitofrontal cortex, vmPFC (pOFC, 10r, 10v) and pregenual anterior cingulate cortex (a24 and p32), and punishment/non-reward regions of the lateral orbitofrontal cortex (47m) have effective connectivity with the hippocampus, entorhinal cortex, and perirhinal cortex. Consistent with this, some neurons in the primate hippocampus respond to a combination of a spatial view and the reward value that is available at that location in a scene location—reward memory task (Rolls and Xiang 2005). It is argued that reward, punishment, and more generally emotional value are important components of episodic memory (Rolls 2022b).

Beyond this, it is proposed that this reward value component results in primarily episodic memories with some value component to be repeatedly recalled from the hippocampus so that they are more likely to become incorporated into neocortical semantic and autobiographical memories (Rolls 2022b). This is thus a theory of how reward or utility value is important in influencing what information is stored in semantic long-term memory (Rolls 2022b), which is a key aspect of memory consolidation.

The same orbitofrontal and anterior cingulate regions also connect in humans to the septal and basal forebrain cholinergic nuclei (Rolls et al. 2023d) (Fig. 7), which in turn project to the hippocampus and neocortex respectively, where acetylcholine is known to be important in memory consolidation (Hasselmo and Sarter 2011; Zaborszky et al. 2018). It is therefore proposed that key brain systems in the orbitofrontal cortex, vmPFC, and anterior cingulate cortex involved in reward value decoding and emotion play key roles in consolidation of information into hippocampal episodic and also semantic long-term neocortical memory (Rolls 2022b). This also helps to account (Rolls 2022b) for why damage to the vmPFC and anterior cingulate cortex impairs memory (Bonnici and Maguire 2018; McCormick et al. 2018).

#### Brain systems for emotion and motivation in primates including humans compared to those in rodents

**Fig. 16:**
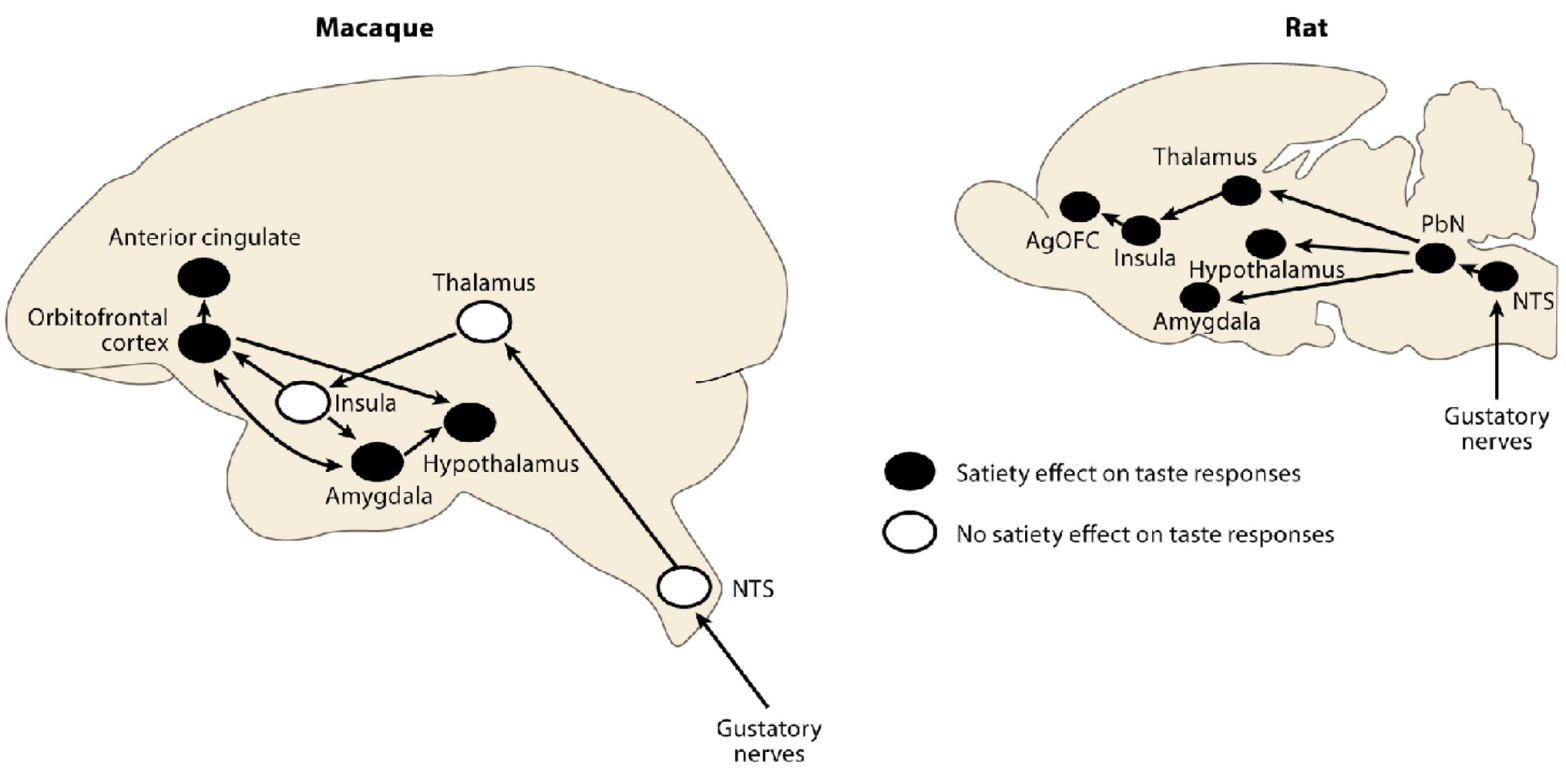
Taste pathways in the macaque and rat. In the *macaque*, gustatory information reaches the nucleus of the solitary tract (NTS), which projects directly to the taste thalamus (ventral posteromedial nucleus, pars parvocellularis, VPMpc) which then projects to the taste cortex in the anterior insula (Insula). The insular taste cortex then projects to the orbitofrontal cortex and amygdala. The orbitofrontal cortex projects taste information to the anterior cingulate cortex. Both the orbitofrontal cortex and the amygdala project to the hypothalamus (and to the ventral striatum). In macaques, feeding to normal self-induced satiety does not decrease the responses of taste neurons in the NTS or taste insula (and by inference not VPMpc) (see text). In the *rat*, in contrast, the NTS projects to a pontine taste area, the parabrachial nucleus (PbN). The PbN then has projections directly to a number of subcortical structures, including the hypothalamus, amygdala, and ventral striatum, thus bypassing thalamo-cortical processing. The PbN in the rat also projects to the taste thalamus (VPMpc), which projects to the rat taste insula. The taste insula in the rat then projects to an agranular orbitofrontal cortex (AgOFC), which probably corresponds to the most posterior part of the primate OFC, which is agranular. (In primates, most of the orbitofrontal cortex is granular cortex, and the rat may have no equivalent to this Wise 2008; Small and Scott 2009; Passingham and Wise 2012; Rolls 2014b, 2015). In the rat, satiety signals such as gastric distension and satiety-related hormones decrease neuronal responses in the NTS (see text), and by inference therefore in the other brain areas with taste-related responses, as indicated in the Figure

Emphasis is placed on research in primates and humans, because there is evidence that the rodent taste and food reward systems operate somewhat differently in primates and humans vs rodents (Rolls 2014b, 2015, 2016d, 2023d). In brief, the taste system is different in rodents in that there is a pontine taste area which then projects subcortically, but in primates there is no pontine taste area and cortical processing is performed first (Fig. 16).

Second, in rodents, the taste and olfactory systems are modulated peripherally [in the nucleus of the solitary tract and the olfactory bulb respectivelyPager et al. 1972; Palouzier-Paulignan et al. 2012; Rolls 2015)] by hunger so that reward is represented peripherally and is entangled with sensory processing, whereas in primates and humans food perception is separated from its reward value, as described elsewhere (Rolls 2014b, 2016d, 2019b, 2021b, 2023d) (Fig. 16). A perceptual correlate of this is that when humans feed to satiety, the intensity of the flavor changes very little, whereas the pleasantness of the flavor decreases to zero (Rolls et al. 1983a; Rolls and Rolls 1997), showing that in humans perceptual representations of taste and olfaction are kept separate from hedonic representations. This is adaptive, in that we do not go blind to the sight, taste, and smell of food after eating it to satiety, and can therefore still learn about where food is located in the environment even when we are not hungry (Rolls 2014b).

Third, the orbitofrontal cortex is very little developed in rodents (with only an agranular part) (Wise 2008), yet is one of the major brain areas involved in taste and olfactory processing, and emotion and motivation, in primates, including humans (Rolls 2014b, 2023d). These findings make the rodent taste and olfactory system a poor model of neural reward processing in humans, and for that reason emphasis is placed here on discoveries in primates and humans (Rolls 2014b, 2015, 2016d, 2019b, 2021b, 2023d). For example, in rodents, movements and actions may be represented in the ‘orbitofrontal cortex’ or what in fact may be the agranular insula (Wilson et al. 2014; Sharpe et al. 2015).

Fourth, the visual system is very different in primates including humans vs rodents, partly because of foveate vision (Rolls 2023a, d). In primates, ventral visual stream object and face recognition become computationally tractable because the fovea provides for fixation in which only one or a very few objects are present, and performs object recognition with a relatively small receptive field in complex natural scenes (Rolls et al. 2003a; Aggelopoulos et al. 2005; Aggelopoulos and Rolls 2005; Rolls 2021b, a, 2023d). In the primate dorsal visual system, there is also great development to provide mechanisms to provide for the fixation of objects in complex natural scenes, and then to perform actions such as reaching and grasping of fixated objects (Itti and Koch 2000; Bisley and Goldberg 2010; Rolls and Webb 2014; Fattori et al. 2017; Galletti and Fattori 2018; Gamberini et al. 2021; Rolls 2023d; Rolls et al. 2023b). Foveate vision has an enormous impact too on other brain systems, including the hippocampal memory system (Rolls 2021b, 2023a, c). There are even differences at a trivial level, in that locomotion greatly increases the firing of rodent visual cortex (Zatka-Haas et al. 2021), whereas the primate inferior temporal visual cortex is little affected by whether an action is performed or not (Rolls et al. 1977, 2003a; Aggelopoulos et al. 2005; Aggelopoulos and Rolls 2005), and instead elegantly represents the identity of the stimulus that is being shown without the great interference described for the rodent. Further, rodents have no posterior cingulate cortex (Vogt 2009; Rolls 2021b). Further, the prefrontal cortex appears to be very different in primates including humans compared to rodents (Passingham 2021).

Fifth, understanding of the functions of different subregions of the rodent orbitofrontal cortex is still limited (Izquierdo 2017; Barreiros et al. 2021).

For these reasons, emphasis here is placed on systems-level investigations in primates and humans, for there is evidence that many cortical systems operate so differently in rodents (Passingham 2021; Rolls 2021b, 2023d). Some of these differences are set out in Sect. 19.10 of Rolls (2023d).

#### Implications for welfare

An implication of the above approach to emotion and motivation is that when considering animal welfare, it is likely to be important to take into account what value each species places on different rewards and the avoidance of possible aversive stimuli. This can in principle be measured by measuring the choices that animals make between different rewards or avoiding different potential punishers. The procedures are well known in neuroeconomics, in which it is possible to measure for example how many drops of fruit juice A are chosen equally often as two drops of fruit juice B (Padoa-Schioppa and Assad 2006; Padoa-Schioppa 2007; Padoa-Schioppa and Assad 2008; Padoa-Schioppa 2011; Padoa-Schioppa and Cai 2011; Cai and Padoa-Schioppa 2012; Glimcher and Fehr 2013; Rolls 2014b; Platt et al. 2016; Padoa-Schioppa and Conen 2017; Yamada et al. 2018; Cai and Padoa-Schioppa 2019; Kuwabara et al. 2020; Dawkins 2021). Similar titration procedures could be used to measure what value, measured by choice, a species places on for example food vs bedding vs having other animals nearby vs overcrowding vs being able to take a swim or shower vs being able to sit on a perch vs being able to reach a branch high above the ground vs being able to perform reproductive behaviour, etc. (Dawkins 2023). When measuring these choices, it is important to ensure that the choice is being made by the goal-directed reward system for instrumental action, and not by any system involved in a reflex or fixed action pattern, or a learned habit.

Measuring instrumental goal-directed choices made by particular species may be useful to minimize over-anthropomorphic inferences about the value that a species may place on different ‘goods’ (the term used in neuroeconomics). Further, even the evidence taken from humans may need to be carefully assessed, for humans are able to provide reasons with their declarative system for their choices made with their syntactic learning system, but may confabulate reasons why they chose a good when the choice has been made by the emotional or by the automatic habit system (Gazzaniga and LeDoux 1978; Rolls 2010a, 2011, 2020, 2023d).

Another implication is that the taste, olfactory and food texture systems present in different species may result in adequate nutrition in their natural environment, but care may be needed to ensure in other environments that the nutrition being made available is appropriate. In this context, it must be remembered that animals do not have flavour mechanisms built to ensure that every possible nutrient needed is being selected by specific appetites for different nutrients. Instead, in the natural environment animals condition to new foods that provide useful nutrients by physiological effects that may occur some time after the food is ingested (Berthoud et al. 2021; Rolls 2023d).

When considering emotional states, we should remember that there are a set of hierarchically organised neural systems that connect what might be defined as emotion-provoking inputs to different types of output, as illustrated in Fig. 2. I define emotional states as states elicited by rewards and punishers that guide goal-directed instrumental behaviour and that are under the control of the reward (or punishment) value of the goal. I refer here therefore to action–outcome learning where the behaviour is under the control of the reward value of the goal in that devaluation of the goal (e.g. by feeding to satiety) results in an immediate cessation of the behaviour, which is not the case for habits or stimulus–response behaviour (Fig. 2). Emotional states are therefore the states involved in implementing this type of goal-dependent instrumental behaviour. We must be very careful to distinguish these emotional states from further states that are related to the subjective feelings, that is to states that in humans are declarative and can be reported and are described as conscious (Rolls 2020). Thus the word ‘emotion’ is ambiguous, and it is always essential to make it clear whether the emotional state is one that might link a stimulus input to for example goal-directed goal-dependent behaviour that utilises action–outcome learning, from a state involved in subjective experience, that may involve further brain systems (Fig. 2). The corresponding situation arises for the word motivation, which as used here and by others refers to goal-directed behaviour of the type just described, with the word ‘drive’ used for simpler behaviors such as approach to food that may not require internal functional states of the type involved in action–outcome learning (Teitelbaum 1974). Thus, for the word ‘motivation’, we should always distinguish systems involved in goal-directed actions, from subjective feelings of being motivated, having ‘desires’.

A set of criteria for achieving good welfare in farm animals, known as the Five Freedoms (Farm Animal Welfare Council 2009) consist of: 1. Freedom from hunger and thirst. 2. Freedom from discomfort. 3. Freedom from pain, injury and disease. 4. Freedom to express normal behaviour. 5. Freedom from fear and distress. The present approach suggests that when assessing (4), it will be useful to measure the value of the different types of ‘normal behaviour’ to help assess priorities. The present approach suggests that when assessing (5), farm animals may often be protected from the fears, stressors, and predators that are present in the natural world, but that these provide a scale against which other fear and distress might be calibrated.

## Conclusions and highlights

1. A new approach is taken here to produce a unified understanding of emotion and motivation and their underlying brain mechanisms. In this unified theory of emotion and motivation, motivational states are states in which instrumental goal-directed actions are performed to obtain rewards or avoid punishers, and emotional states are states that are elicited when the reward or punisher is or is not received. This greatly simplifies our understanding of emotion and motivation, for the same set of genes and associated brain systems can define the primary or unlearned rewards and punishers such as sweet taste or pain that can be used for both emotion and motivation.
2. New evidence on the connectivity in humans of brain systems involved in emotion and motivation is described, which measures the effective connectivity between 360 cortical regions in the Human Connectome Project MultiModal Parcellation atlas (HCP-MMP) (Glasser et al. 2016a), and is complemented by the addition of 66 subcortical regions (Huang et al. 2022). The cortical regions in this atlas are defined by anatomical characteristics (cortical myelin content and cortical thickness), functional connectivity, and task-related fMRI, and provide a useful basis for understanding brain regions with different connectivity and potentially different computational functions. Some of the following points reflect advances in our understanding of brain systems involved in emotion by taking into account the effective connectivity of the human brain, complemented by functional connectivity and diffusion tractography (Rolls et al. 2022a, 2023a, b, d, e, f, i).
3. It is shown that the primate including human orbitofrontal cortex represents primary reinforcers such as taste, pain, and pleasant touch, with this information reaching the orbitofrontal cortex from the primary taste cortex in the anterior insula and from somatosensory cortical regions. It is shown that the primate including human orbitofrontal cortex learns associations between these primary reinforcers and secondary reinforcers such as the sight of food or of an aversive stimulus in one trial, and can reverse these associations in one trial using a rule- or model-based computation. These stimulus-stimulus learned representations are of expected value. The representations in the orbitofrontal cortex are value-based, and are appropriate for being the goals for motivated behaviour, and for eliciting emotional states. Actions are not represented in the primate orbitofrontal cortex. Other inputs to the orbitofrontal cortex are about socially relevant stimuli such as face expression and face identity, and relate to inputs from the cortex in the superior temporal sulcus. Rewards tend to be represented in the human medial orbitofrontal cortex, and punishers and non-reward in the lateral orbitofrontal cortex. This evidence is complemented by the effects of damage to the orbitofrontal cortex in humans, which impairs reward-related reversal learning, emotional responses, and subjective emotional feelings. In primates, reward and punisher value is not represented in cortical stages of sensory processing prior to the orbitofrontal cortex, such as the insular primary taste cortex and inferior temporal visual cortex.
4. The ventromedial prefrontal cortex (vmPFC) receives from the orbitofrontal cortex, is activated by rewards, is implicated in reward-related decision-making, and has connectivity to the pregenual and supracallosal anterior cingulate cortex (Rolls et al. 2023d) (Fig. 7).
5. The human medial and lateral orbitofrontal cortex, and the vmPFC, have connectivity to the pregenual anterior cingulate cortex, which is strongly activated by rewards, and which projects to the hippocampal system, both directly, and via the posterior cingulate cortex (Figs. 5, 6, 7) (Rolls et al. 2023d). It is proposed that this provides the route for rewards and emotional states to become part of episodic memory. It is further proposed that the reward/emotional value of recalled episodic memories is important in influencing which memories are further processed and become incorporated into long-term semantic memory. It is further proposed that this route enables goals for navigation to enter the human hippocampal system, and indeed navigation is almost always to obtain goals, and which are reflected in hippocampal neuronal activity (Rolls 2023a, c).
6. The human pregenual anterior cingulate cortex has effective connectivity to the septum, from which cholinergic neurons important in memory consolidation project to the hippocampus (Fig. 7) (Rolls et al. 2023d). The human medial orbitofrontal cortex (region pOFC) has effective connectivity to the basal forebrain magnocellular nucleus of Meynert, from which cholinergic neurons important in memory consolidation project to the hippocampus (Fig. 7) (Rolls et al. 2023d). It is proposed that by these routes the value system can influence memory consolidation. Consistent with this, damage to the vmPFC/anterior cingulate cortex in humans impairs memory. It is argued that the human orbitofrontal cortex/vmPFC/pregenual anterior cingulate cortex is not a memory system, but a value system, and that this value system influences memory and memory consolidation by these connectivities (Rolls 2022b).
7. The orbitofrontal cortex and pregenual anterior cingulate cortex have connectivity in humans to the supracallosal anterior cingulate cortex, which in turn has connectivity to premotor cortical regions including the midcingulate premotor cortex. It is proposed that these routes provide for action–outcome learning in the supracallosal anterior cingulate cortex, where the outcome is the reward or punisher received from the orbitofrontal cortex and pregenual anterior cingulate cortex.
8. With this foundation, it is proposed that the function of the primate orbitofrontal cortex in emotion is to represent rewards and punishers, and to implement stimulus – reward/punisher association learning and reversal (i.e. stimulus-stimulus learning). It is argued that in contrast, the role of the supracallosal anterior cingulate cortex is to learn associations between actions and the rewards/punishers that follow the actions, and with this action–outcome learning to influence the future choice of actions when reward/aversive expected value stimuli are received from the orbitofrontal cortex.
9. It is shown that the human amygdala has effective connectivity from relatively few cortical regions, primarily those in the anterior temporal lobe, and even less effective connectivity back to the neocortex. The outputs of the human amygdala are directed primarily to brainstem regions involved in autonomic responses, cortical arousal, and some behavioural responses. In line with this, there is evidence that the human amygdala is much less involved in reported, experienced, declarative emotion than the orbitofrontal cortex. This is a key re-evaluation of the functions of the human amygdala in human emotion (Rolls, Deco, Huang and Feng 2023a).
10. It is shown that in addition to these emotion-related outputs to behaviour, in humans and perhaps in other animals there is a rational, reasoning, route to action, that may over-ride the genes selected during evolution to specify the rewards and punishers important in the control of goal-directed behaviour. The reasoning route to action may make choices in the interests of the individual, the phenotype, not in the interests of the gene-specified rewards.
11. Damage to the orbitofrontal cortex in humans can produce neurological changes such as reduced ability to respond correctly to emotion-relevant stimuli such as face and voice expression, and to learn and change behaviour in response to reinforcement contingencies. It is shown that altered connectivity of the orbitofrontal cortex with other brain regions, and sensitivity of the medial orbitofrontal cortex to rewards and of the lateral orbitofrontal cortex to punishers is involved in human depression.
12. In relation to welfare, it is proposed that measurement by choice of the value of goal-related options is important to consider, and needs to be distinguished from other routes to responses such as fixed action patterns, reflexes, taxes, and habits that result from over-training.

## Data Availability

No new data were analysed for this review paper. The effective connectivity, functional connectivity, and diffusion tractography analyses referred to here (Huang et al. 2021; Ma et al. 2022; Rolls et al. 2022a, b, 2023a, b, d, e, f, h, i) were performed with Human Connectome Project data (Glasser et al. 2016b), which are available at the HCP website http://www.humanconnectome.org/. The Human Connectome Project Multimodal Parcellation atlas and its availability are described by Glasser et al. (2016a), and the extended version HCPex is described by Huang et al. (2022), and is available at https://www.oxcns.org.

## Other theories of emotion

For completeness, I now outline some other theories of emotion, and compare them with the above (Rolls’) theory of emotion. Surveys of some of the approaches to emotion that have been taken in the past are provided by Strongman (2003) and Keltner et al. (2018).

### The James-Lange and other bodily theories of emotion including Damasio’s theory

James (1884) believed that emotional experiences were produced by sensing bodily changes, such as changes in heart rate or in skeletal muscles. Lange (1885) had a similar view, although he emphasized the role of autonomic feedback (for example from the heart) in producing the experience of emotion. The theory, which became known as the James-Lange theory, suggested that there are three steps in producing emotional feelings. The first step is elicitation by the emotion-provoking stimulus of peripheral changes, such as skeleto-muscular activity to produce running away, and autonomic changes, such as alteration of heart rate. But, as pointed out above, the theory leaves unanswered perhaps the most important issue in any theory of emotion: Why do some events make us run away (and then feel emotional), whereas others do not? This is a major weakness of this type of theory. The second step is the sensing of the peripheral responses (e.g. running away, and altered heart rate). The third step is elicitation of the emotional feeling in response to the sensed feedback from the periphery.

The history of research into peripheral theories of emotion starts with the fatal flaw that step one (the question of which stimuli elicit emotion-related responses in the first place) leaves unanswered this most important question. The history continues with the accumulation of empirical evidence that has gradually weakened more and more the hypothesis that peripheral responses made during emotional behaviour have anything to do with producing the emotional behaviour (which has largely already been produced anyway according to the James-Lange theory), or the emotional feeling. Some of the landmarks in this history are described by Rolls (2014b).

First, the peripheral changes produced during emotion are not sufficiently distinct to be able to carry the information that would enable one to have subtly different emotional feelings to the vast range of different stimuli that can produce different emotions. The evidence suggests that by measuring many peripheral changes in emotion, such as heart rate, skin conductance, breathing rate, and hormones such as adrenaline and noradrenaline (known in the United States by their Greek names epinephrine and norepinephrine), it may be possible to make coarse distinctions between, for example, anger and fear, but not much finer distinctions (Keltner et al. 2018). Brain processing must of course produce the somewhat different autonomic responses in the first place, and there is evidence that the orbitofrontal and anterior cingulate cortices, perhaps acting via an insular visceral cortex region, are involved in producing autonomic responses (Critchley and Harrison 2013; Quadt et al. 2018). Of course there are pathways from the viscera to the brain, and visceral changes can influence the brain (Critchley and Harrison 2013; Quadt et al. 2018; Quadt, Critchley and Nagai 2022), but whether those visceral changes are in the normal causal chain for the elicitation of emotional states is much more difficult to prove.

Second, when emotions are evoked by imagery, then the peripheral responses are much less marked and distinctive than during emotions produced by external stimuli (Ekman et al. 1983; Levenson et al. 1990). This makes sense in that although an emotion evoked by imagery may be strong, there is no need to produce strong peripheral responses, because no behavioural responses are required.

Third, disruption of peripheral responses and feedback from them either surgically (for example in dogs (Cannon 1927, 1931), or as a result of spinal cord injury in humans (Hohmann 1966; Bermond et al. 1991), does not abolish emotional responses. What was found was that in some patients there was apparently some reduction in emotions in some situations (Hohmann 1966), but this could be related to the fact that some of the patients were severely disabled (which could have produced its own consequences for emotionality), and that in many cases the patients were considerably older than before the spinal cord damage, and this could have been a factor. What was common to both studies was that emotions could be felt by all the patients; and that in some cases, emotions resulting from mental events were even reported as being stronger (Hohmann 1966; Bermond et al. 1991).

Fourth, when autonomic changes are elicited by injections of, for example, adrenaline or noradrenaline, particular emotions are not produced. Instead, the emotion that is produced depends on the cognitive decoding of the reinforcers present in the situation, for example an actor who insults your parents to make you angry, or an actor who plays a game of hula hoop to make you feel happy (Schachter and Singer 1962). In this situation, the hormone adrenaline or noradrenaline can alter the magnitude of the emotion, but not which emotion is felt. This is further evidence that it is the decoded reinforcement value of the input stimulus or events that determines which emotion is felt. The fact that the hormone injections produced some change in the magnitude of an emotion is not very surprising. If you felt your heart pounding for no explicable reason, you might wonder what was happening, and therefore react more or abnormally.

Fifth, if the peripheral changes associated with emotion are blocked with drugs, then this does not block the perception of emotion (Reisenzein 1983).

Sixth, it is found that in normal life, behavioural expressions of emotion (for example smiling when at a bowling alley) do not usually occur when one might be expected to feel happy because of a success, but instead occur when one is looking at one’s friends (Kraut and Johnson 1979). These body responses, which can be very brief, thus often serve the needs of communication, or of action, not of producing emotional feelings.

Despite this rather overwhelming evidence against an important role for body responses in producing emotions or emotional feelings, Damasio (1994) effectively tried to resurrect a weakened version of the James-Lange theory of emotion from the nineteenth century, by arguing with his *somatic marker hypothesis* that after reinforcers have been evaluated, a bodily response (‘somatic marker’) normally occurs, then this leads to a bodily feeling, which in turn is appreciated by the organism to then make a contribution to the decision-making process. [In the James-Lange theory, it was emotional feelings that depend on peripheral feedback; for Damasio, it is the decision of which behavioural response to make that is normally influenced by the peripheral feedback. A quotation from Damasio (1994, p 190) follows: “The squirrel did not really think about his various options and calculate the costs and benefits of each. He saw the cat, was jolted by the body state, and ran.” Here it is clear that the pathway to action uses the body state as part of the route. Damasio would also like decisions to be implemented using the peripheral changes elicited by emotional stimuli. Given all the different reinforcers that may influence behaviour, Damasio (1994) even suggests that the net result of them all is reflected in the net peripheral outcome, and then the brain can sense this net peripheral result, and thus know what decision to take.] The James-Lange theory has a number of major weaknesses just outlined that apply also to the somatic marker hypothesis.

The somatic marker hypothesis postulates that emotional decision-making is facilitated by peripheral feedback from for example the autonomic nervous system. In a direct test of this, emotional decision-making was measured using the Iowa Gambling Task (Bechara, Damasio, Tranel and Damasio 2005) in patients with pure autonomic failure (Heims et al. 2004). In this condition, there is degeneration of the peripheral autonomic system, and thus autonomic responses are severely impaired, and there can be no resulting feedback to the brain. It was found that performance in the Iowa Gambling Task was not impaired, and nor were many other tests of emotion and emotional performance, including face expression identification, theory of mind tasks of social situations, and social cognition tasks. Thus emotional decision-making does not depend on the ongoing feedback from somatic markers related to autonomic function. Damasio might argue that feedback from the autonomic system is not actually important, and that it is feedback from skeletomotor responses such as arm movements or muscle tension that is important. He might also argue that the autonomic feedback is not usually necessary for emotional decision-making, because it can be ‘simulated’ by the rest of the brain. However, the study by Heims et al (2004) does show that ongoing autonomic feedback is not necessary for normal emotional decision-making, and this leaves the somatic marker hypothesis more precarious.

Part of the evidence for the somatic marker hypothesis was that normal participants in the Iowa Gambling Task were described as deciding advantageously before knowing the advantageous strategy (Bechara, Damasio, Tranel and Damasio 1997). The interpretation was that they had implicit (unconscious) knowledge implemented via a somatic marker process that was used in the task, which was not being solved by explicit (conscious) knowledge. Maia and McClelland (2004; 2005) however showed that with more sensitive questioning, normal participants at least had available to them explicit knowledge about the outcomes of the different decks that was as good as or better than the choices made, weakening the arguments that the task was being solved implicitly and using somatic markers (Bechara, Damasio, Tranel and Damasio 1997; Bechara, Damasio, Tranel and Damasio 2005).

Another argument against the somatic marker hypothesis is that there can be dissociations between autonomic and other indices of emotion, thus providing evidence that behaviour may not follow from autonomic and other effects. For example, lesions of different parts of the amygdala influence autonomic responses and instrumental behaviour differently (Rolls 2014b, 2018).

Another major weakness, which applies to both the James-Lange and to Damasio’s somatic marker hypothesis, and to the roles of feedback from the autonomic system to the brain (Quadt, Critchley and Nagai 2022), is that they do not take account of the fact that once an information processor has determined that a response should be made or inhibited based on reinforcement association, a function attributed in Rolls’ theory of emotion (Rolls 2014b, 2018) to the orbitofrontal cortex, it would be very inefficient and noisy to place in the execution route a peripheral response, and transducers to attempt to measure that peripheral response, itself a notoriously difficult procedure. Even for the cases when Damasio (1994) might argue that the peripheral somatic marker and its feedback can be by-passed using conditioning of a representation in, e.g., the somatosensory cortex to a command signal (which might originate in the orbitofrontal cortex), he apparently would still wish to argue that the activity in the somatosensory cortex is important for the emotion to be appreciated or to influence behaviour. (Without this, the somatic marker hypothesis would vanish.) The prediction would apparently be that if an emotional response were produced to a visual stimulus, then this would necessarily involve activity in the somatosensory cortex or other brain region in which the ‘somatic marker’ would be represented. This prediction could be tested (for example in patients with somatosensory cortex damage), but it seems most unlikely that an emotion produced by a visual reinforcer would require activity in the somatosensory cortex to feel emotional or to elicit emotional decisions. However, it has been found that the more damage there is to somatosensory cortex, the greater the impairment in the emotional state reported by patients (Adolphs et al. 2000). However, the parts of the somatosensory system that appear to be damaged most frequently in the patients with emotional change are often in the anterior and ventral extensions of the somatosensory cortex in insular and nearby areas, and it would be useful to know whether this damage interrupted some of the connections or functions of the orbitofrontal cortex areas just anterior.

More recently, Damasio has stated the somatic marker hypothesis in a weak form, suggesting that somatic markers do not even reflect the valence of the reinforcer, but just provide a signal that depends on the intensity of the emotion, independently of the type of emotion. On this view, the role of somatic markers in decision-making would be very general, providing, as Damasio says, just a jolt to spur the system on (A.R. Damasio, paper delivered at the 6th Annual Wisconsin Symposium on Emotion, April 2000).

The alternative view proposed here and elsewhere (Rolls 1990, 2000a, b, 2014b, 2018) is that where the reinforcement value of the visual stimulus is decoded, namely in the orbitofrontal cortex and the amygdala, is the appropriate part of the brain for outputs to influence behaviour (via, e.g., the orbitofrontal to cingulate cortex and orbitofrontal-to-striatal connections), and that the orbitofrontal cortex and amygdala, and brain structures that receive connections from them, are the likely places where neuronal activity is directly related to emotional states.

### Appraisal theory

Appraisal theory (Frijda 1986; Moors et al. 2013; Keltner et al. 2018) generally holds that two types of appraisal are involved in emotion. Primary appraisal holds that “an emotion is usually caused by a person consciously or unconsciously evaluating an event as relevant to a concern (a goal) that is important; the emotion is felt as positive when a concern is advanced and negative when a concern is impeded” (Oatley and Jenkins 1996). The concept of appraisal presumably involves assessment of whether something is a reward or punisher, that is whether it will be worked for or avoided. The description in terms of rewards and punishers adopted here (and by Rolls (2014a) simply seems much more precisely and operationally specified. If primary appraisal is defined with respect to goals, it might be helpful to note that goals may just be the reinforcers specified in Rolls’ theory of emotion (Rolls 1999, 2000a, 2005, 2014a), and if so the reinforcer/punisher approach provides clear definitions of goals. Secondary appraisal is concerned with coping potential, that is with whether for example a plan can be constructed, and how successful it is likely to be.

Scherer (2009) summarizes his appraisal theory approach as follows. He suggests that there are four major appraisal objectives to adaptively react to a salient event:

- Relevance: How relevant is this event for me? Does it directly affect me or my social reference group?
- Implications: What are the implications or consequences of this event and how do they affect my well-being and my immediate or long-term goals?
- Coping Potential: How well can I cope with or adjust to these consequences?
- Normative Significance: What is the significance of this event for my self-concept and for social norms and values?

To attain these objectives, the organism evaluates the event and its consequences on a number of criteria or stimulus evaluation checks, with the results reflecting the organism's subjective assessment (which may well be unrealistic or biased) of consequences and implications on a background of personal needs, goals, and values. Scherer (2009) states that an important feature of the model is that it does not include overt instrumental behaviour. Instead he sees emotion as a reaction to significant events that prepares action readiness and different types of alternative, possibly conflicting, action tendencies but not as a sufficient cause for their execution. This is a clear difference from my theory, in that my theory is that emotions are states that have a key role in brain design by providing a way for stimuli to produce states that are the goals for instrumental actions. Of course stimuli that are instrumental reinforcers, goals for action, can also produce adaptive autonomic and skeletomotor reflexes (such as freezing), but these are responses, and can be classically conditioned, but do not require intervening goal-related representations or states, which are emotional and motivational states.

I note that appraisal theory is in many ways quite close to the theory that I outline here and elsewhere (Rolls 1999, 2000a, 2005, 2014a), and I do not see them as rivals. Instead, I hope that those who have an appraisal theory of emotion will consider whether much of what is encompassed by primary appraisal is not actually rather close to assessing whether stimuli or events are instrumental reinforcers; and whether much of what is encompassed by secondary appraisal is rather close to taking into account the actions that are possible in particular circumstances.

An aspect of some flavours of appraisal theory with which I do not agree is that emotions have as one of their functions releasing particular actions, which seems to make a link with species-specific action tendencies or responses, or ‘fixed action patterns’ (Panksepp 1998, 2011) or more ‘open motor programs’ (Ekman 2003). I argue that rarely are behavioural responses programmed by genes, but instead genes optimize their effects on behaviour if they specify the goals for (flexible) actions, that is if they specify rewards and punishers (Rolls 2014b). The difference is quite considerable, in that specifying goals is much more economical in terms of the information that must be encoded in the genome; and in that specifying goals for actions allows much more flexibility in the actual actions that are produced. Of course I acknowledge that there is some preparedness to learn associations between particular types of secondary and primary reinforcers (Seligman 1970), and see this just as an economy of sensory-sensory convergence in the brain, whereby for example it does not convey much advantage to be able to learn that flashing lights (as contrasted with the taste of a food just eaten) are followed by sickness.

### Panksepp’s theory of emotion

Panksepp’s approach to emotion had its origins in neuroethological investigations of brainstem systems that when activated lead to behaviours like fixed action patterns, including escape, flight and fear behaviour (Panksepp 1998, 2011). Using evidence from brain stimulation that elicits behaviours, he has postulated that there are a set of basic emotions, including for example Seeking, Rage, Fear, Lust, Care, Panic/Grief and Play. He argued that these are ‘natural kinds’, things that exist in nature as opposed to being inventions (constructions) of the human mind. My view is that there are not a few basic emotions, that emotions do not involve fixed action patterns as these do not require intervening emotional states to support goal-directed instrumental actions, and that emotions can be classified based on the specific reinforcer, the specific reinforcement contingency, the actions that are available, etc. as described in Rolls’ theory of emotion (Rolls 2014b, 2018).

### Dimensional, categorical, and other theories of emotion

Dimensional and categorical theories of emotion suggest that there are a number of fundamental or basic emotions. Charles Darwin for example in his book *The Expression of the Emotions in Man and Animals* (Darwin 1872) showed that some basic expressions of emotion are similar in animals and humans. Some of the examples he gave are shown in Table 1. His focus was on the continuity between animals and humans of how emotion is expressed.

In a development of this approach, Ekman (1992; 2003) has suggested that humans categorize face expressions into a number of basic categories that are similar across cultures. These face expression categories include happy, fear, anger, surprise, grief and sadness.

A related approach is to identify a few clusters of variables or factors that result from multidimensional analysis of questionnaires, and to identify these factors as basic emotions. (Multidimensional analyses such as factor analysis seek to identify a few underlying sources of variance to which a large number of data values such as answers to questions are related.)

One potential problem with some of these approaches is that they risk finding seven plus or minus two categories, which is the maximal number of categories with which humans normally operate, as described in a famous paper by George Miller (1956). A second problem is that there is no special reason why the first few factors (which account for most of the variance) in a factor analysis should provide a complete or principled classification of different emotions, or of their functions. In contrast, the theory described here does produce a principled classification of different emotions based on reinforcement contingencies, the nature of the primary and secondary reinforcers, etc., as set out by Rolls (2014b). Moreover, the present theory links the functions of emotions to the classification produced, by showing how the functions of emotion can be understood in terms of the gene-specified reinforcers that produce different emotions (Rolls 2014b, 2018).

An opposite approach to the dimensional or categorical approach is to attempt to describe the richness of every emotion (Ben-Ze'ev 2001). Although it is important to understand the richness of every emotion, I believe that this is better performed with a set of underlying principles of the type set out above and by Rolls (2014b), rather than without any obvious principles to approach the subtlety of emotions.

LeDoux has described a theory of the neural basis of emotion (LeDoux 1992, 1995, 1996, 2012) that is probably conceptually similar to that of Rolls (Rolls 1999, 2000a, 2005, 2014a, 2018) except that he focuses mostly on the role of the amygdala in emotion (and not on other brain regions such as the orbitofrontal cortex, which are poorly developed in the rat); except that he focuses mainly on fear (based on his studies of the role of the amygdala and related structures in fear conditioning in the rat); and except that he suggests from his neurophysiological findings that an important route for conditioned emotional stimuli to influence behaviour is via the subcortical inputs (especially auditory from the medial part of the medial geniculate nucleus of the thalamus) to the amygdala. In contrast, I suggest that cortical processing to the object representation level before the representation is then sent to areas such as the amygdala and orbitofrontal cortex is normally involved in emotion, as emotions normally occur to objects, faces, etc. and not to spots of light or pure tones, which is what are represented precortically. Further, LeDoux (2012) has emphasized especially reflexes and classically conditioned reflexes such as autonomic responses and freezing, which I argue have adaptive value or in LeDoux’s words ‘survival value’, whereas Rolls’ theory is that emotional and motivational states are important intervening states in relation to instrumental actions. The way in which the rodent and amygdala literature has focussed on conditioned responses and not on emotional feelings is described by LeDoux and colleagues (LeDoux, Brown, Pine and Hofmann 2018; LeDoux and Daw 2018; Mobbs et al. 2019; LeDoux 2020; Taschereau-Dumouchel et al. 2022). Indeed, in this more recent research, LeDoux has reached the view that in humans the amygdala is not closely involved in emotional feelings, but does not suggest an alternative. I propose that the key parts of the human brain involved in emotional feelings are the orbitofrontal and anterior cingulate cortex, with the evidence provided above (Rolls 2014b, 2018; Rolls, Deco, Huang and Feng 2023a).

Barrett proposed a theory of constructed emotion (Barrett 2017) in which “the dynamics of the default mode, salience and frontoparietal control networks form the computational core of a brain’s dynamic internal working model of the body in the world, entraining sensory and motor systems to create multi-sensory representations of the world at various time scales from the perspective of someone who has a body, all in the service of allostasis.” “In other words, allostasis (predictively regulating the internal milieu) and interoception (representing the internal milieu) are at the anatomical and functional core of the nervous system (see also (Kleckner et al. 2017)).” Claude Bernard introduced the concept of regulation of the internal milieu (Bernard 1865), and Cannon developed this into the concept of homeostasis (Cannon 1929; Goldstein 2019). Rolls’ theory is that many physiological processes involve regulation, but that emotion is linked to instrumental behavioural actions required to obtain rewards and avoid punishers. Rewards and punishers may be useful for homeostasis, but are also useful for many other functions including for example reproduction, and for example in humans using the reasoning system to perform actions in the interests of phenotypes, which can be other individuals.

Other approaches to emotion are summarized by Keltner et al. (2018).
